# Insight Into the Long Noncoding RNA and mRNA Coexpression Profile in the Human Blood Transcriptome Upon *Leishmania infantum* Infection

**DOI:** 10.3389/fimmu.2022.784463

**Published:** 2022-03-15

**Authors:** Sandra Regina Maruyama, Carlos Alessandro Fuzo, Antonio Edson R. Oliveira, Luana Aparecida Rogerio, Nayore Tamie Takamiya, Gabriela Pessenda, Enaldo Vieira de Melo, Angela Maria da Silva, Amélia Ribeiro Jesus, Vanessa Carregaro, Helder I. Nakaya, Roque Pacheco Almeida, João Santana da Silva

**Affiliations:** ^1^ Department of Genetics and Evolution, Center for Biological Sciences and Health, Federal University of São Carlos, São Carlos, Brazil; ^2^ Department of Clinical Analyses, Toxicology and Food Sciences, Ribeirão Preto School of Pharmaceutics Sciences, University of São Paulo, Ribeirão Preto, Brazil; ^3^ Department of Clinical and Toxicological Analyses, School of Pharmaceutical Sciences, University of São Paulo, São Paulo, Brazil; ^4^ Department of Biochemistry and Immunology, Ribeirão Preto Medical School, University of São Paulo, Ribeirão Preto, Brazil; ^5^ Department of Medicine, University Hospital-Empresa Brasileira de Serviços Hospitalares (EBSERH), Federal University of Sergipe, Aracaju, Brazil; ^6^ Hospital Israelita Albert Einstein, São Paulo, Brazil; ^7^ Fiocruz-Bi-Institutional Translational Medicine Platform, Ribeirão Preto, Brazil

**Keywords:** blood transcriptomics, human visceral leishmaniasis, *Leishmania infantum* (syn. *Leishmania chagasi*), mRNA sequencing (mRNA-seq), long noncoding RNA–mRNA coexpression

## Abstract

Visceral leishmaniasis (VL) is a vector-borne infectious disease that can be potentially fatal if left untreated. In Brazil, it is caused by *Leishmania infantum* parasites. Blood transcriptomics allows us to assess the molecular mechanisms involved in the immunopathological processes of several clinical conditions, namely, parasitic diseases. Here, we performed mRNA sequencing of peripheral blood from patients with visceral leishmaniasis during the active phase of the disease and six months after successful treatment, when the patients were considered clinically cured. To strengthen the study, the RNA-seq data analysis included two other non-diseased groups composed of healthy uninfected volunteers and asymptomatic individuals. We identified thousands of differentially expressed genes between VL patients and non-diseased groups. Overall, pathway analysis corroborated the importance of signaling involving interferons, chemokines, Toll-like receptors and the neutrophil response. Cellular deconvolution of gene expression profiles was able to discriminate cellular subtypes, highlighting the contribution of plasma cells and NK cells in the course of the disease. Beyond the biological processes involved in the immunopathology of VL revealed by the expression of protein coding genes (PCGs), we observed a significant participation of long noncoding RNAs (lncRNAs) in our blood transcriptome dataset. Genome-wide analysis of lncRNAs expression in VL has never been performed. lncRNAs have been considered key regulators of disease progression, mainly in cancers; however, their pattern regulation may also help to understand the complexity and heterogeneity of host immune responses elicited by *L. infantum* infections in humans. Among our findings, we identified lncRNAs such as IL21-AS1, MIR4435-2HG and LINC01501 and coexpressed lncRNA/mRNA pairs such as CA3-AS1/CA1, GASAL1/IFNG and LINC01127/IL1R1-IL1R2. Thus, for the first time, we present an integrated analysis of PCGs and lncRNAs by exploring the lncRNA–mRNA coexpression profile of VL to provide insights into the regulatory gene network involved in the development of this inflammatory and infectious disease.

## Introduction

*Leishmania* protozoans cause a group of diseases known as leishmaniases. The diseases are characterized by a wide range of clinical manifestations depending on the infecting *Leishmania* species, which are classified as cutaneous, mucocutaneous or visceral forms. This dixenous and dimorphic parasite is transmitted to humans through bites from infected sand flies (1). Infections by *Leishmania infantum* can lead to the most severe form of disease, visceral leishmaniasis (VL), which can be lethal if untreated or misdiagnosed (2). Human cases in Brazil account for approximately 95% of reported VL cases in the Americas, with a mortality rate of 7.2% (3). It is also classified as a zoonotic disease with dogs and wild animals as reservoirs. Antileishmanial treatment is administered only parenterally and triggers many toxicity effects, and currently, there is no vaccine against VL. The control of the vector and the surveillance of reservoirs of *L. infantum* have been the measures adopted by the Brazilian public health policies to control and prevent the disease (4). Patients susceptible to VL go through a minimum period of remission of six months, and recidivism has been more frequently observed in recent years. Primarily, children are more often affected, but the incidence in adults has significantly increased (5). However, as observed for other infectious diseases, most infected people do not become sick or even develop any symptoms, likely due to the diverse factors influencing the complexity of the host/parasite interface.

Most mechanistic knowledge about *Leishmania* infections arises from experimental animal models and eventually from human infections. *Leishmania* parasites are able to infect multiple cell types, of which mononuclear phagocytes are the main cells for intracellular replication. By establishing a long-term infection, the parasites are capable of escape from microbicidal and immune mechanisms. CD4^+^ Th1 cells and IFN-γ production are crucial to control *Leishmania* infections, but other CD4^+^ T cell subtypes and CD8^+^ T cells have been shown to be important in the adaptive immune response (6, 7). Displaying a variety of *Leishmania* species, hosts and infection scenarios, the combinations of host/pathogen interactions reach a diversity of host responses to be investigated. In this context, studies aiming to uncover the molecular mechanisms underlying the different outcomes of *L. infantum* human infections are still scarce.

Blood transcriptomics represents an accessible and powerful approach to address the molecular immune mechanisms elicited by inflammatory conditions (8) and to foster the understanding of the heterogeneity of many human infectious diseases. In this regard, blood transcriptomes of human VL caused by *L. donovani* have been explored by others in India (9), with a focus on amphotericin B treatment. Another blood transcriptomics study using patients from Africa also focused on treatment efficacy assessment, but in VL patients coinfected with HIV (10). Of interest for VL occurrence in Brazil, a pioneering study performed by Gardinassi et al. with VL patients infected with *L. infantum* revealed molecular immunological signatures according to the outcome of infection and disease state, such as asymptomatic infection, active infection and during VL remission between two to five months after treatment with pentavalent antimonial (11). These studies found that the IFN-γ response circuit was enriched in active VL (as expected), pathways related to the activation of T lymphocytes *via* MHC class I, type I interferon signaling and B cells (11). Adriaensen et al. showed that IL-10 integrated a 4-gene pre-post transcriptional signature to discriminate treatment outcomes (10).

All these blood transcriptomics studies were dedicated to defining transcriptional signatures of protein-coding genes (PCGs). Another type of gene, as important as PCGs, is those classified as long noncoding RNAs (lncRNAs) owing to their key roles in several molecular processes, such as gene regulation (namely, posttranscriptional and posttranslational mechanisms), genome integrity, cellular structural functions and interference in signaling pathways (12). None of these previous transcriptome studies in VL have focused on lncRNAs. Long noncoding RNAs are broadly expressed in health and disease states, and their specific or altered expression profiles indicate their potential as biomarkers and targets for novel therapies. Most lncRNA functions and relevance came from studies with tumors, but their central role in hematopoiesis and immunity is quite prominent (13, 14).

This type of transcript is larger than 200 nucleotides, and like mRNA, it is spliced, capped at the 5’ end and polyadenylated at the 3’ end (15), i.e., it can be captured not only by total RNA sequencing but also by mRNA sequencing. From this perspective, we performed an integrated analysis of lncRNAs and mRNAs (PCGs) in the blood transcriptomes of human VL caused by *L. infantum* obtained by mRNA sequencing. To produce robust findings and overcome potential host genetics factors, we compared transcriptional data of the same patients in two defined states, during active VL and after six months of being treated, when they were considered clinically cured. In addition, we compared the gene expression profiles of active VL to two other non-diseased profiles, asymptomatic individuals and healthy uninfected volunteers. First, we provided an overview of this new blood transcriptome in VL depicting the most enriched biological pathways and the featured landscape of expressed lncRNAs. Then, we proceeded to the differential expression analysis to define gene subsets to be further focused on in lncRNA–mRNA coexpression analysis. We provided an expression profile of lncRNAs induced in VL during *L. infantum* infection associated with coexpressed protein coding genes, uncovering important insights into the transcriptional response of this parasitic infectious disease. Several lncRNAs were identified as key players in human *L. infantum* infections, and their potential as blood biomarkers for VL is discussed.

## Material and Methods

### Patients and Healthy Uninfected Subjects

**Figure 1:**
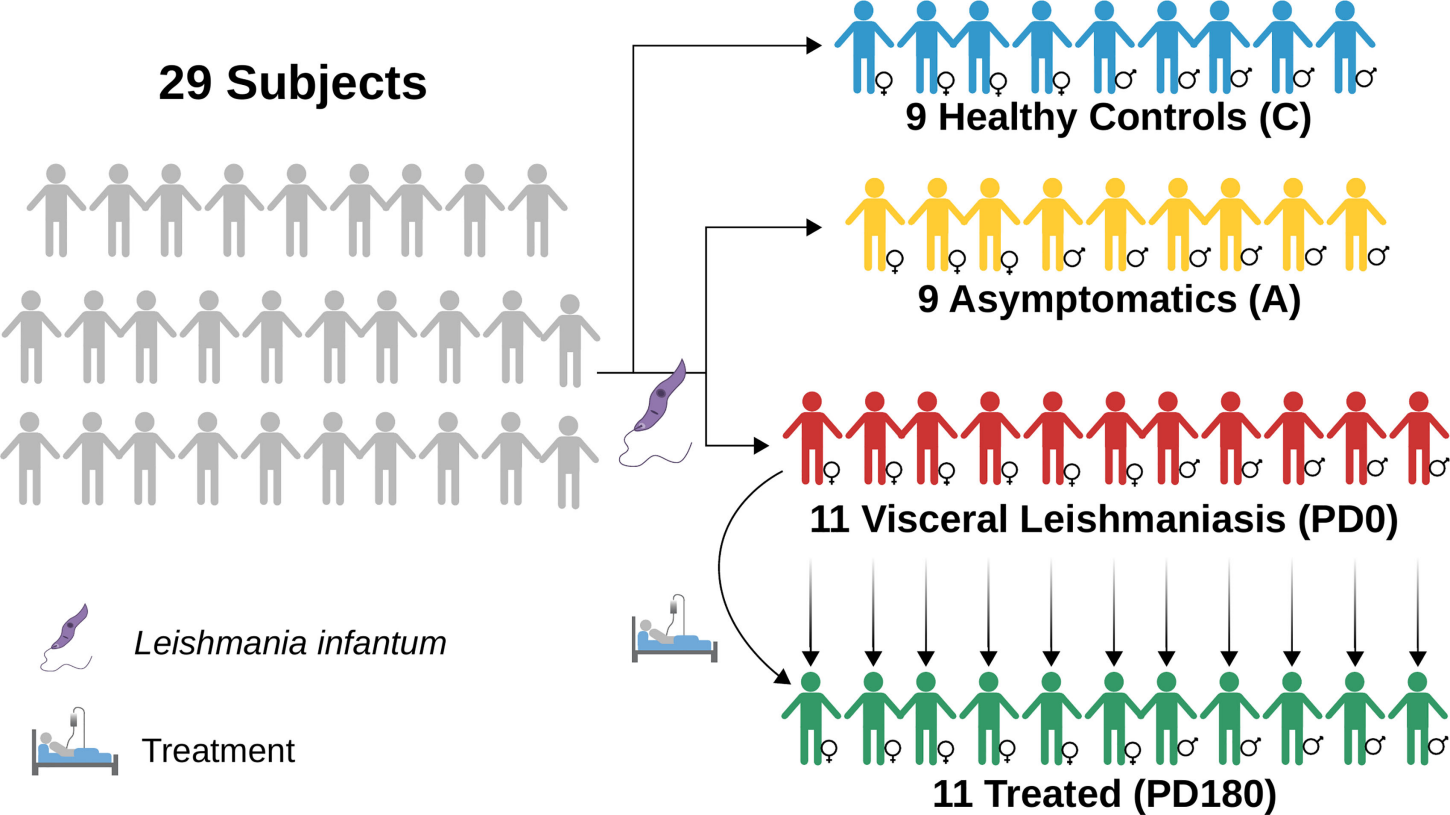
Diagram depicting the groups used in this work to perform RNA-seq data analyses of blood transcriptomes from VL patients (PD0, in red) compared to nondiseased groups, treated (PD180 in green, cured patients), asymptomatic subjects IgG^+^ for *Leishmania infantum* (A, in yellow) and healthy/control subjects (C, in blue). Image diagrammed in Inkscape (https://inkscape.org/).

Twenty-nine individuals were enrolled in the study and categorized into four groups: visceral leishmaniasis patients with active disease (PD0), cured VL patients (PD180; VL patients from the PD0 group 180 days after treatment), asymptomatic individuals (A) and healthy uninfected controls (C), as summarized in **Figure 1** and **Table 1**. The individuals enrolled in this study are from Sergipe state, located at the Northeast region of Brazil, that is not endemic for Malaria. All procedures were performed in accordance with the guidelines of the Brazilian Human Research Ethics Evaluation System (CEP/CONEP) and were approved by the Ethics Committee of the Federal University of Sergipe (CAAE: 04587312.2.0000.0058). All subjects or their legal guardians signed an informed consent form prior to the study.

**Table 1:** General information of the groups analyzed in this study.

Group	N°	Women			Men		
		N°	Age*^b,c^*	Drug therapy *^d^*	N°	Age*^b,c^*	Drug therapy
PD0 *^a^* (VL)	11	6	14.5 (01/51)	Glucantime + AmBisome (n = 2); AmBisome (n = 3)	5	24 (10/44)	Glucantime + AmBisome (n = 2); AmBisome (n = 2); Glucantime (n = 1)
PD180 *^a^* (cured VL)	11	6	14.5 (01/51)		5	24 (10/44)	
Asymptomatic	9	3	12 (03/30)	–	6	21 (06/42)	–
Healthy Control	9	4	25.5 (24/27)	–	5	23 (11/29)	–

a Same patients before (diseased) and after treatment (cured).

b Ages are expressed as the mean values (in years) and minimum and maximum values in parentheses (min/max).

c Age variance among groups was not statistically significant (Kruskal–Wallis test, p-value = 0.5157).

d Not available for one patient.

Diseased patients (PD0 samples) were characterized by the presence of fever, weight loss, hepatosplenomegaly, and low leukocyte and platelet counts. VL diagnosis in the PD0 group was confirmed by direct observation of *Leishmania* in bone marrow aspirate or positive culture in Novy–MacNeal–Nicolle (NNN) medium plus a positive rK39 serological test (Kalazar Detect Rapid Test, InBios International Inc., Seattle, WA). All VL patients were negative for hepatitis B and C viruses and HIV, and also for bacterial infections or other parasites. The patients were treated with conventional drug therapies used for visceral leishmaniasis, according to the national guidelines of the Brazilian Ministry of Health: meglumine antimonate (Glucantime®) and/or liposomal amphotericin B (AmBisome®). In the follow-up appointment, after 180 days of VL treatment, the patients were considered clinically cured, comprising the PD180 group (totaling 22 paired samples, n = 11 in each time point). Healthy individuals who presented normal hematologic indices and neither clinical signs nor symptoms of VL but positive reactions to leishmanial antigens (Montenegro Skin test and rK39 serological test) were considered asymptomatic (n = 9), i.e., they were infected by *L. infantum* but without development of the disease. Parasite visualization tests were not performed in the PD180 group or asymptomatic individuals. Healthy individuals with negative tests for leishmanial antigens comprised the control group (n = 9).

### Blood Sample Collection and RNA Isolation

Peripheral blood samples were collected using BD Vacutainer^®^ tubes for hematologic tests and PAXgene Blood RNA tubes for RNA isolation. Total RNA was extracted from whole blood with the PAXgene Blood RNA Kit followed by globin mRNA depletion using the GLOBINclear™ Human Kit to enrich the samples for RNA from leukocytes. RNA samples were checked for purity by absorbance measurements (nm) of the 260/280 and 260/230 ratios using a NanoDrop™ 1000 Spectrophotometer and quantified using a Qubit™ 3.0 Fluorometer with a Qubit™ RNA HS Assay Kit. Assessment of RNA quality was obtained with RIN values >7.0 (RNA Integrity Number) with an Agilent 2100 Bioanalyzer using a Bioanalyzer RNA 6000 Nano assay.

### mRNA Sequencing (mRNA-seq)

mRNA-seq data were generated in Illumina sequencing technology at the Genomics Center of the Laboratory of Animal Biotechnology, ESALQ, University of São Paulo, Piracicaba, Brazil, following the workflow recommended by the instructions of the manufacturer. Polyadenylated cDNA libraries were prepared with 300 μg of RNA depleted from globin mRNAs using the TruSeq^®^ Stranded RNA Sample Preparation Kit. Paired-end sequencing was performed using a HiSeq SBS V4 kit (2 × 100 and 2 × 125 reads) in a HiSeq 2500 sequencer, yielding approximately 71 million reads for each mRNA-seq library.

### RNA-seq Data Analysis

Raw fastq files were checked for quality control using FastQC (16). Illumina sequencing adaptors were trimmed, and low-quality reads (Phred score lower than 20, Q20) were filtered out using Trimmomatic (17). Read mapping was performed with STAR aligner (18) using the human genome reference assembly GRCh38.p38 (provided by The Genome Reference Consortium) annotated by the Ensembl database (19). Concordant uniquely mapped reads were used for downstream analyses. Quantification of reads to gene features used the –quantMode GeneCounts function from STAR. Read counts were used for differential expression analyses with the edgeR package in R (20), applying a quasi-likelihood F test (glmQLFTest function) with batch effect correction. A threshold false discovery rate lower than 0.05 (FDR <0.05) and a cutoff of 2-fold regulation (−1< log2-fold-change >+1) were used to fill the differentially expressed gene (DEG) list for each possible comparison between groups. TPM (transcripts per kilobase million) values were obtained by dividing read counts by the mean length of each gene in kilobases achieved by GTFtools to obtain the reads per kilobase (RPK) (21) and then dividing the RPK values by the sum of all RPK values in millions in a sample. Gene annotations were retrieved from Ensembl using the biomaRt R package (22).

Modular gene coexpression analyses were performed with the CEMiTool R package (23) using embedded functions for gene set enrichment analysis (GSEA) and overrepresentation analysis (ORA) with pathways from the Reactome database (24). The sample heterogeneity of gene expression profiles was assessed by the Molecular Degree of Perturbation (MDP) R package (25). Cell type composition based on blood RNA-seq data was predicted by CIBERSORT (26), a cellular deconvolution method. Long noncoding RNA (lncRNA) gene annotation was performed using the Ensembl BioMart (https://www.ensembl.org/biomart/martview/) (27) and the LNCipedia database (https://lncipedia.org/) (28). The functional genomics public repositories GEO/NCBI (29) and ArrayExpress/EMBL-EBI (30) were used to search other *Leishmania*-related transcriptomes, and three blood transcriptomics studies published elsewhere were selected for comparative analyses of detected differentially expressed long noncoding RNAs (DE lncRNAs): GSE77528 (11), GSE125993 (9) and PRJNA595895 (10).

Prioritized DEGs for lncRNA-mRNA coexpression analysis were obtained by overlapping the DEG lists using a Venn diagram (http://bioinformatics.psb.ugent.be/webtools/Venn/). The proportions of lncRNAs and mRNAs in the DEG results were calculated by the chi-square test using the chisq.test function in R and plotted with the corrplot R package (31). Pearson’s correlation implemented in R base functions was used to find coexpressed pairs of DE lncRNA-mRNA based on prioritized DEG lists encompassed by 147 lncRNAs and 1,263 protein coding genes (PCG, mRNA molecules) that were differentially expressed; only coexpressed pairs with resulting correlation coefficients of −0.8< *r >*0.8 were used for downstream analysis regarding lncRNAs. Network analysis of lncRNA–mRNA pairs was performed using igraph (32) and ggnetwork R packages (33). Hub genes were identified by betweenness and centrality measures. Subcellular localization of lncRNAs was retrieved from the LncSLdb database (34) and/or predicted using the LncLocator webtool (35). Interactions of lncRNAs with other molecules were searched or predicted with RNAInter (36). In the case of interaction with miRNAs, mRNAs targeted by miRNAs were searched using the miRDB database (37).

### Statistical Analysis for the Demographic and Clinical Parameters

Statistical analysis of demographic (**Table 1**) and hematological (**Table 2**) data of the twenty-nine individuals was performed using GraphPad Prism v5.02 software. Each measured parameter was preliminarily assessed for normality using D’Agostino & Pearson and Shapiro–Wilk tests. Student T test was used to compare two groups, if the data follow normal distribution. Mann–Whitney U test was used to compare two groups, if the data followed non-Gaussian distribution. To compare the paired groups, PD0 and PD180, paired T-test and Wilcoxon signed rank test were used for data with normal and non-Gaussian distribution, respectively. Age variance in multiple groups was tested using Kruskall–Wallis followed by Dunn’s post-test for non-Gaussian distribution. *P-*values lower than 0.05 were considered for statistical significance.

**Table 2:** Hematological data of the groups analyzed in this study.

Parameter	PD0—VL diseased (mean ± SD)	PD180—VL cured (mean ± SD)	Asymptomatic (mean ± SD)	Control (mean ± SD)	*p-value* for comparisons*
RBC (10^6^/mm^3^)	3.47 ± 0.35	5.11 ± 0.68	5.16 ± 0.537	4.61 ± 0.37	<0.001 *^b^* (1, 2 and 3)
Hemoglobin (g/dl)	7.98 ± 1.01	12.2 ± 3.6	13.95 ± 2.128	13.23 ± 0.25	<0.01 *^a^* (1, 2 and 3)
Hematocrit (%)	26.55 ± 5.58	40.6 ± 5.4	42.511 ± 4.9	39.85 ± 1.59	<0.01 *^b^* (1, 2 and 3)
Platelets (10^3^/mm^3^)	148.18 ± 81.63	239.9 ± 57.1	277.44 ± 144.53	253.75 ± 73.67	<0.05 *^a^* (1 and 2)
WBC (10^3^/mm^3^)	3,500.1 ± 1,948.8	7,114.5 ± 1,051.2	6,421.1 ± 1,423.7	6,260 ± 1161.2	<0.05 *^a^* (1, 2 and 3)
Neutrophils (10^3^/mm^3^)	1,329.5 ± 1,318.7	3,224.9 ± 753.3	3,286.7 ± 423.5	3,422.5 ± 258.505	<0.01 *^b^* (1, 2 and 3)
Eosinophils (10^3^/mm^3^)	51.00 ± 123.84	676.9 ± 531.2	332.8 ± 175.98	213.2 ± 144	<0.01 *^b^* (1 and 2)
Basophils (10^3^/mm^3^)	10.10 ± 21.76	98.8 ± 100.2	84.8 ± 43.4	87 ± 40.4	<0.001 *^b^* (1, 2 and 3)
Lymphocytes (10^3^/mm^3^)	1,644.70 ± 861.50	2,643.4 ± 956.2	2,273.9 ± 1,256.8	2,065 ± 626.5	<0.01 *^a^* (1)
Monocytes (10^3^/mm^3^)	360.20 ± 186.72	470.5 ± 186.6	445.2 ± 215.09	473.5 ± 141.7	NS *^a^*

*Comparisons: 1 = PD180 vs PD0; 2 = Asymptomatic (A) vs PD0; 3 = Control (C) vs PD0; 4 = A vs PD180; 5 = A vs C; 6 = C vs PD180.

a p-value calculated by t-Test (paired t-test for PD180 vs PD0).

b p-value calculated by Mann–Whitney test (Wilcoxon signed rank test for PD180 vs PD0).

NS, non-significant.

## Results and Discussion

### Features of the Polyadenylated Transcriptome of Blood From Visceral Leishmaniasis Patients

We generated and analyzed bulk RNA-seq data of whole blood samples collected from 11 patients with active VL (“P–D0” abbreviations) and six months after the treatment, when these individuals were considered clinically cured (“P–D180” abbreviations). To gather insights into not only the immunopathophysiology of the disease but also the molecular mechanisms involved in *L. infantum* infection, we enrolled nine asymptomatic individuals (positive for anti-*L. infantum*, “A” abbreviations) and nine healthy subjects as control individuals (“C” abbreviations). Our analyses consisted of a total of 40 RNA-seq libraries depleted from globin and poly(A)+ selected mRNA, yielding an average of 4 Gb and 24 million paired-end mapped reads per library. The main characteristics of the analyzed groups and the clinical laboratory data for VL patients are displayed in **Tables 1** and **2**, respectively.

**Figure 2:**
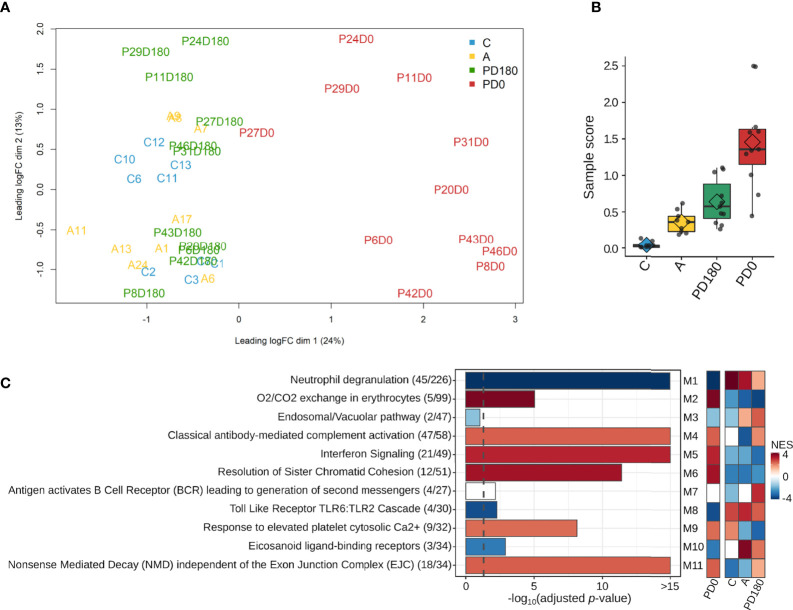
Overview of the blood transcriptomes of this study. A total of 14,247 genes were expressed across the four groups, which encompassed 40 samples from 29 subjects. **(A)** Multidimensional scaling (MDS) plot and **(B)** molecular degree of perturbation (MDP) plot of all expressed genes in the four groups; **(C)** most significantly enriched pathways retrieved from the Reactome database for each module. The gene ratio for each pathway is displayed in parentheses. Colors in the Reactome enrichment graph refer to module activity in VL patients (PD0 group) as represented by the GSEA plot (heatmap in the right panel) displaying the module activity for each group. Color intensity is proportional to NES (normalized enrichment score). The graded scale side bar (NES) from red to blue indicates higher and lower activity, respectively, based on the ranked expression level.

After filtering out genes with low expression (cpm <2; N <3) from the dataset, a total of 14,247 genes remained to be further analyzed. A multidimensional scaling (MDS) plot of the gene dataset was built to visualize the similarity across the 29 individuals represented by 40 samples. As shown in **Figure 2A**, there was clear segregation between active VL (PD0 group) and VL-free (PD180, A and C groups) individuals. Apart from P27 patient, all VL patients presented noticeably distinct gene expression patterns when considered clinically cured of the disease (PD180 group), which clustered together with other VL-free groups, asymptomatic (A) and healthy uninfected controls (C). Despite being within the same cluster, patients with active VL presented dissimilarities among them, reflecting the multifaceted nature of the disease. The heterogeneity of these blood transcriptomes was also assessed by molecular degree of perturbation (MDP) scores (25), in which PD0 samples presented the highest scores as expected, but it is also interesting to note that among VL-free groups, asymptomatic and cured patients presented distinct scores from healthy uninfected people (**Figure 2B**).

For an overview of the system-level functionality of all genes expressed in blood during the development of visceral leishmaniasis, we performed modular gene coexpression analysis using CEMiTool (23). Eleven different coexpressed modules were identified in the Gene Set Enrichment Analysis (GSEA), out of which 10 presented at least four significantly enriched pathways in the Over Representation Analysis (ORA). In the GSEA plot (**Figure 2C**, right panel), the activity of each module is displayed for all studied groups. Among the modules enriched across all groups, we depicted the modules M2, M5 and M6 with high Normalized Enrichment Scores (NES) that were only active in the VL group (PD0), which presented enriched pathways related mainly to “O2/CO2 exchange in erythrocytes”, “Interferon Signaling” and “Cell Cycle Checkpoints”, respectively. The crucial role of IFN- γ signaling in leishmaniasis (38) and the key roles of type I interferons in protozoan infections, including *Leishmania*, have been increasingly established in recent years (39). Modules M1 and M8 were related mainly to the pathways “neutrophil degranulation” and “Toll-like receptor cascades”, respectively, and presented a low NES (activity) in active VL (**Figure 2C**, right panel). Neutrophils are massively recruited upon *Leishmania* infections, but the parasite efficiently evades neutrophil killing (40). They also influence the different forms of leishmaniasis, but they have been reported to play either protective or harmful roles during infection, depending on *Leishmania*-infecting species (41). The neutrophil response to infection outcomes seems to depend on their recruitment phase and tissue environment (42). Toll-like receptors (TLRs) are another innate immune branch acting in *Leishmania* infections, with multiple TLRs being activated simultaneously, and the interplay among them may influence the final outcome of infection (43). In an experimental model of *L. infantum* infection, TLR2 was shown to be important in promoting a protective immune response and effector mechanism of neutrophils (44). In addition, the TLR4 and IFN-I pathways play significant roles in preventing chronic inflammatory processes and immunopathology during *L. infantum* infection (45). All significantly enriched pathways elicited in VL are visualized in **Presentation 1** within each module.

**Figure 3:**
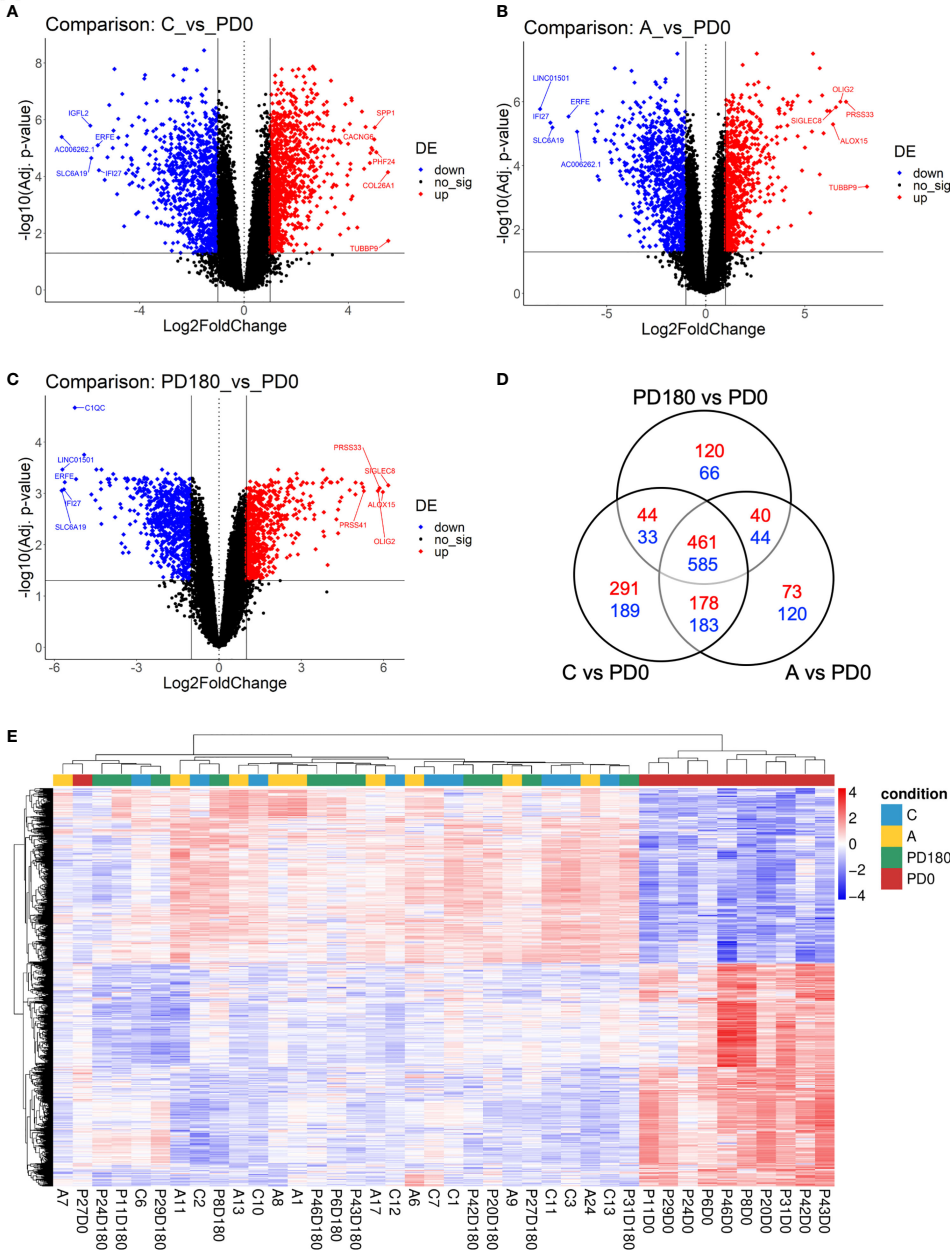
Differential expression analysis comparing VL patients to nondiseased patients identified hundreds of differentially expressed genes (DEGs). Volcano plots highlighting genes significantly regulated (FDR <0.05, horizontal threshold line set on y-axis) in the comparisons C vs. PD0 **(A)**, A vs. PD0 **(B)** and PD0 vs. PD180 **(C)**. Vertical lines at -1 and +1 on the x-axis indicate the expression level criteria of fold decrease or increase, respectively, applied to DEGs that were further analyzed (all genes colored in blue or red); **(D)** Venn diagram displaying the number of exclusive DEGs for each comparison, as well as the number of shared DEGs among them. Dashed squares indicate the DEG lists used as subsets of prioritized genes in the mRNA-lncRNA coexpression profile analysis; **(E)** Heatmap of 1,045 DEGs shared among the three comparisons (suggested to be the gene signature of VL disease status), depicting the clustering of samples in two major groups: VL patients (PD0 labels, in red), the very consistent cluster on the right side and a heterogeneous cluster encompassing the nondiseased groups (**C, A** and PD180 labels, in blue, yellow and green, respectively) split into minor clusters. Z scores of cpm read counts were used, and a graded color scale from red to blue indicates whether the level of gene expression was above or below the mean (i.e., up- or downregulation).

**Figure 4:**
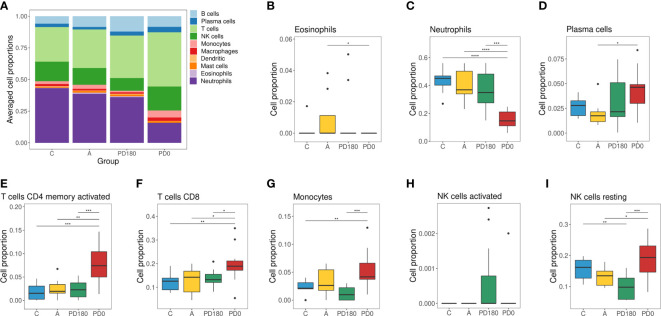
Cellular deconvolution of blood transcriptomes in human visceral leishmaniasis using the CIBERSORT method: **(A)** Leukocyte proportions inferred from gene expression profiles of blood samples. Plots by cell type displaying the relative cell proportions for eosinophils **(B)**, neutrophils **(C)**, plasma cells **(D)**, activated memory CD4 cells **(E)**, CD8 T cells **(F)**, monocytes **(G)**, activated NK **(H)** cells and resting NK cells **(I)**. Groups were classified as active VL (PD0) and VL-free (PD180, A and C groups), in which the PD180 subjects were treated and considered clinically cured after 180 days of disease follow-up (PD0 and PD180 are paired groups, n = 11); A: asymptomatic (n = 9); C: healthy uninfected controls (n = 9). The differences between cell proportions were evaluated by Wilcoxon with Holm’s correction. *P < 0.05, **P < 0.01, and ***P < 0.001.

Next, we proceeded to differential expression analysis to identify genes (DEGs) that were significantly regulated. The lists of DEGs (cutoff: FDR <0.05 and log2-fold-change ± 1) for pairwise comparisons can be found in **Supplementary Table 1**. As observed by others (9, 11), no DEG was found between asymptomatic and healthy control individuals considering the *post hoc* test at FDR<0.05. Considering that both groups basically include healthy and VL-free individuals, it is coherent do not finding difference statically significant between them. We focused on comparisons of the three non-diseased groups, A, C and PD180, against the active VL group, PD0. The comparisons presented an average of 1,680 DEGs each, with many hundreds of up- or downregulated genes (**Figures 3A–C**). All three comparisons presented some dozens of highly regulated genes (−3< log2-fold-change >3) with very statistically significant FDR values (FDR <0.0001), as can be observed at the superior corners of the volcano plots. Among many DEGs, we highlighted PRSS33 (serine protease 33), which was upregulated in the nondiseased groups (i.e., downregulated during active VL) at least 32-fold. PRSS33, also known as EOS, was primarily identified as expressed predominantly by macrophages, and also in peripheral leukocytes, and was detected in many organs, such as spleen, intestine, lung and brain (46). More recently, it was found that the production of PRSS33 by leukocytes is attributed specifically to eosinophils, which present constitutive expression at the mRNA level and cell surface expression at the protein level rather than being secreted (47). Interestingly, the gene expression pattern of PRSS33 along with IL10, SLFN14, and HRH4 was identified as a transcriptional signature to assess the treatment efficacy of visceral leishmaniasis in HIV patients (10). Here, the eosinophil count in VL patients was significantly decreased during *L. infantum* infection (**Table 2**); interestingly, the eosinophil gene signature was only detected in asymptomatic individuals (**Figure 4B**). The important role of eosinophils during *Leishmania* infection has become increasingly evident since many studies have demonstrated that eosinophils are able to control parasite load and interact with innate and adaptive immune responses, mainly by shaping macrophage responses (48–50). Another interesting DEG is interferon alpha inducible protein 27 (IFI27), which was highly downregulated in the non-diseased groups (i.e., upregulated during active VL) by an average of 64-fold. IFI27 (also known as ISG12) is a gene highly induced by type 1 interferon with pro-apoptotic effects (51) and antiviral activity (52, 53) and a potential biomarker identified through transcriptomics in some cancers, such as pancreatic adenocarcinoma (54).

A Venn diagram of these three comparisons (**Figure 3D**) displayed the number of shared or exclusive DEGs. Considering all intersections, when the redundancy was filtered out, we identified 2,427 unique DEGs. The central overlap presented 461 upregulated and 584 downregulated genes, which represents the “disease” gene set, because regardless of the non-disease group, these genes were significantly regulated in the disease group. An unsupervised clustering of this disease status gene signature (1,045 DEGs) showed the substantial grouping of PD0 patient samples (**Figure 3E**). In addition, we depicted the exclusive gene sets of PD180 vs. PD0 and A vs. PD0 comparisons, and the intersection between them, which accounted for 40 up- and 44 downregulated genes and may reveal genes related to a “molecular footprint” of the *L. infantum* infection because both cured patients and asymptomatic individuals had already been infected (unrelated to whether they became sick or not). The exclusive gene set of PD180 vs. PD0 may indicate genes associated with “molecular scarring” triggered by immunopathological mechanisms of the disease. Last, the exclusive gene set of A vs. PD0 comparison (73 up- and 120 downregulated genes) might uncover genes related to infection control and resistance mechanisms, which abrogate the development of visceral leishmaniasis. To assign these signatures regarding “molecular footprint”, “molecular scarring” and “controlling of infection” is difficult due to the individual sample heterogeneity in non-diseased groups (as observed in **Figure 2B** and **Supplementary Figure 1** for individual samples) but is still a valid assumption since the groups PD180 (cured) and Asymptomatic (A) groups presented higher molecular perturbation scores than the healthy control group, as assessed by the MDP tool (**Supplementary Figure 1**).

Furthermore, to assess the composition of cellular subtypes of leukocytes in VL, we applied the CIBERSORT method for deconvolution analysis of blood transcriptomes (**Figure 4**). In addition to standard subtypes observed in blood count data (**Table 2**), the leukocyte gene signature matrix was able to discriminate natural killer (NK) cells, and subsets of T and B cells. Through data reuse, we also compared our cellular profile with the composition of another study in VL with the blood transcriptome obtained by microarray (11) (**Supplementary Figure 2**). The cell proportions in whole blood RNA-seq data of VL patients (PD0) showed variations mainly in neutrophils, macrophages, monocytes, NK cells, T lymphocytes and plasma cells (**Figure 4A**). When compared to the study of Gardinassi et al. (**Supplementary Figure 2**), in general, similarities in alteration tendency between both transcriptomes regarding cellular composition but also unmatched alterations for some cell subtypes were observed. In addition to the composition, the proportions of most cell types (irrespective of which group) were different between the two datasets, such as neutrophils, plasma cells (both higher in this study) and monocytes (lower in this study). The profiles for neutrophils, plasma cells, activated memory CD4 T cells and CD8 T cells presented similar tendencies in both studies (**Figures 4C–F**), whereas the profile for monocytes presented the opposite behavior; here, it was increased in VL, but in Gardinassi et al., diseased patients presented a reduction in monocytes (**Figure 4G**). However, the variation in the number of monocytes before (PD0) and after treatment (PD180) was not significant, as shown in **Table 2**. This increase checked through CIBERSORT is related to the gene expression profile signed by monocytes. For NK cells, the profile presented complementary findings for activated (**Figure 4H**) and resting (**Figure 4I**) states between both studies, but both studies presented higher proportions of NK cells in active VL than in posttreatment, asymptomatic and control groups (**Figure 4A**). NK cells are able to recognize and are activated by *Leishmania* lipophosphoglycan (LPG), which is a dominant promastigote-specific surface glycoconjugate (55), *via* TLR-2 (56), but recently, it has been reported that human NK cells cannot be straightforwardly activated by *Leishmania* promastigotes and require monocyte-derived signals, such as transpresentation of IL-18, for their activation (57).

### Long Noncoding RNA (lncRNA) Expression in Blood Upon *L. infantum* Infection

**Figure 5:**
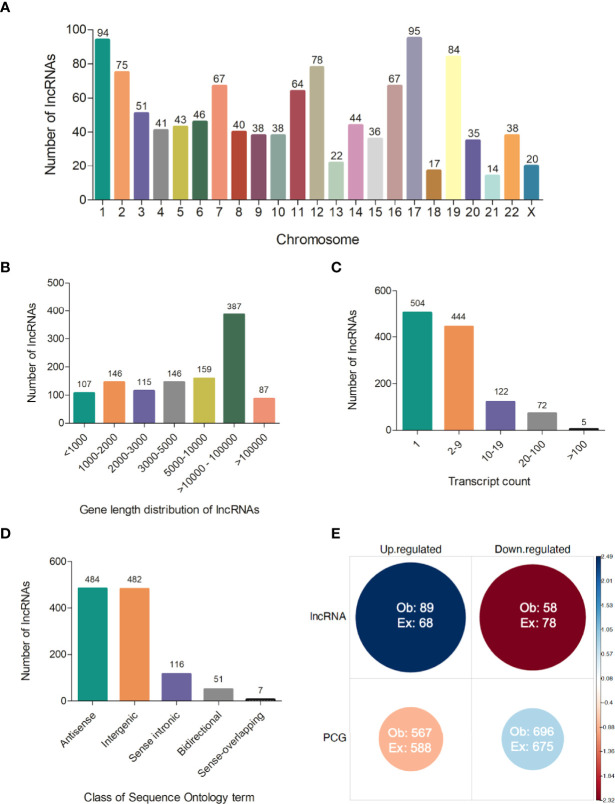
Features of lncRNAs detected in polyadenylated RNA-seq of human visceral leishmaniasis. **(A)** Chromosomal distribution of lncRNAs across the 22 autosomes and the X chromosome; **(B)** Gene length distribution of lncRNAs; **(C)** Number of transcripts presented by each lncRNA; **(D)** Classification of lncRNAs according to sequence ontology terms; **(E)** Pearson’s chi-square test using the number of lncRNAs and protein-coding genes (PCGs) and regulation patterns (up- or downregulated transcripts) in DEG datasets (comparisons of non-diseased groups, A, C and PD180 to PD0 active VL). The size and color intensity of circles are proportional to the contribution of the cell to the significance of the chi-square test. The standardized residuals are scaled at the sidebar, where positive and negative values indicate positive and negative associations, respectively. Ob, observed value; Ex, expected value, χ^2^ = 12.343, df = 1, *p-value* = 0.0004.

Several processed lncRNAs are capped and polyadenylated (15). Due to this feature, poly-A selection as a strategy of enrichment used in our RNA-seq was able to reveal the set of lncRNAs expressed in the blood of visceral leishmaniasis patients. Therefore, we also addressed the analysis of long noncoding RNAs, which is completely new in the VL transcriptomics field. From the total of 14,247 expressed genes, we identified 1,147 transcripts annotated as lncRNAs, according to gene biotype annotations in BioMart Ensembl. They were widely distributed across human chromosomes, from 1 to 22 and X, in which chromosomes 17 and 1 accounted for the largest numbers of lncRNAs, 95 (~8.3%) and 94 (~8.2%), respectively (**Figure 5A** and **Supplementary Table 2**). No lncRNA from the Y chromosome was detected in our RNA-seq dataset. The average gene length of the identified lncRNAs was 311,117 bp, with 9.3% being shorter than 1,000 bp and 41% being higher than 10,000 bp (**Figure 5B** and **Supplementary Table 2**). According to our analyses, 504 (~44%) lncRNAs were found to be expressed as unique transcripts (one transcript count in annotated genome version). Approximately 28% of lncRNAs had a transcript count from 2 to 5 (28%), and one expressed lncRNA (ENSG00000179818) presented 239 transcripts (**Figure 5C** and **Supplementary Table 2**). We identified the class of sequence ontology terms of 1,140 from 1,147 lncRNAs, namely, 484 (~42%) antisense, 482 (~42%) intergenic, 116 (~10%) sense intronic, 51 (~4.5%) bidirectional, and 7 (~0.6%) sense-overlapping lncRNAs (**Figure 5D** and **Supplementary Table 2**).

Based on the 2,427 unique DEGs found in the comparisons of nondiseased groups (A, C, and PD180) versus active VL, we searched for lncRNA gene biotype annotations in specific gene sets presented earlier (1,512 DEGs), focused on the central intersection (1,045 common DEGs) and the intersections between PD180 vs. PD0 and A vs. PD0 comparisons (88 DEGs), and their exclusive gene sets, which account for 186 and 193 DEGs, respectively (**Figure 3B**). A total of 147 lncRNAs (9.7%) out of 1,512 DEGs were found to be differentially expressed in this dataset (**Supplementary Table 3**), in which 89 (60.5%) and 58 lncRNAs were up- and downregulated, respectively, in the non-diseased groups compared to PD0 patients with active VL. Of note, the PD180 vs. PD0 exclusive gene set, with 186 DEGs, included 35 lncRNAs (19%), among which the expression of 30 genes significantly increased upon the clinical cure of VL, which might suggest that the suppression of these lncRNAs is related to the transcriptional regulation of protein-coding genes (PCGs) involved in the immunopathology of disease.

Notably, we highlighted MALAT1 (metastasis-associated lung adenocarcinoma transcript 1), which presented a statistically significant 2-fold increase in cured VL patients (PD180 group, **Supplementary Table 1**). MALAT1 is one of the most studied lncRNAs, exhibiting a variety of molecular regulatory functions in transcription and alternative splicing by binding in chromatin regions and binding in a plethora of protein, miRNA and mRNA molecules (58). MALAT1 has been extensively studied not only in oncology but also in many inflammatory diseases, where it plays a controversial role due to its action as either an oncogene or a tumor suppressor gene depending on the type of cancer (59). Interestingly, this controversial role of MALAT1 was also observed in parasitic protozoan infections, where deficiency of this lncRNA was important to enhance immunity and for clearance of *L. donovani* in a VL mouse model; however, in an experimental malaria model, *Malat1^−/−^* mice presented more severe disease (60). In this latter cited work, the authors suggest that MALAT1 is a nonredundant regulator of immunity by promoting the expression of the Maf/IL-10 axis in effector CD4^+^ T cells. This immune regulator function of MALAT1 was also found in tolerized mice with cardiac allografts by inducing tolerogenic dendritic cells and regulatory T cells through the miRNA-155/DC-SIGN/IL10 axis (61).

**Figure 6:**
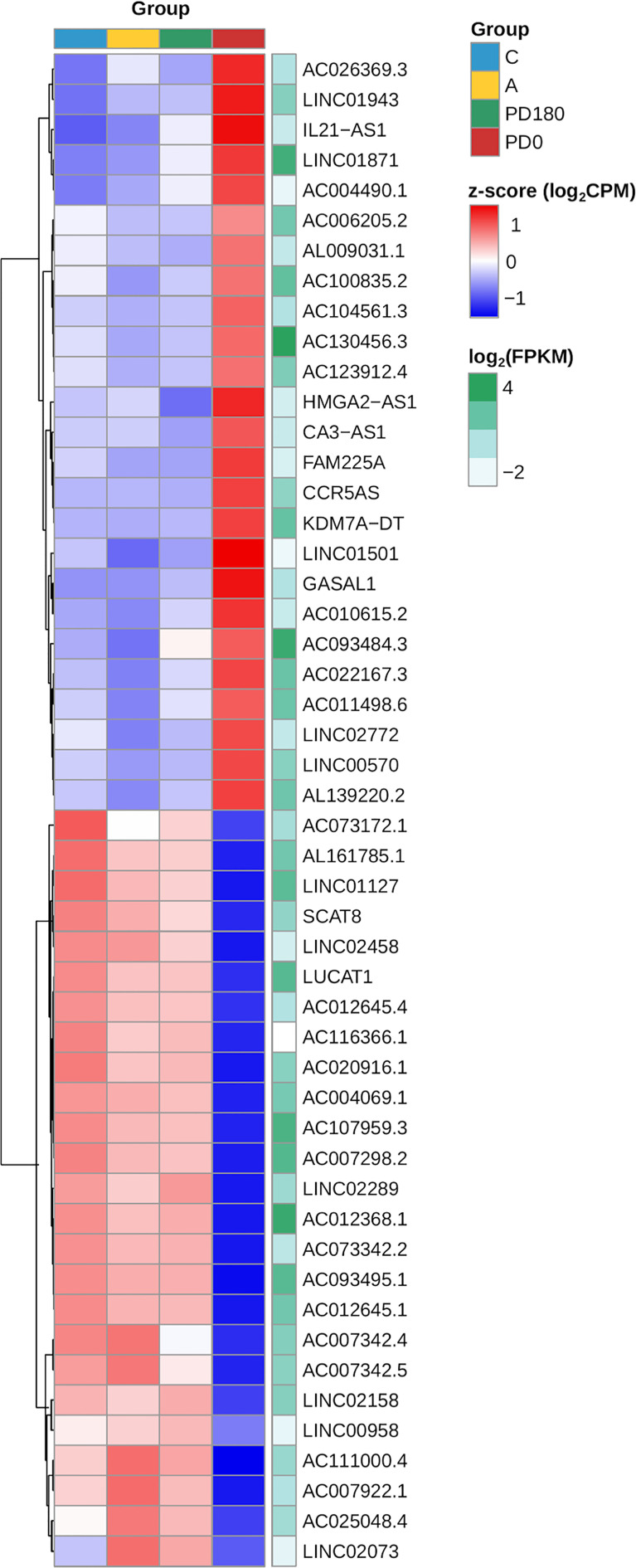
Heatmap of the expression profiles of the top 20 lncRNAs expressed in VL patients (PD0). The top selection retrieved those genes within the dataset of 147 primarily selected DE lncRNAs (**Supplementary Table 2**) based on ranking of statistical significance (FDR values) followed by fold regulation (log_2_FC values). Gene expression regulation by group is represented by the z score of the cpm read count, and the transition color scale from red to blue indicates up- and downregulation, respectively. Gene expression level is represented by mean FPKM values at the side (green heatmap column), where color intensity toward dark green indicates increasing levels (highly expressed lncRNAs).

Although lncRNAs have portrayed less than a tenth of the polyadenylated transcriptome, the proportion of lncRNAs in the DEG gene set and their regulation pattern (up- or downregulation) were significantly associated and enriched in the differential expression data as calculated by the chi-square test (p-value = 0.0004, **Figure 5E**). The clustering of expression data of the 147 differentially expressed lncRNAs highlights the distinct expression pattern of selected lncRNAs in the active VL group (PD0, **Figure 6**). Generally, lncRNAs are expressed at low levels (62). Notably, some lncRNAs presented relatively high levels of expression, such as LINC01871 and AC012368.1 (**Figure 6**, green heatmap), as indicated by their TPM values. Other lncRNAs presented marked fold change regulation, such as IL21-AS1 and AC111000.4, which presented an average 16-fold increase (log_2_FC = 4) and an average 32-fold decrease (log_2_FC = −5), respectively, in the PD0 active VL group compared to the non-diseased groups (**Supplementary Table 3**). The most upregulated DE lncRNA in VL patients was the completely unknown lncRNA LINC01501 (long intergenic nonprotein coding RNA 1501), which presented an average fold increase of 64× (log_2_FC = 6) in the PD0 group (**Supplementary Table 4**).

### lncRNA–mRNA Coexpression Analysis

**Figure 7:**
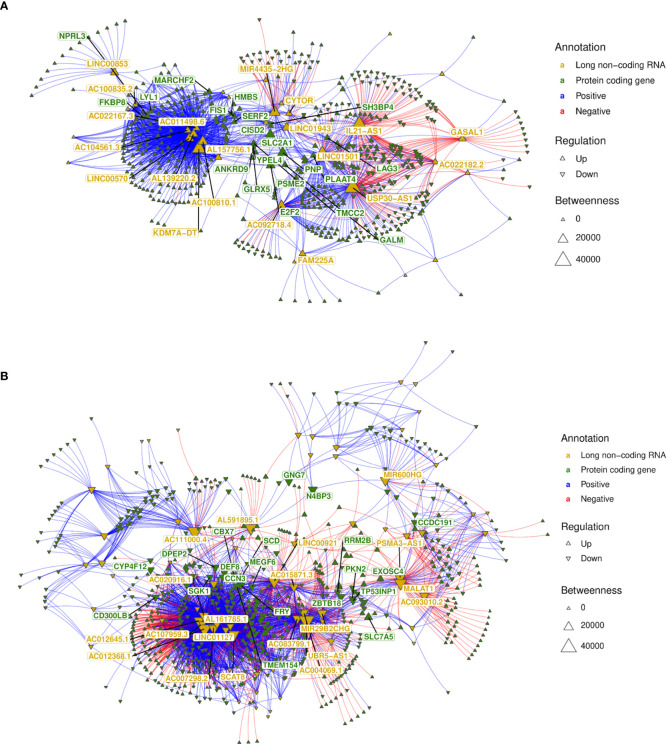
lncRNA–mRNA coexpression network analysis. **(A)** A network was built with all highly correlated lncRNA–mRNA pairs (1,870 pairs; **Supplementary Table 6**) obtained for 58 lncRNAs downregulated in non-diseased groups (i.e., upregulated in VL patients—PD0); **(B)** A network was built with all highly correlated lncRNA–mRNA pairs (4,256 pairs; **Supplementary Table 6**) obtained for 89 lncRNAs upregulated in nondiseased groups (i.e., downregulated in VL patients—PD0). The top 20 hubs for lncRNAs and mRNAs are flagged with gene symbols in yellow and green, respectively.

Subsequently, we integrated the profiles of protein-coding genes (i.e., mRNAs) and long noncoding RNAs by performing lncRNA–mRNA coexpression analysis. Pearson correlations for the lncRNA–mRNA coexpression profile encompassing the 147 lncRNAs (89 up- and 58 downregulated) and the 1,263 (567 up- and 696 downregulated) mRNAs differentially expressed (as numbered in **Figure 5D**) identified 4,901 positive and 1,223 negative highly correlated pairs of lncRNA–mRNA associations (**Supplementary Table 5**). Networks were built using these coexpression correlations (**Figure 7**) to identify hubs of lncRNAs (e.g., KDM7A-DT, USP30-AS1 and LINC01501) and mRNAs (e.g., NPRL3, LAG3 and E2F2). The top 20 hubs within these networks for lncRNAs and mRNAs were identified by flagging them with their respective gene symbols. The top pairs of coexpressed lncRNA–mRNA positively (e.g., AC111000.4-CCR3; AC111000.4-IL5RA) and negatively correlated profiles (e.g., USP30-AS1-CCN3; LINC01501-IL1RAP) were identified by ranking Pearson’s correlation results (**Supplementary Table 5**). The main pathways enriched by this highly correlated lncRNA–mRNA expression profile corroborated the results found when the whole expression profiling was analyzed (**Figures 2C, D** and **Presentation 1**), with common Reactome enrichment results (**Supplementary Table 5**).

**Figure 8:**
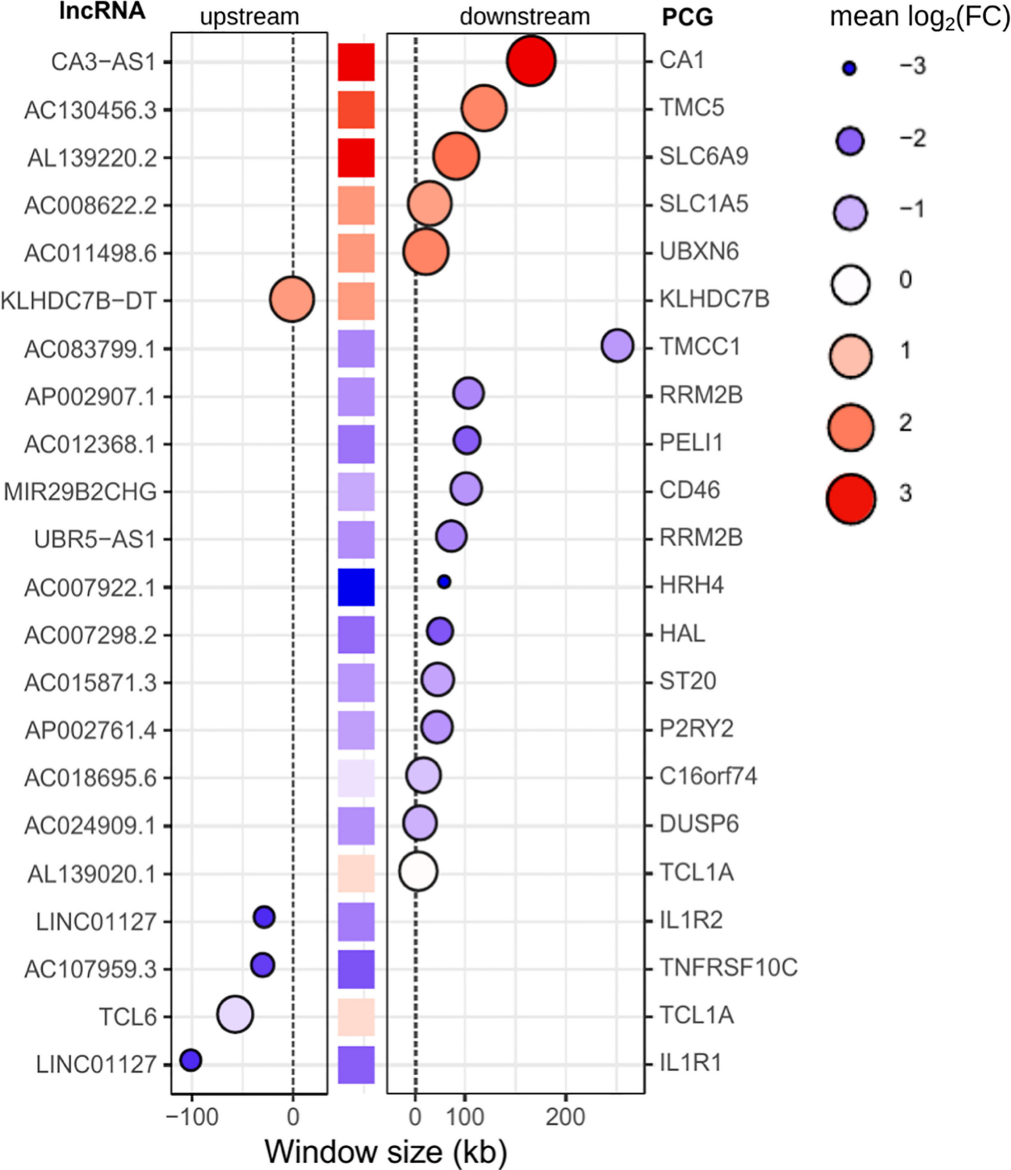
Potential *cis-acting* lncRNAs inferred from highly correlated lncRNA-mRNA pairs in the genomic vicinity. A window size of 300 kb was set to consider *cis*-regulation. Gene IDs on the left refer to lncRNAs, whereas gene IDs on the right refer to PCGs (mRNAs). The central column of squares indicates the position of PCGs related to lncRNAs, and the graded color indicates the pattern regulation in PD0 (logFC). Bubbles indicate the position of lncRNAs upstream (left panel) or downstream (right panel) of the PCG. The size and color intensity of bubbles indicate the pattern regulation of lncRNAs in PD0. The blue scale indicates downregulation, whereas the red scale indicates upregulation in VL patients (PD0).

Regulatory networks of lncRNAs act in *cis-* and *trans-*regulation. Human genome annotation has revealed that the vicinity of PCGs is surrounded by lncRNAs, and as we verified by sequence ontology of expressed lncRNAs, most of them were classified as antisense or intergenic (**Figure 5D**). *Cis*-acting lncRNAs play gene regulation functions from their own transcription sites, operating in PCGs at proximal distances within the same chromosome (63). Many intergenic (lincRNA) long noncoding RNAs are placed in topologically associated domains (TADs), an approximately 1 Mb genomic segment featuring chromatin interactions; *cis*-acting lincRNAs have been associated with modeling the chromosomal architecture (64). To infer potential *cis-*regulation, the highly correlated lncRNA–mRNA pairs were tracked into their genomic coordinates. lncRNAs within a 300-kb window size from the transcription start site (TSS) of the correlated PCG were retrieved, resulting in 22 potential *cis-*acting lncRNAs all positively associated with their respective mRNAs (**Figure 8**). The majority are located downstream of the TSS of the PCG pair, and the five sites upstream of the PCG pair are lincRNAs. Out of six potential *cis*-acting lncRNAs upregulated in VL patients, CA3-AS1 (CA3 antisense RNA 1) presented higher fold regulation than the non-diseased group (log_2_FC = 3.035, approximately 9-fold increase). CA3-AS1 has been identified as a key lncRNA in gastrointestinal cancers (65–67). Even more differentially expressed, its correlated PCG pair, CA1 (carbonic anhydrase 1), presented a 24-fold increase during active *L. infantum* infection. GO (Gene Ontology) biological process classification for CA1 revealed that this protein participates in the interleukin-12-mediated signaling pathway (GO:0035722) and is involved in gene and protein expression by JAK-STAT signaling after IL-12 stimulation according to the Reactome database (R-has-8950505).

As shown by genomic localization mapping, most coexpressed lncRNA–mRNA pairs were located in different chromosomes or distally in the same chromosome, making the majority of lncRNA networks hypothetically play a role in transcription as a *trans*-acting regulation. In fact, lncRNAs that may act near their transcription sites may also undertake regulatory functions far from the TSS or even outside the nucleus (12, 63). Mechanisms for the transregulation of transcription depend on interactions of lncRNAs with proteins, DNA and other RNA molecules. Additionally, depending on their subcellular localization, lncRNAs may interfere with posttranscriptional and intracellular signaling (15). Based on this framework, we proceeded to mine data for lncRNA subcellular localization and lncRNA interactions using the LncSLdb database (34) and the LncLocator webtool (35) for subcellular localization and the RNAInter database (36) for an interaction overview of hub lncRNAs selected from coexpression network analyses (**Figure 7** and **Supplementary Table 5**), and inferred *cis*-acting lncRNAs (**Figure 8**). The analysis of this subset comprises 51 lncRNAs from the 147 DE lncRNA list (**Supplementary Table 4**) detailed in our work. A piece of this interactome is summarized in **Table 3**, and interactions related to the 51 lncRNAs are available as additional material (**Supplementary Table 6**).

**Table 3:** Potential lncRNA subcellular localization and interactors based on highly correlated lncRNA–mRNA pairs differentially expressed during *L. infantum* infection.

Potential *cis*-acting					
lncRNA	PCG (mRNA) pair in chromosome vicinity	Number of PCGs coexpressed (total; positive; negative) *^b^*	Subcellular localization *^c^*	Number and type of interaction*^d,e^*	Coexpressed PCG pairs *^f^*
↑ AL139220.2 *^a^*	↑ SLC6A9	128; 128; 0	Cytosol^c2^	4 miRNA: hsa-miR-107, hsa-miR-103a-3p, hsa-let-7c-5p, hsa-let-7b-5p	miRNA targets: CARM1, ANK1, IFIT1B, TRIM10, PSMF1, FRMD4A, TAL1, TSPAN5, SERF2, XK, CDC34, RBM38, BCL2L1, PBX1, IGF2BP2
				5 TF	LYL1, KLF1, PBX1, MXI1, TAL1
↑ CA3-AS1	↑ CA1	22; 22; 0	–	1 miRNA: hsa-miR-93-5p	miRNA targets: NEDD4 L, MXI1
				1 TF	MXI1
↑ AC130456.3	↑ TMC5	38; 38; 0	–	2 TF	LYL1, TAL1
↓ UBR5-AS1	↓ RRM2B	166; 100; 66	–	5 TF	↓ MXD1, ↓ BACH1, ↑ E2F2, ↑ FOXM1, ↑ EZH2
↓ AC012368.1	↓ PELI1	269; 172; 97	–	5 TF and 1 histone	↓ MXD1, ↓ FOS, ↑ E2F8, ↑ E2F7, ↑ EZH2, ↑ CENPA (histone)
↓ LINC01127	↓ IL1R1	214; 171; 43	–	4 TF	↓ MXD1, ↓ FOS, ↑ E2F7, ↑ EZH2
	↓ IL1R2			1 DNA	↑ NUF2
↓ AC107959.3	↓ TNFRSF10C	214; 160; 54	–	5 TF and 1 histone	↓ MXD1, ↓ FOS, ↑ E2F8, ↑ E2F7, ↑ EZH2, ↑ CENPA (histone)
**Potential *trans*-acting**					
**lncRNA**	**Top coexpressed PCG pairs *^g^***	**Number of PCGs coexpressed (total; positive; negative) *^b^***	**Subcellular localization *^c^***	**Number and type of interaction*^d,e^***	**Coexpressed PCG pairs *^f^***
↑ FAM225A	↑ C2	16; 16; 0	Nucleus/Cytoplasm^c1^ Cytosol^c2^	1 miRNA: hsa-miR-1-3p	miRNA target: UBE2L6
	↑ FBXO6				
	↑ PSME2				
↑ KDM7A-DT	↑ UBB	154; 152; 2	–	5 TF	↑ LYL1, ↑ KLF1, ↑ PBX1, ↑ MXI1, ↑ TAL1
	↑ STMP1				
	↑ RBM38				
↑ IL21-AS1	↑ FABP5	117; 47; 70	Cytoplasm^c2^	2 TF	↓ TLE3, ↑ EZH2
	↑ CDKN2A				
	↑ CTLA4				
↑ AC092718.4	↑ CDCA3	87; 78; 9	–	4 TF and 1 histone	↑ E2F2, ↑ FOXM1, ↑ MYBL2, ↑ EZH2, CENPA (histone)
	↑ NCAPH				
	↓ IRS2				
↑ MIR4435-2HG	↑ H2AJ,	49; 33; 16	–	6 miRNAs: hsa-miR-6754-5p, hsa-miR-128-3p, hsa-miR-1185-5p, hsa-miR-105-5p, hsa-miR-103b, hsa-miR-1-3p,	miRNA targets: ↓ ZFP28, ↓ PDE3B, ↓ ATP2B1, ↓ HDAC9, ↓ EPHA4, ↓ MOB3B, ↓ ZNF677, ↓ TCTN1, ↓ MAP3K1, ↑ POMP, ↑ E2F2, ↑ EMC3
	↑ PTMS,				
	↓ HDAC9				
				1 TF	↑ BATF
↑ GASAL1	↓ EEPD1,	38; 5; 33	Cytosol^c2^	7 miRNAs: hsa-miR-93-5p, hsa-miR-519d-3p, hsa-miR-20b-5p, hsa-miR-20a-5p, hsa-miR-17-5p, hsa-miR-106b-5p, hsa-miR-106a-5p	miRNA targets: ↓ TMCC3, ↓ SORL1 e ↓ OLIG1
	↓ ADGRE3,				
	↑ IFNG				
↓ AC004069.1	↑ DNAJA4,	65; 53; 12	Cytosol^c1^, Ribosome ^c1^, Nucleus^c1^ Cytoplasm^c2^	2 miRNAs: hsa-miR-10b-5p, hsa-miR-10a-5p	miRNA targets: ↓ BAZ2B, ↑ NEDD4 L, ↑ ARHGEF12
	↓ ST3GAL6				
				1 TF	↓ MXD1
↓ PSMA3-AS1	↓ ZNF439	44; 19; 25	–	4 miRNAs: hsa-miR-106b-5p, hsa-miR-106a-5p, hsa-miR-101-3p, hsa-miR-105-5p	miRNA targets: ↓ ZBTB18, ↓ ZFP28, ↓ PKN2, ↓ TP53INP1, ↓ ZFYVE16, ↓ VCPKMT, ↓ TMEM65, ↓ ZNF677, ↓ TLR10, ↑ TGFB1I1, ↑ PBX1
	↑ PSMF1				
				2 TF	↑ LYL1, ↑ PBX1

a lncRNA hub in network analysis (**Figure 7**).

b Numbers were extracted from the expression correlation results available in **Supplementary Table 4**.

c Highlighted results from data mining in the LncSLdb Database (c1) or prediction by the LncLocator tool (c2). For in silico prediction, only scores higher than 0.7 (ranging from 0 to 1) were considered.

d Highlighted results from data mining in RNAInter Database. Interactions of lncRNAs with microRNAs (miRNAs), transcription factors (TFs), RNA binding proteins (RBPs), DNA and histone modifications; when lncRNAs exhibited interactions with miRNAs, targeted PCGs were listed.

e All lncRNAs displayed here present at least 35 histone modification results.

f PCGs were extracted from the expression correlation results available in **Supplementary Table 4**.

g PCGs with the highest correlation coefficients according to the results available in **Supplementary Table 4**. Up or down arrows indicate the expression regulation pattern in VL patients (PD0 group).

Most of the interactors found in RNAInter is a database belong to microRNA (miRNA) and transcription factor categories. Of note, 8 out of 51 lncRNAs did not present any annotation in noncoding RNA databases (**Supplementary Table 6**) but reached high scores for cytoplasm/cytosol subcellular localization prediction. Three lncRNAs of these 8 (AL157756.1, AC100810.1, and AC007922.1) presented exclusively at least 40 highly positively coexpressed PCGs (i.e., same pattern regulation) and three other lncRNAs presented mixed correlations of PCG pairs, including AC111000.4, the most downregulated lncRNA in VL patients. For those interactions with miRNA, we searched for miRNA targets using miRDB (37) to provide the PCGs coexpressed with the respective lncRNAs, which in turn were retrieved from coexpression network analysis (last column of **Table 3**). lncRNAs that present miRNA binding sites can interact with miRNAs by acting as competing endogenous RNAs (ceRNAs) or natural miRNA sponges, building another complex layer of the transcriptional regulatory network (68). As shown in **Table 3**, CA3-AS1 interacts with hsa-miR-93-5p, which in turn targets the NEDD4L (E3 ubiquitin-protein ligase NEDD4-like) protein and the transcriptional repressor MXI1 (Max-interacting protein 1); CA3-AS1, NEDD4L and MXI1 were upregulated during *L. infantum* infection. The ceRNA regulator function of CA3-AS1 was described elsewhere (66), where it was found to be an anti-oncogene in gastric cancer by sponging hsa-miR-93-5p. Interactions with miRNAs were found for the other 6 lncRNAs, of which 5 interacted with multiple miRNAs. The upregulated hub GASAL1 (growth arrest-associated lncRNA 1) presented the highest number of miRNA interactions and was the only lncRNA correlated with IFNG expression. GASAL1 has been shown to be involved in the inhibition of tumor growth in lung cancer and may improve chronic heart failure by downregulating the TGF-β signaling pathway (69, 70).

The upregulated lncRNAs, AL139220.2 (novel transcript) and KDM7A-DT (KDM7A divergent transcript), were predicted to interact with the same five transcriptional regulators that were coexpressed with LYL1 (Protein lyl-1), KLF1 (Kruppel-like factor 1), PBX1 (Pre-B-cell leukemia transcription factor 1), TAL1 (T-cell acute lymphocytic leukemia protein 1) and MXI1 (Max-interacting protein 1), with the latter being a repressor of target genes. PBX1 is involved in natural killer cell differentiation (GO:0001779), whereas LYL1 plays a role in B cell differentiation (GO:0030183). KLF1 and TAL1 are transcription regulators of hemopoietic differentiation. KDM7A-DT, also known as JHDM1D-AS1, has been suggested to play a protective role during ROS-induced apoptosis in periodontal ligament stem cells (71). Experimental validation data for AL139220.2 were not found.

LYL1 and PBX1 were also interactors of the downregulated PSMA3-AS1 (PSMA3 antisense RNA 1), a lncRNA that presented predicted interactions with 4 miRNAs, including hsa-miR-105-5p, which targets TLR10 (downregulated in VL patients). PSMA3-AS1 was a DE lncRNA only in the comparison of PD180 vs. PD0 (**Supplementary Table 3**). Recently, PSMA3-AS1 has been validated as a ceRNA involved in the malignant phenotypes of esophageal cancer by modulating the miR-101/EZH2 axis (72). EZH2 (enhancer of zeste 2 polycomb repressive complex 2 subunit) is a histone methyltransferase and is another transcriptional repressor found as an interactor of our lncRNA network. In addition, PSMA3-AS1 has been discovered to sponge miR-409-3p and is considered an oncogenic lncRNA involved in the aggressive phenotype of non−small cell lung carcinoma (73).

Other coexpressed transcription factors commonly found as interactors of lncRNAs were the downregulated transcriptional repressor MXD1 (Max dimerization protein 1) and the E2F family of activators and repressors, whose coexpressed genes (E2F2, E2F7 and E2F8) were all upregulated in VL patients. The activity of E2F members is critical for transcriptional machinery throughout the cell cycle and cytokinesis (74). E2F2 is one of the transcription activators signed as a gene signature of the Th1 immune response (75). Finally, we highlight the upregulated lncRNA MIR4435-2HG, which was predicted to interact with 6 miRNAs, and BATF (basic leucine zipper ATF-like transcription factor), a transcription regulator that controls the differentiation of lymphocytes, specifically on switch isotypes in B cells and Th17 cells, follicular T-helper (TfH) cells, and CD8^+^ dendritic cells by interacting with members of the interferon-regulatory factor (IRF) family (76). Moreover, BATF plays an essential role during hematopoiesis and the homeostasis of effector functions of innate lymphoid cells (ILCs) (77), which are innate counterparts of T cells and are predominantly situated at the mucosal barriers (78). To the best of our knowledge, this study is the first to infer a lncRNA–TF interaction between BATF and MIR4435-2HG within a transcriptional network regulation of a human infectious disease. Furthermore, MIR4435-2HG bound to EZH2 and promoted hepatocellular carcinoma progression *via* EZH2-mediated epigenetic silencing of p21 and E-cadherin expression (79). MIR4435-2HG lncRNA has been extensively studied in recent years, with 41 related articles in PubMed (https://www.ncbi.nlm.nih.gov/gene/541471), in which 34 of them have shown experimental validation of its role in tumor progression and as a prognostic biomarker in different types of cancer. It is also known as AGD2, LINC00978, MIR4435-1HG, MORRBID and lncRNA-AWPPH and acts as a ceRNA by sponging many other miRNAs (not listed in **Table 3**), such as miR-296-5p, which was identified to be part of the Akt2/SNAI1 signaling pathway involved in the development of oral squamous cell carcinoma triggered by *Fusobacterium nucleatum* infection (80).

Among the hub lncRNAs featured in our work, some of them have been shown to be stable and detectable in blood plasma samples and circulating exosomes elsewhere, such as MALAT1, MIR4435-2HG, and PSMA3-AS1 (81–83), which may promptly favor their potential as blood biomarkers. For other lncRNAs, such as IL21-AS1, AC111000.4, CA3-AS1, GASAL1, and LINC01501, no literature associated with plasma or circulating exosomes was found. However, since this study was not designed for diagnosis purpose, future studies should be performed to evaluate these lncRNAs as new avenue to be explored in VL. The significance of lncRNAs as master regulators of many biological processes in health and diseases is well established, and their application as biomarkers for disease progression has been rapidly increased in cancer biology but is still incipient in parasitic diseases. Furthermore, lncRNAs have been found in body fluids, freely or inside exosomes (84). Detecting eligible lncRNAs in plasma and/or serum is a promising noninvasive and affordable method for prognosis, and many studies with tumors have shown its value in either complementary diagnosis or aggressiveness prediction (84, 85).

## Concluding Remarks

For the first time, an integrated analysis of lncRNAs and protein-coding genes (mRNAs) was performed in human visceral leishmaniasis using blood transcriptomics. Blood transcriptomes obtained by mRNA-seq allowed us to surpass the typical analyses comprising gene pathways and protein networks, adding an extra and important layer to this big picture captured by transcriptomics, the long noncoding RNA profile. From a comprehensive analysis, we highlighted lncRNAs such as MALAT1, CA3-AS1, GASAL1, PSMA3-AS1, MIR4435-2HG, IL21-AS1, AC111000.4, and LINC01501, with these last three being the most regulated lncRNAs compared active VL to the VL-free groups. Moreover, by comparing VL patients before and after a six-month follow-up, this study suggests there is a potential for use lncRNAs in plasma and/or serum as marker for monitoring disease remission. Focusing on a set of differentially expressed genes, the lncRNA–mRNA coexpression profile presented here was able to provide valuable and insightful data to help unravel the complexity of host/parasite interactions in human visceral leishmaniasis caused by *L. infantum* infection. We believe that our study will be useful to guide future studies for searching lncRNAs as biomarkers, new targets for drugs or drug repurposing and new therapies to control this neglected disease.

## Data Availability Statement

Metadata and fastq files from the RNA sequencing experiment were deposited in the functional 582 genomics repository, ArrayExpress from EMBL-EBI, under accession number E-MTAB-11047 583 (available at: http://www.ebi.ac.uk/arrayexpress/E-MTAB-11047/).

## Ethics Statement

The studies involving human participants were reviewed and approved by the Brazilian Human Research Ethics Evaluation System (CEP/CONEP; CAAE: 04587312.2.0000.0058). Written informed consent to participate in this study was provided by own parcipants if in adult age or by the legal guardian/next of kin of the participants if under 18 years of age.

## Author Contributions

Conceived the study: RPA and JSS. Supervised the study: SRM, RPA, HN, and JSS. Designed the experiments: SRM, RPA, and JSS. Generated clinical samples and management of clinical data: RPA, ARJ, EVM, and AMS. Performed the experiments: SRM and GP. Analyzed data: SRM, CAF, AERO, LAR, NTT, VC, and HN. Performed statistical data analysis: SRM, CAF, and AERO. Contributed reagents/material/analysis tools: SRM, ARJ, RPA, and JSS. Sketched figures and tables: SRM, LAR, NTT, CAF, and AERO. Wrote the manuscript: SRM. All authors listed have made a substantial, direct, and intellectual contribution to the work and approved it for publication.

## Funding

This work was supported by grants from the Fundação de Amparo à Pesquisa do Estado de São Paulo (FAPESP agreements 2016/20258-0 (Young Investigator Award to SM), 2018/14933-2 (HN), and 2013/08216-2 (Center for Research in Inflammatory Diseases); the Coordenação de Aperfeiçoamento de Pessoal de Nível Superior—Brasil (CAPES) grant no. 23038.005304/2011-01 and Finance code 001; the Conselho Nacional de Desenvolvimento Científico e Tecnológico (CNPq) grant no. 552721/2011-5 (RPA); fellowship/grant no. 307741/2017-6 to AJ. SM and AO received fellowships from the FAPESP (2017/16328-6 and 2019/22579-7, respectively). LR received a scholarship from the FAPESP (2018/26799-9 and 2020/14011-8), and NT received scholarships from the FAPESP (2021/12464-8) and the CNPq (133661/2020-2).

## Conflict of Interest

The authors declare that the research was conducted in the absence of any commercial or financial relationships that could be construed as a potential conflict of interest.

## Publisher’s Note

All claims expressed in this article are solely those of the authors and do not necessarily represent those of their affiliated organizations, or those of the publisher, the editors and the reviewers. Any product that may be evaluated in this article, or claim that may be made by its manufacturer, is not guaranteed or endorsed by the publisher.

## Supplementary Material

Presentation 1 CEMiTool html report with results for coexpression modules of RNA-seq analysis of blood in human visceral leishmaniasis caused by *L. infantum* infection.

Supplementary Figure 1 Heterogeneity of samples analyzed using the **M**olecular **D**egree of **P**erturbation (MDP) tool. **(A)** Molecular perturbation scores plotted for each sample based on 14,247 genes expressed across the four groups. DEGs shared among all comparisons of VL patients to nondiseased groups (as numbers in **(Figure 3)**, central overlap); **(B–D)** Boxplots representing MDP scores by group quantified based on three subsets of DEGs, where the first was composed of 84 DEGs shared between A vs. PD0 and PD180 vs. PD0 comparisons (b), the second was composed of 186 DEGs exclusive for PD180 vs. PD0 comparison (c) and the third was composed of 193 DEGs exclusive for A vs. PD0 comparison.

Supplementary Figure 2 Cellular deconvolution of blood transcriptomes in human visceral leishmaniasis using the CIBERSORT method. **(A)** Leukocyte proportions inferred from gene expression profiles of blood samples from Gardinassi et al., 2016 (microarray). **(B)** Plots by cell type displaying the relative cell proportions for neutrophils, eosinophils, plasma cells, memory CD4 cell plasma cells, CD8 T cells, monocytes, activated NK cells and resting NK cells. In the study of Gardinassi et al., groups were classified as CTRL (healthy uninfected controls, n= 15), DTH (asymptomatic patients, n= 14), TRT (patients at 2-5 months after treatment, considered under remission of the disease; n= 8) and VL (diseased patients, n= 8).

Supplementary Table 1 Excel spreadsheets with results for differential expression analysis for pairwise comparisons between nondiseased groups PD180 (cured VL patients, 180 days after treatment), A (asymptomatic subjects) and C (healthy controls) against PD0 (active VL patients). Only differentially expressed genes (DEGs) at FDR <0.05 with -1< log2-fold-change > +1 (twofold decrease or increase) were included for further analysis.

Supplementary Table 2 Annotation of 1,147 lncRNAs expressed in RNA-seq in this work. The main features were retrieved from the GRCh38.p13 human genome version using the BioMart Ensembl LNCipedia database.

Supplementary Table 3 Annotation of 147 selected differentially expressed (DE) lncRNAs in RNA-seq in this work. The main features were retrieved from the GRCh38.p13 human genome version using the BioMart Ensembl, LNCipedia and Expression Atlas databases.

Supplementary Table 4 Top50 lncRNAs selected from 147 DE lncRNAs (**Supplementary Table 3** spreadsheet) ranked by statistical significance (FDR values) followed by fold regulation (log_2_FC values) in VL patients compared to nondiseased groups.

Supplementary Table 5 Highly correlated pairs of lncRNA-mRNA retrieved by Pearson’s correlation using log(cpm) read count for 147 DE lncRNAs and 1,263 DE mRNAs from all samples. The cutoff for the correlation coefficient was -0.8 < *r* > 0.8; all results presented a *p value* < 6e^-10^. Hubs from networks and Reactome pathways are also tabulated herein.

Supplementary Table 6 Catalog of lncRNA subcellular location and interaction partners searched for lncRNAs featuring hubs in network analysis **(Figure 7)** and predicted as *cis*-acting lncRNAs **(Figure 8)**. Data mining was performed using the LncSLdb and RNA Inter databases and the LncLocator prediction tool.
