# Supplementary material for: Insight Into the Long Noncoding RNA and mRNA Coexpression Profile in the Human Blood Transcriptome Upon Leishmania infantum Infection
Source: Front Immunol. 2022 Mar 15;13:784463. doi: 10.3389/fimmu.2022.784463 (PMC8965071; doi:10.3389/fimmu.2022.784463)
Supplement: Presentation 1 — CEMiTool html report with results for coexpression modules of RNA-seq analysis of blood in human visceral leishmaniasis caused by L. infantum infection. [file Presentation_1.pdf]

Code

- Show All Code
- Hide All Code

CEMiTool

Report

Modules

Profile Plot

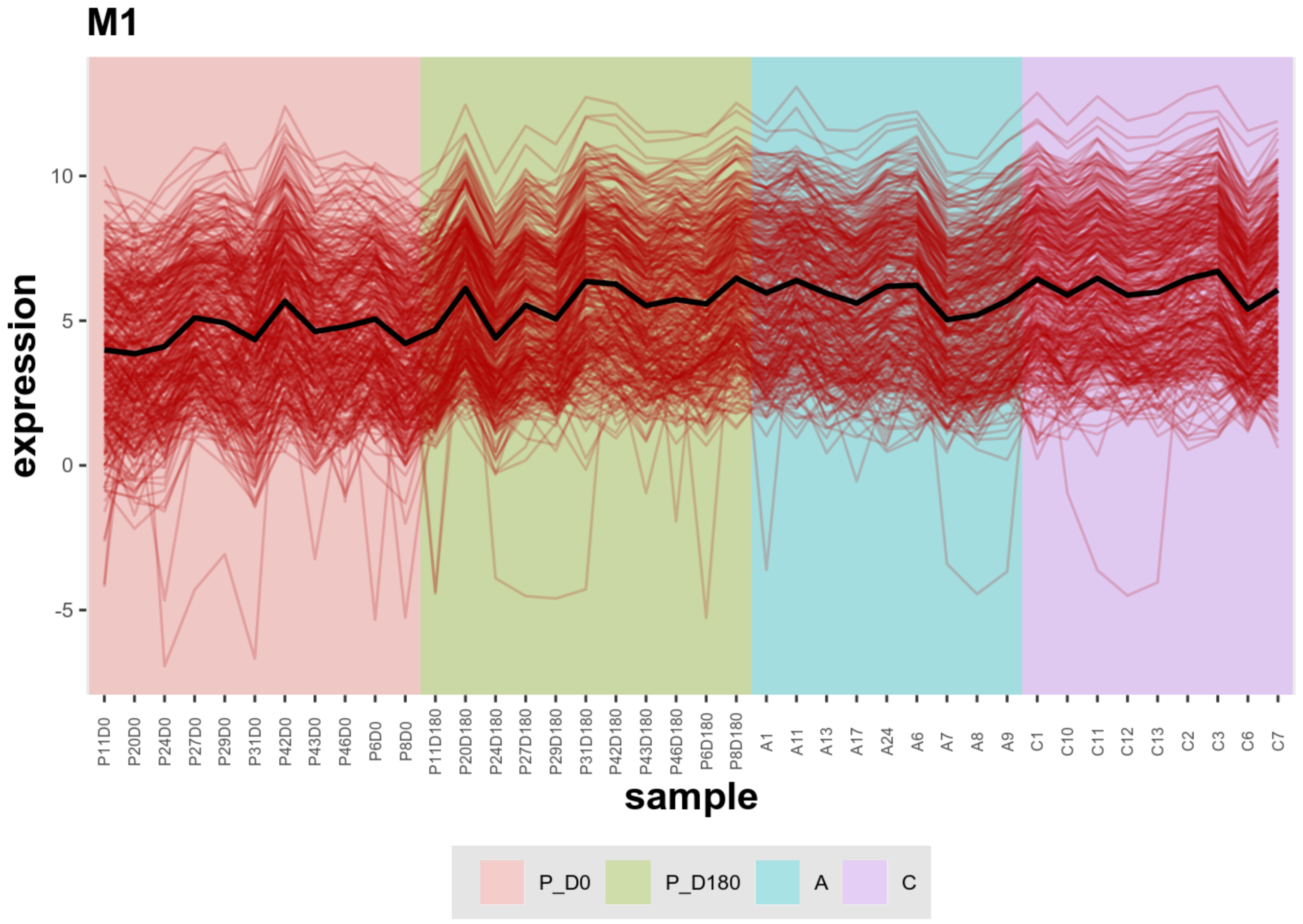
M1
expression
sample
10
5
0
-5
P11D0
P20D0
P24D0
P27D0
P29D0
P31D0
P42D0
P43D0
P46D0
P6D0
P8D0
P11D180
P20D180
P24D180
P27D180
P29D180
P31D180
P42D180
P43D180
P46D180
P6D180
P8D180
A1
A11
A13
A17
A24
A6
A7
A8
A9
C1
C10
C11
C12
C13
C2
C3
C6
C7
P_D0
P_D180
A
C

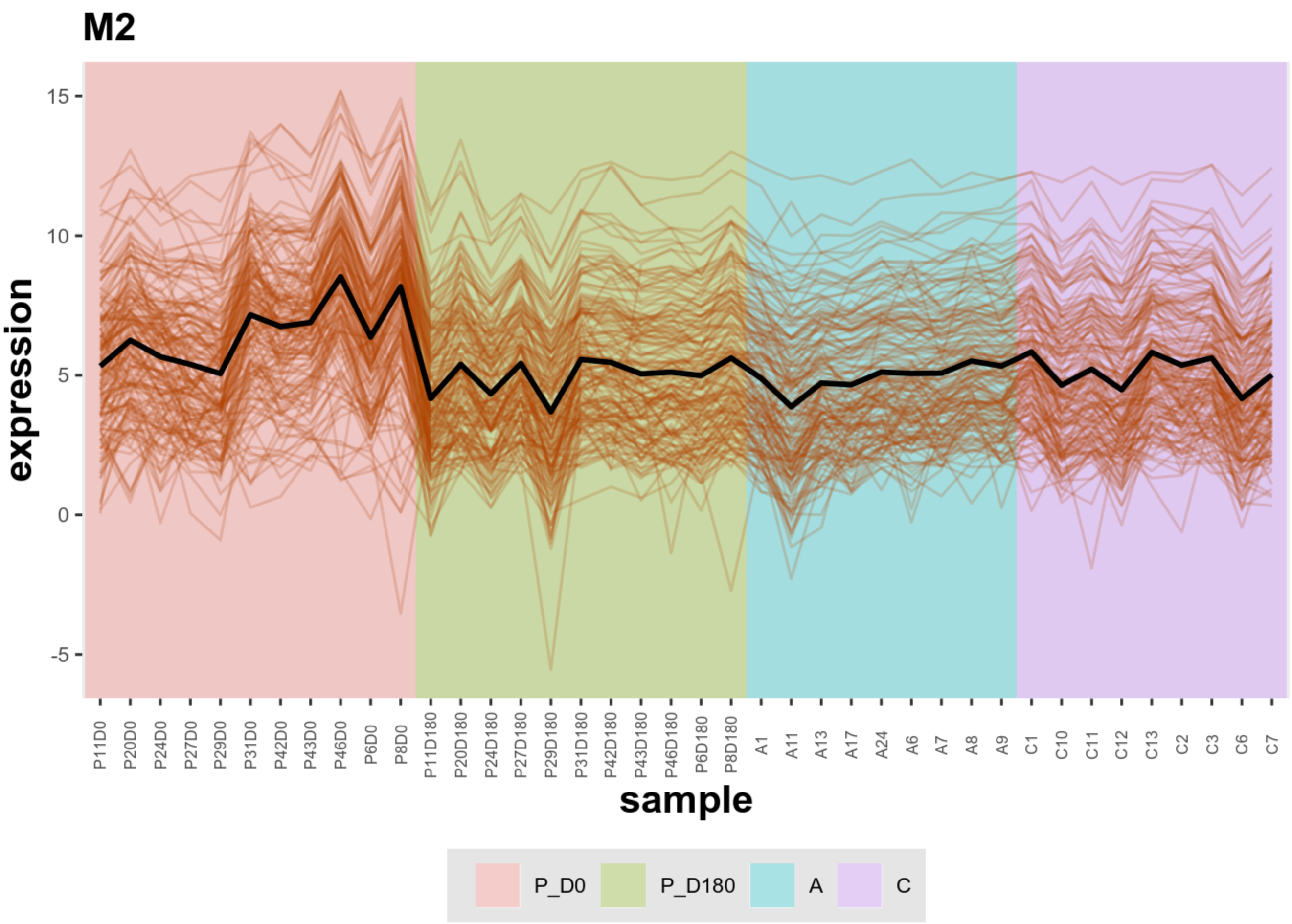
M2
expression
15
10
5
0
-5
P11D0
P20D0
P24D0
P27D0
P29D0
P31D0
P42D0
P43D0
P46D0
P6D0
P8D0
P11D180
P20D180
P24D180
P27D180
P29D180
P31D180
P42D180
P43D180
P46D180
P6D180
P8D180
A1
A11
A13
A17
A24
A6
A7
A8
A9
C1
C10
C11
C12
C13
C2
C3
C6
C7
sample
P_D0
P_D180
A
C

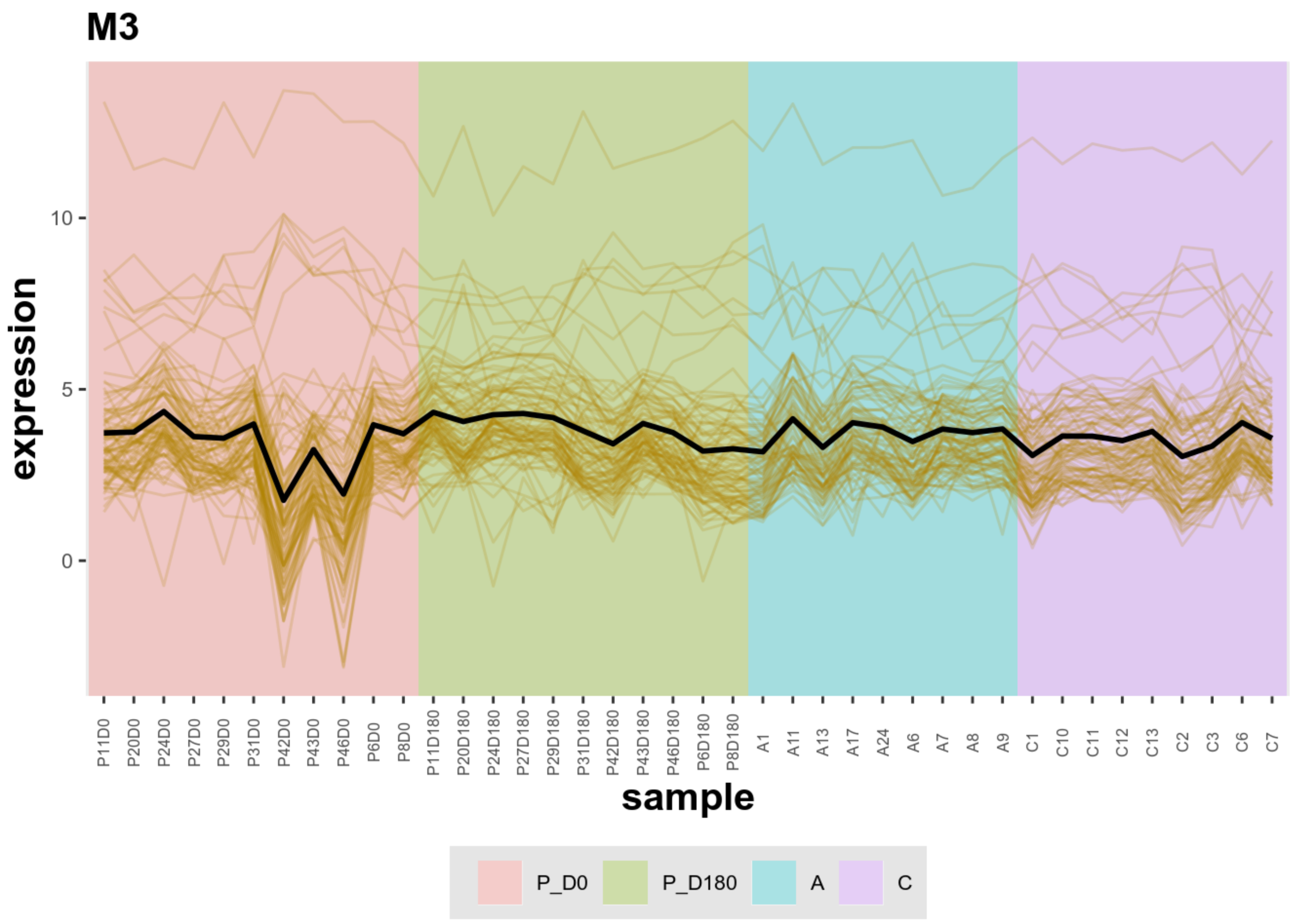
M3
expression
10
5
0
P11D0
P20D0
P24D0
P27D0
P29D0
P31D0
P42D0
P43D0
P46D0
P6D0
P8D0
P11D180
P20D180
P24D180
P27D180
P29D180
P31D180
P42D180
P43D180
P46D180
P6D180
P8D180
A1
A11
A13
A17
A24
A6
A7
A8
A9
C1
C10
C11
C12
C13
C2
C3
C6
C7
sample
P_D0
P_D180
A
C

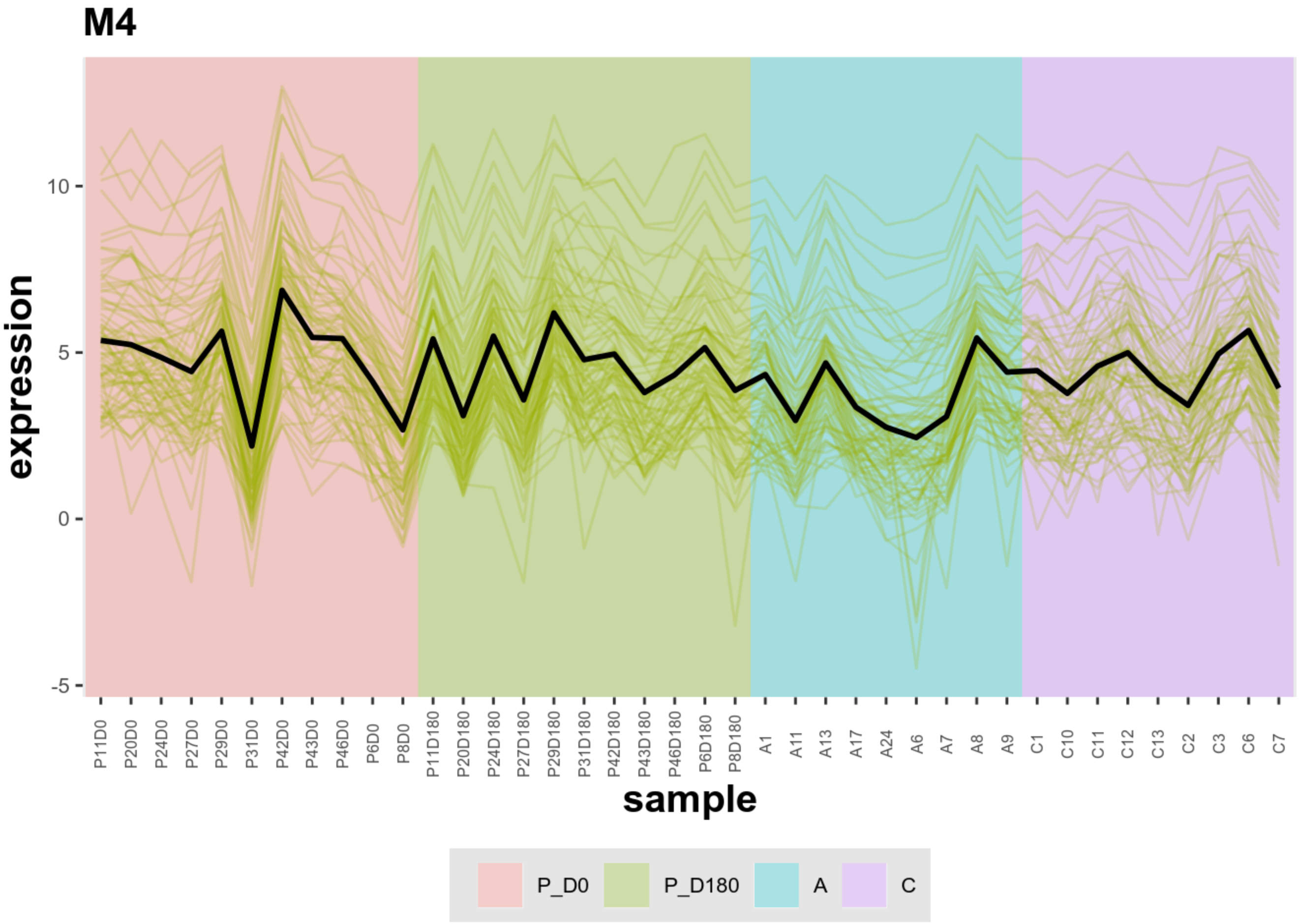
M4
expression
10
5
0
-5
P11D0
P20D0
P24D0
P27D0
P29D0
P31D0
P42D0
P43D0
P46D0
P6D0
P8D0
P11D180
P20D180
P24D180
P27D180
P29D180
P31D180
P42D180
P43D180
P46D180
P6D180
P8D180
A1
A11
A13
A17
A24
A6
A7
A8
A9
C1
C10
C11
C12
C13
C2
C3
C6
C7
sample
P_D0
P_D180
A
C

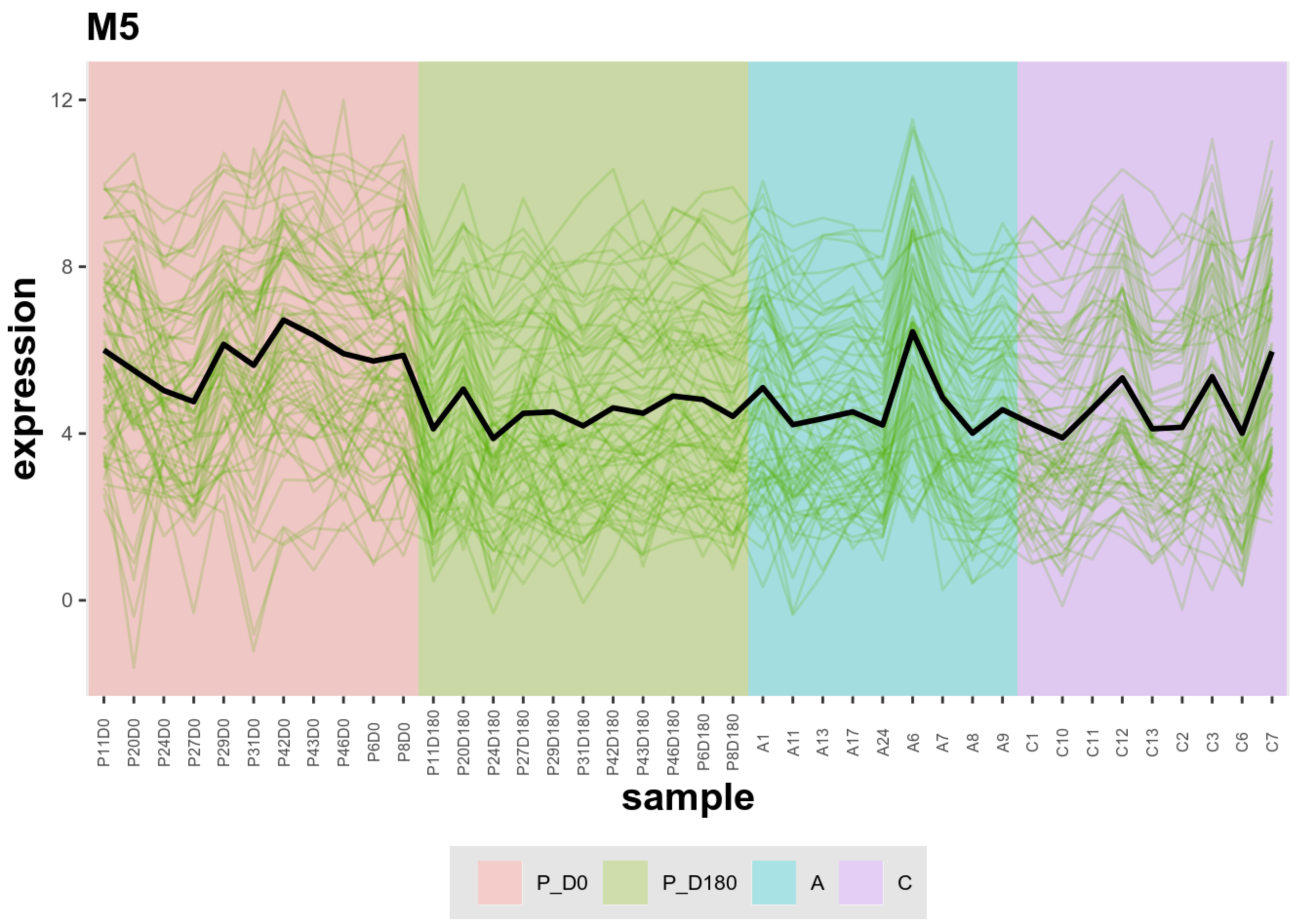
M5
expression
12
8
4
0
P11D0
P20D0
P24D0
P27D0
P29D0
P31D0
P42D0
P43D0
P46D0
P6D0
P8D0
P11D180
P20D180
P24D180
P27D180
P29D180
P31D180
P42D180
P43D180
P46D180
P6D180
P8D180
A1
A11
A13
A17
A24
A6
A7
A8
A9
C1
C10
C11
C12
C13
C2
C3
C6
C7
sample
P_D0
P_D180
A
C

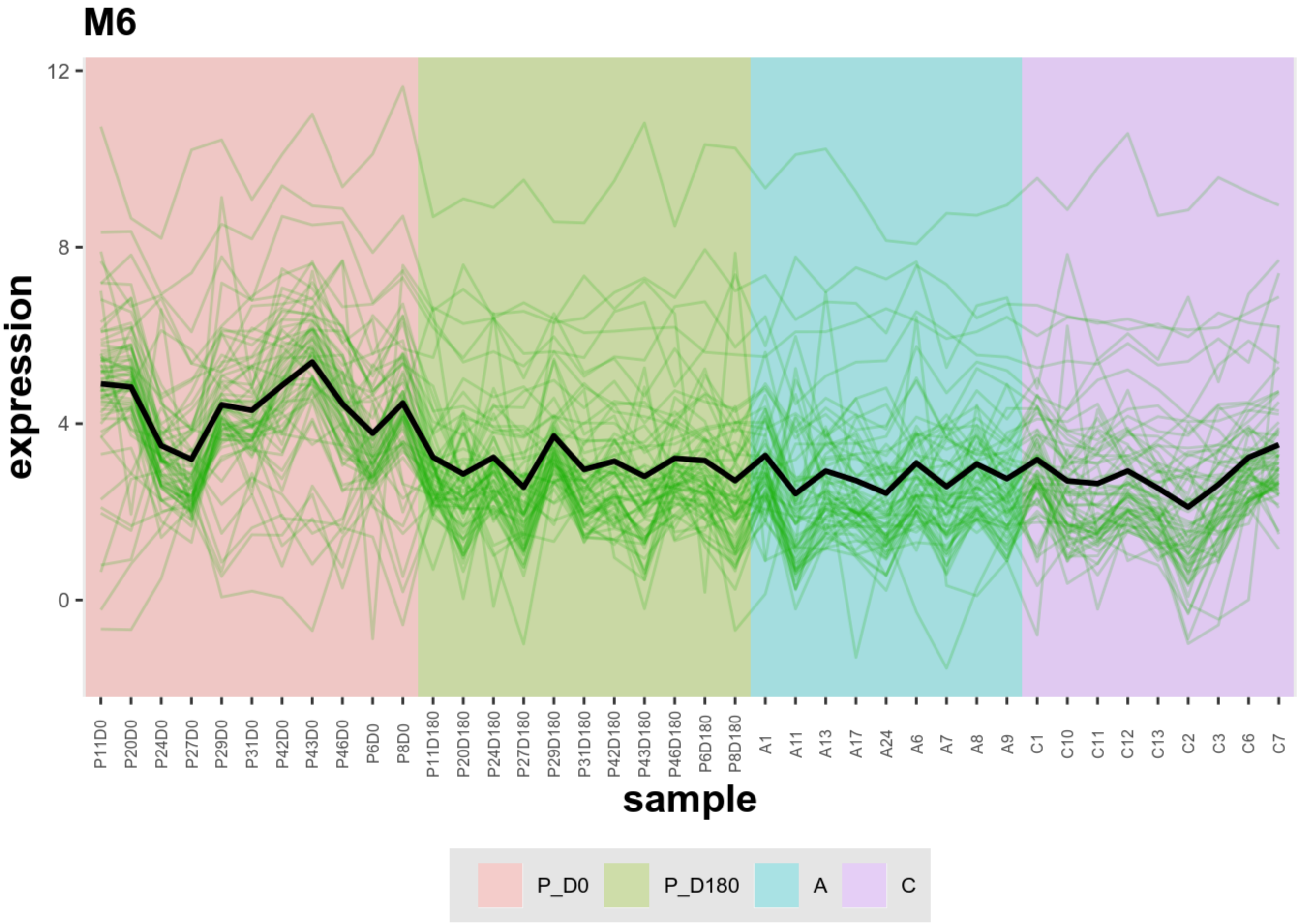

M6
expression
sample
12
8
4
0
P11D0
P20D0
P24D0
P27D0
P29D0
P31D0
P42D0
P43D0
P46D0
P6D0
P8D0
P11D180
P20D180
P24D180
P27D180
P29D180
P31D180
P42D180
P43D180
P46D180
P6D180
P8D180
A1
A11
A13
A17
A24
A6
A7
A8
A9
C1
C10
C11
C12
C13
C2
C3
C6
C7
P_D0
P_D180
A
C

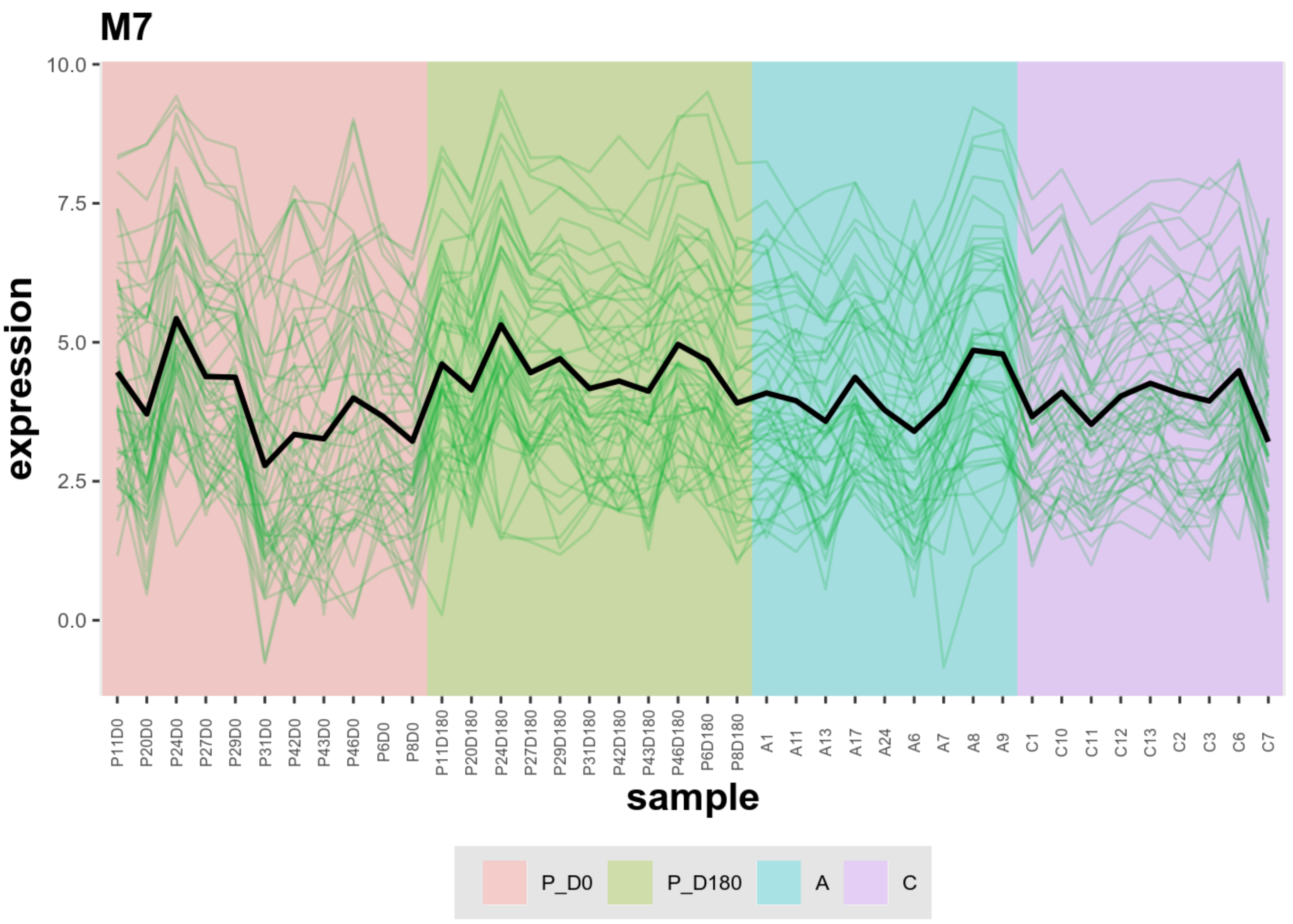
M7
10.0
7.5
5.0
2.5
0.0
expression
P11D0
P20D0
P24D0
P27D0
P29D0
P31D0
P42D0
P43D0
P46D0
P6D0
P8D0
P11D180
P20D180
P24D180
P27D180
P29D180
P31D180
P42D180
P43D180
P46D180
P6D180
P8D180
A1
A11
A13
A17
A24
A6
A7
A8
A9
C1
C10
C11
C12
C13
C2
C3
C6
C7
sample
P_D0
P_D180
A
C

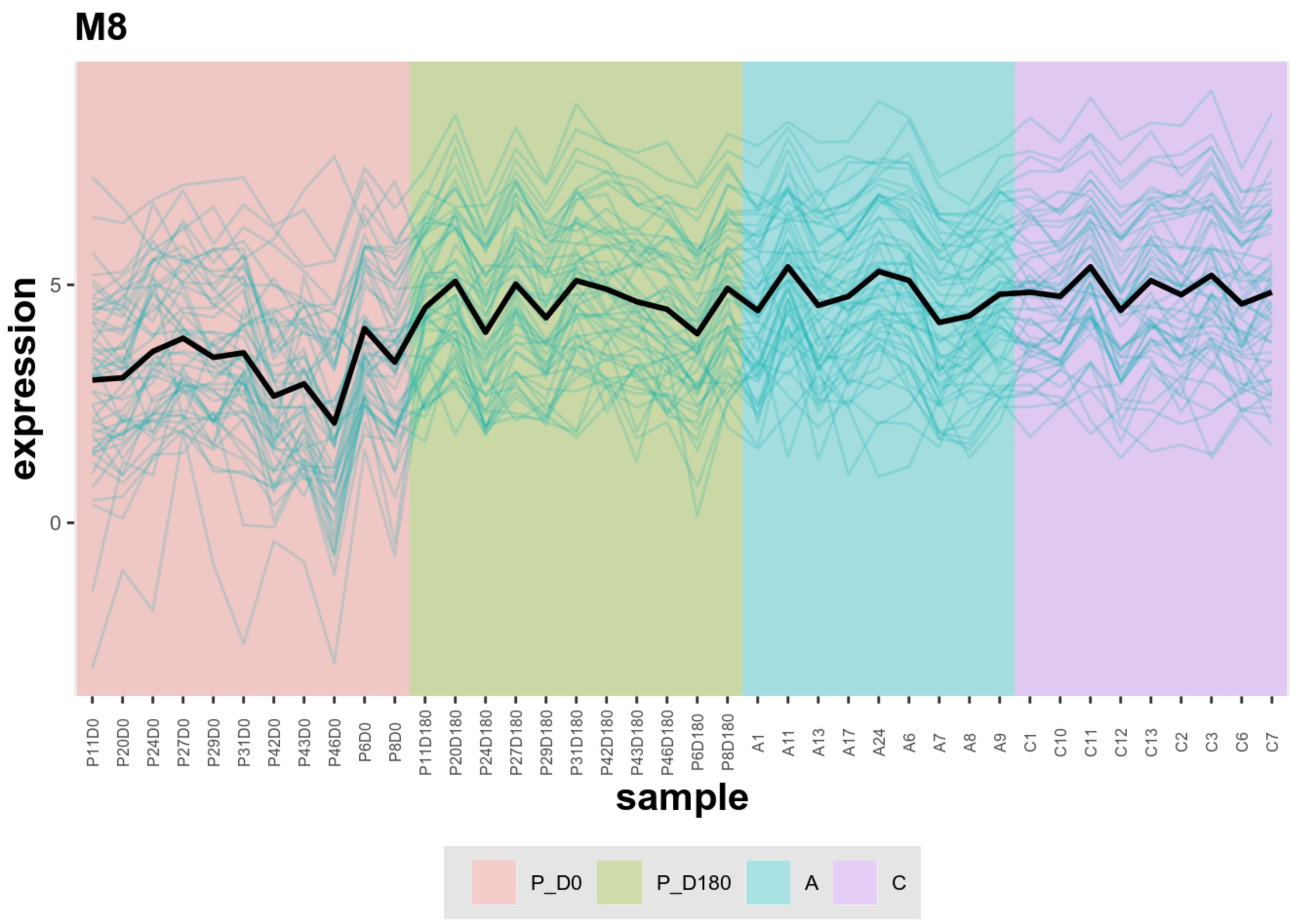

M8
expression
5
0
P11D0
P20D0
P24D0
P27D0
P29D0
P31D0
P42D0
P43D0
P46D0
P6D0
P8D0
P11D180
P20D180
P24D180
P27D180
P29D180
P31D180
P42D180
P43D180
P46D180
P6D180
P8D180
A1
A11
A13
A17
A24
A6
A7
A8
A9
C1
C10
C11
C12
C13
C2
C3
C6
C7
sample
P_D0
P_D180
A
C

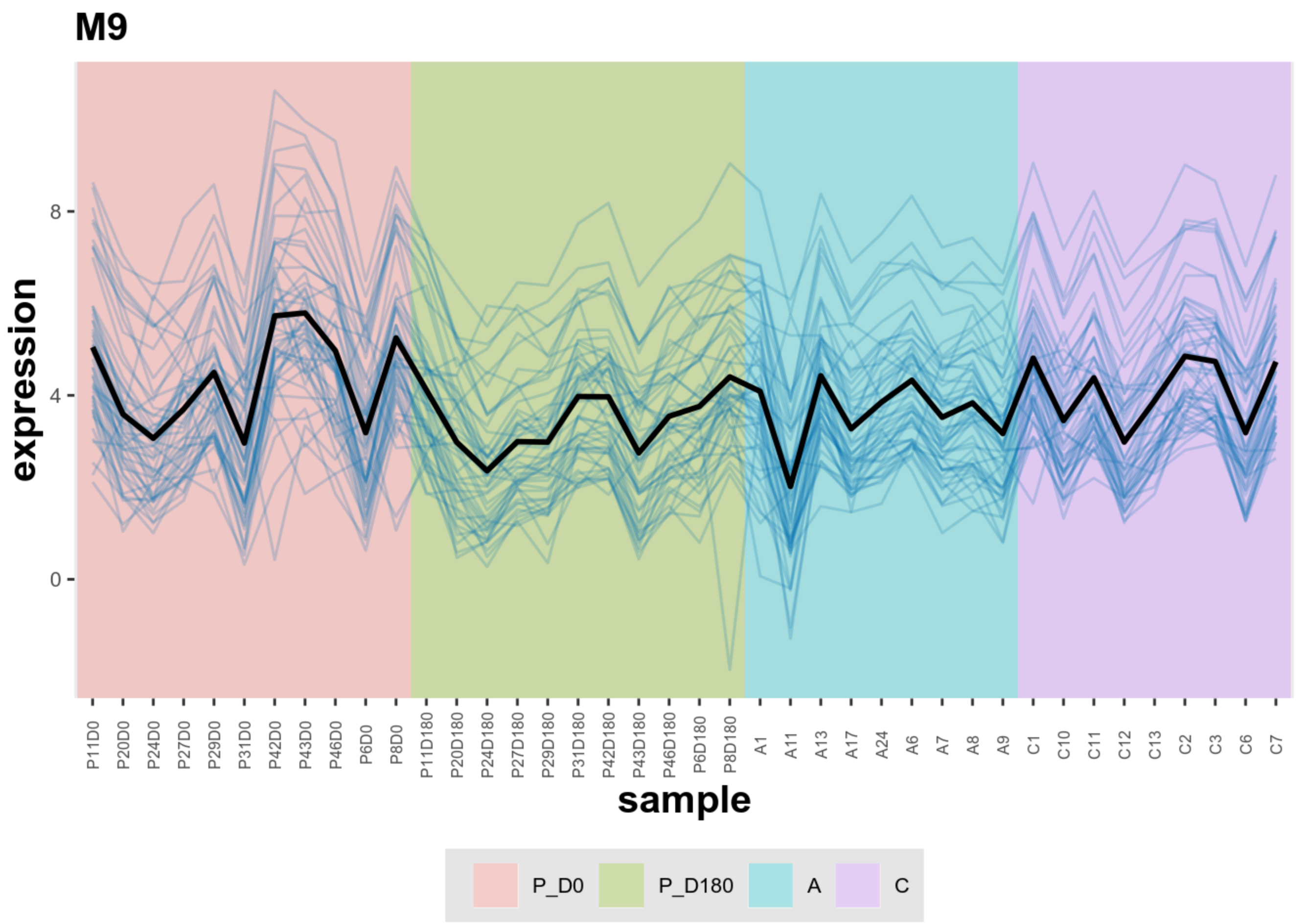
M9
expression
8
4
0
P11D0
P20D0
P24D0
P27D0
P29D0
P31D0
P42D0
P43D0
P46D0
P6D0
P8D0
P11D180
P20D180
P24D180
P27D180
P29D180
P31D180
P42D180
P43D180
P46D180
P6D180
P8D180
A1
A11
A13
A17
A24
A6
A7
A8
A9
C1
C10
C11
C12
C13
C2
C3
C6
C7
sample
P_D0
P_D180
A
C

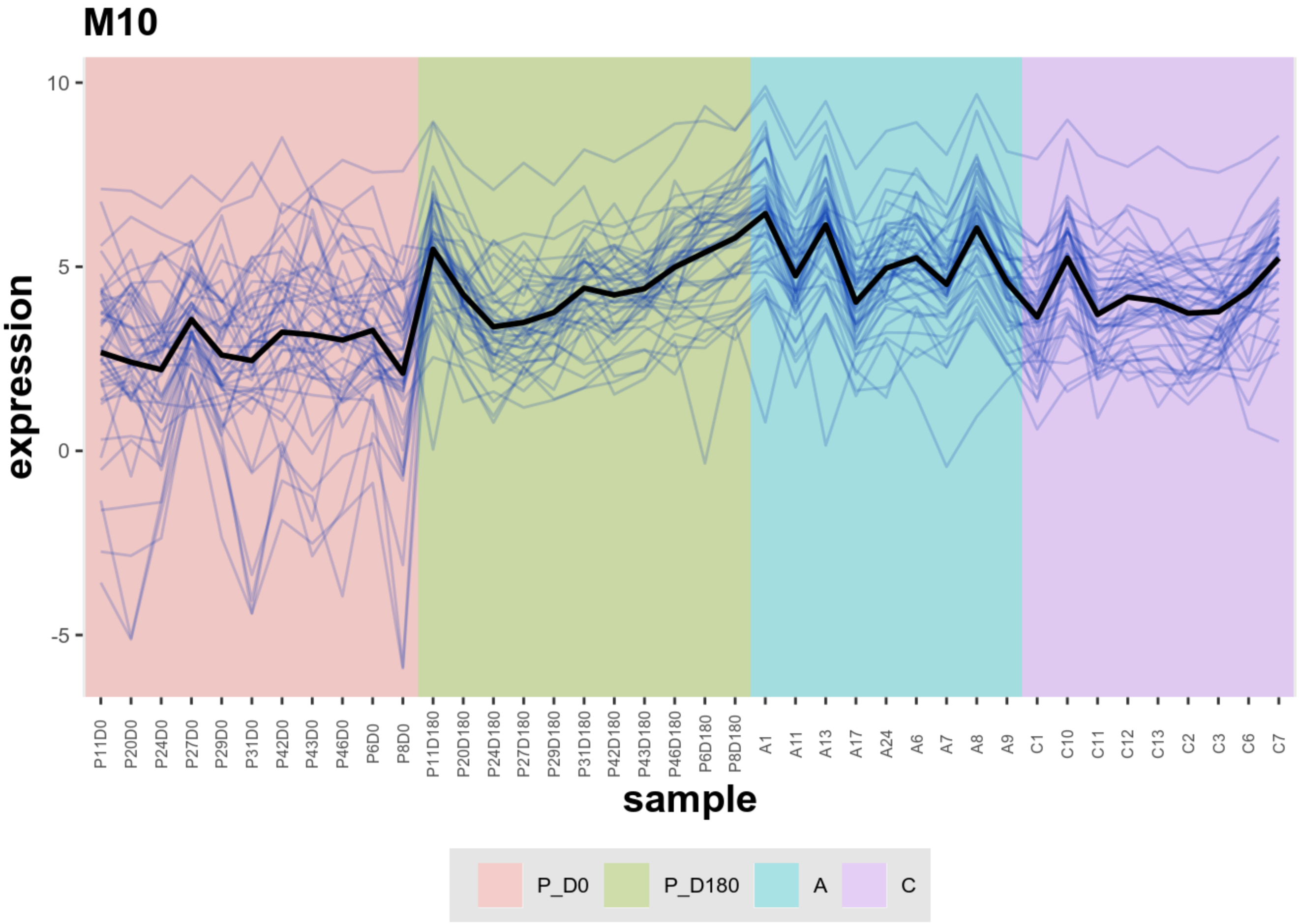

M10
expression
10
5
0
-5
sample
P11D0
P20D0
P24D0
P27D0
P29D0
P31D0
P42D0
P43D0
P46D0
P6D0
P8D0
P11D180
P20D180
P24D180
P27D180
P29D180
P31D180
P42D180
P43D180
P46D180
P6D180
P8D180
A1
A11
A13
A17
A24
A6
A7
A8
A9
C1
C10
C11
C12
C13
C2
C3
C6
C7
P_D0
P_D180
A
C

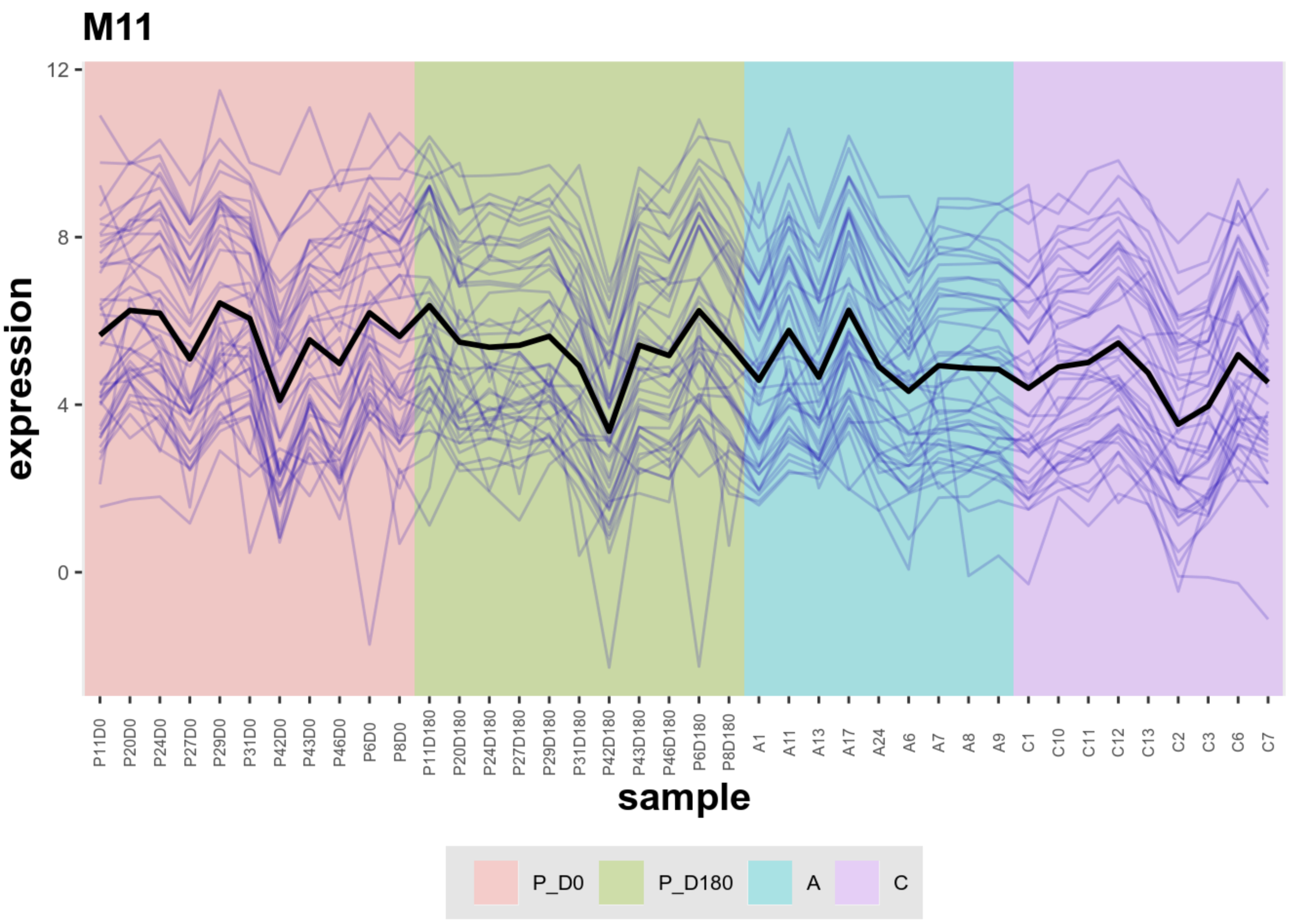
M11
expression
12
8
4
0
P11D0
P20D0
P24D0
P27D0
P29D0
P31D0
P42D0
P43D0
P46D0
P6D0
P8D0
P11D180
P20D180
P24D180
P27D180
P29D180
P31D180
P42D180
P43D180
P46D180
P6D180
P8D180
A1
A11
A13
A17
A24
A6
A7
A8
A9
C1
C10
C11
C12
C13
C2
C3
C6
C7
sample
P_D0
P_D180
A
C

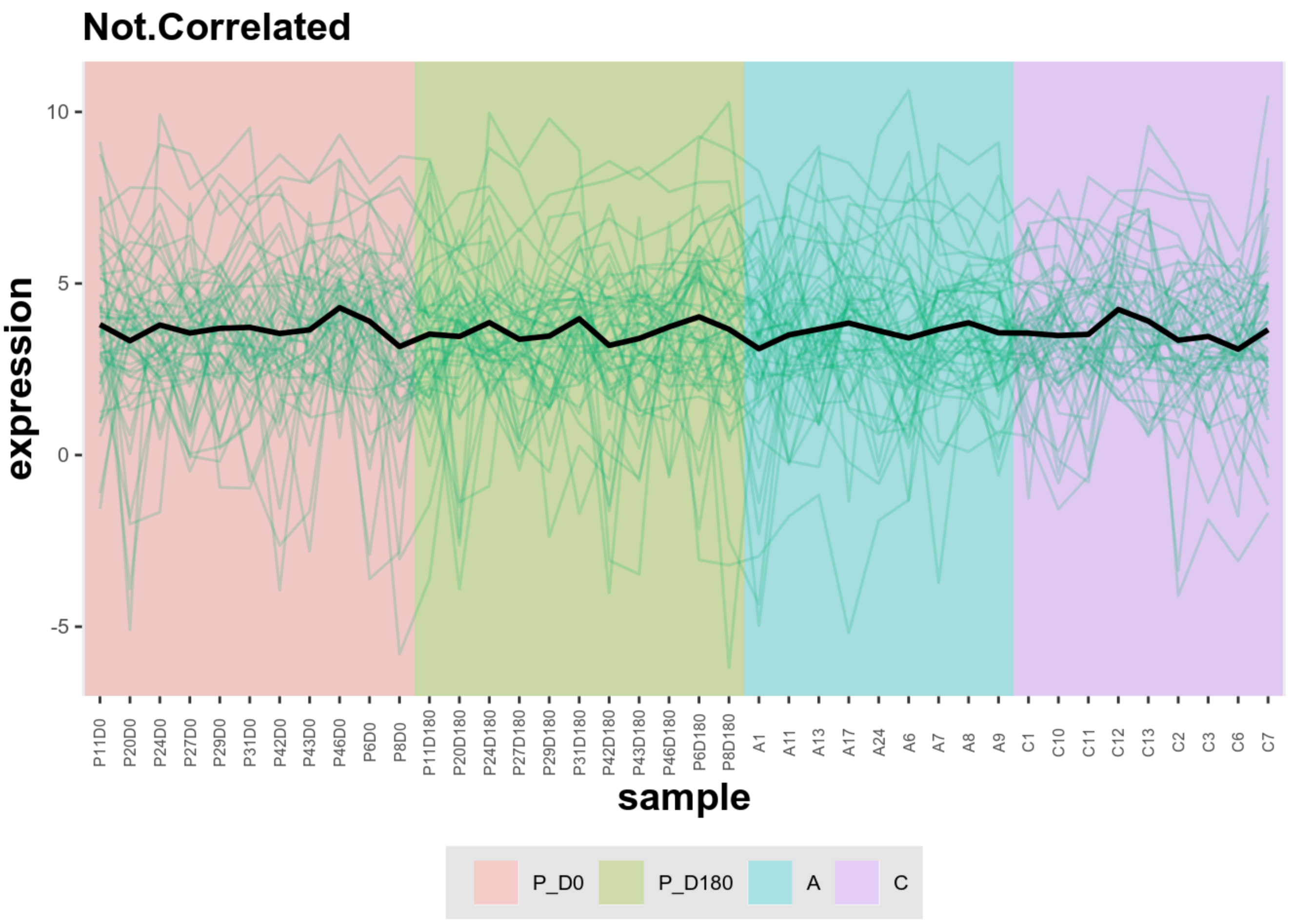

Gene Set Enrichment Analysis

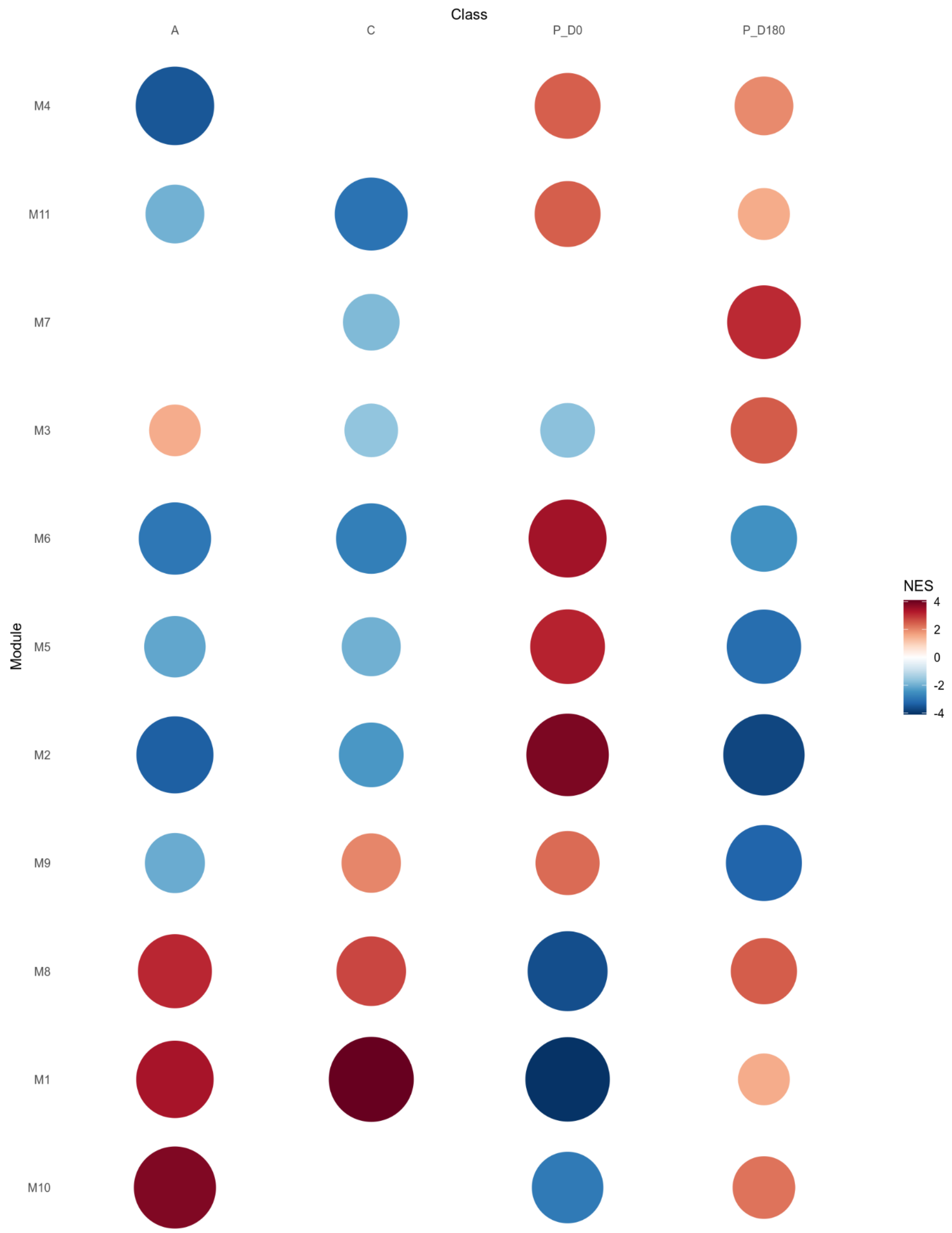

Over Representation Analysis

M1

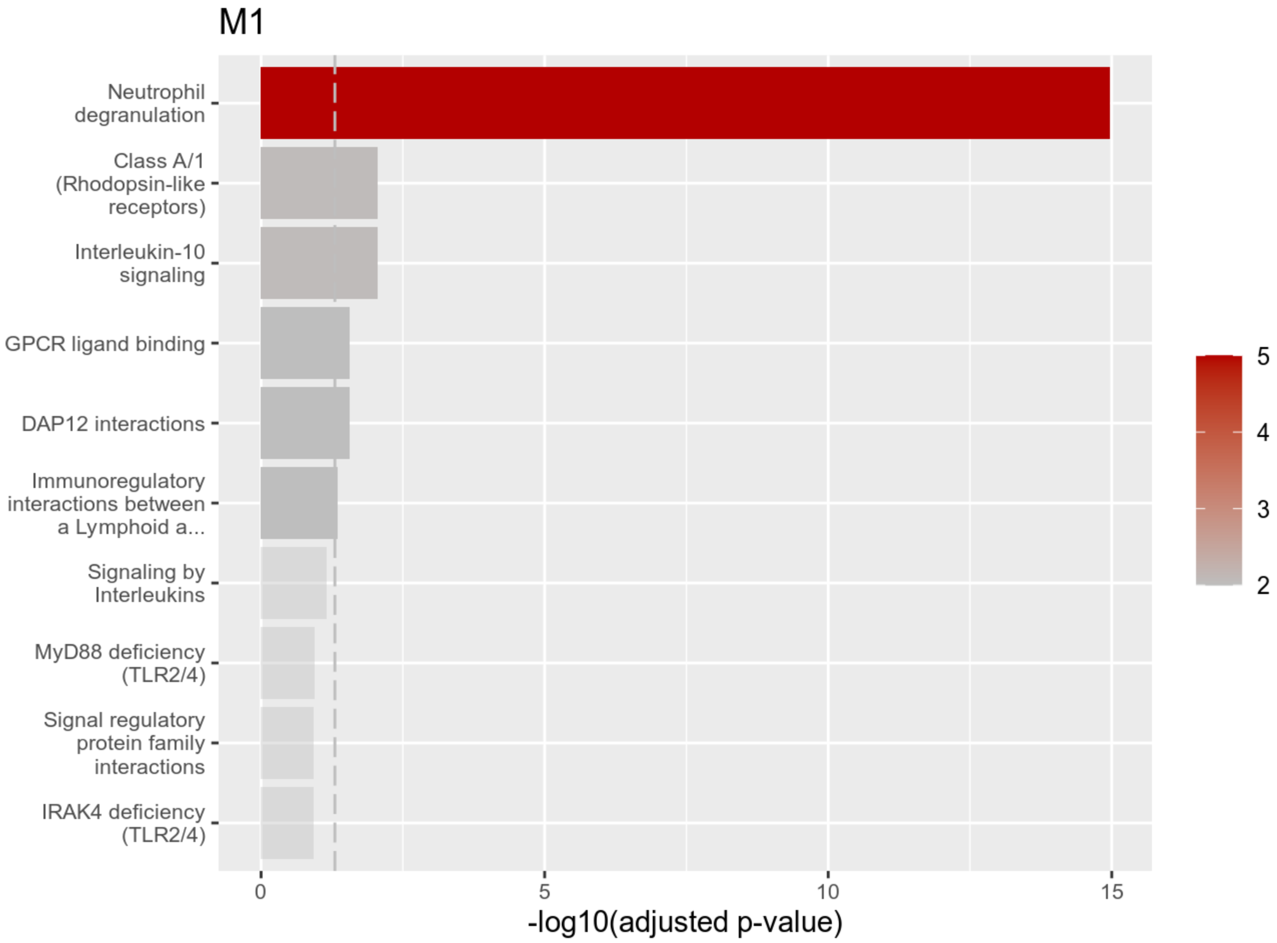
M1
Neutrophil degranulation
Class A/1 (Rhodopsin-like receptors)
Interleukin-10 signaling
GPCR ligand binding
DAP12 interactions
Immunoregulatory interactions between a Lymphoid a...
Signaling by Interleukins
MyD88 deficiency (TLR2/4)
Signal regulatory protein family interactions
IRAK4 deficiency (TLR2/4)
0
5
10
15
-log10(adjusted p-value)
5
4
3
2

M2

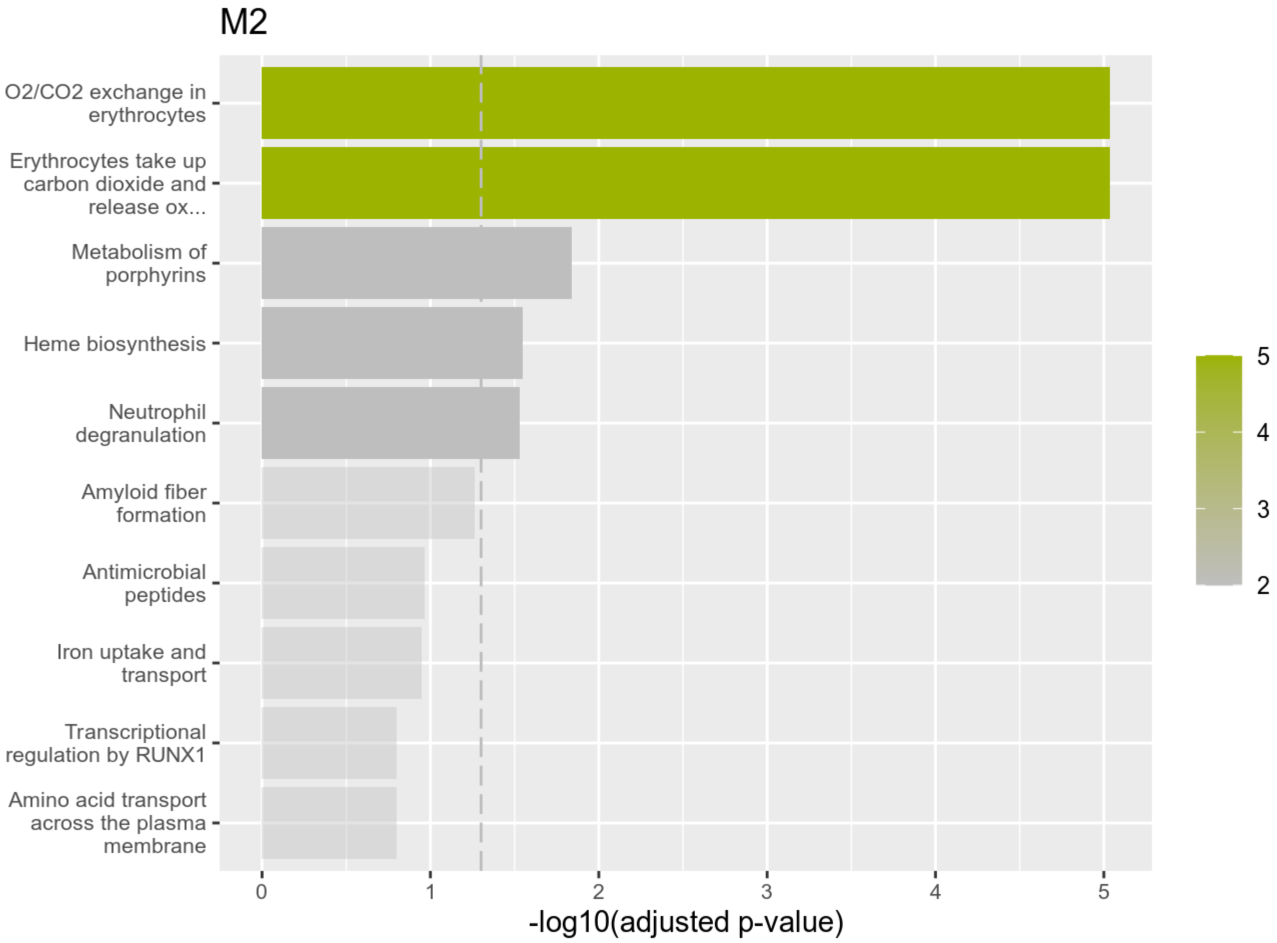

M2
O2/CO2 exchange in erythrocytes
Erythrocytes take up carbon dioxide and release ox...
Metabolism of porphyrins
Heme biosynthesis
Neutrophil degranulation
Amyloid fiber formation
Antimicrobial peptides
Iron uptake and transport
Transcriptional regulation by RUNX1
Amino acid transport across the plasma membrane
0
1
2
3
4
5
-log10(adjusted p-value)
5
4
3
2

M3

M3

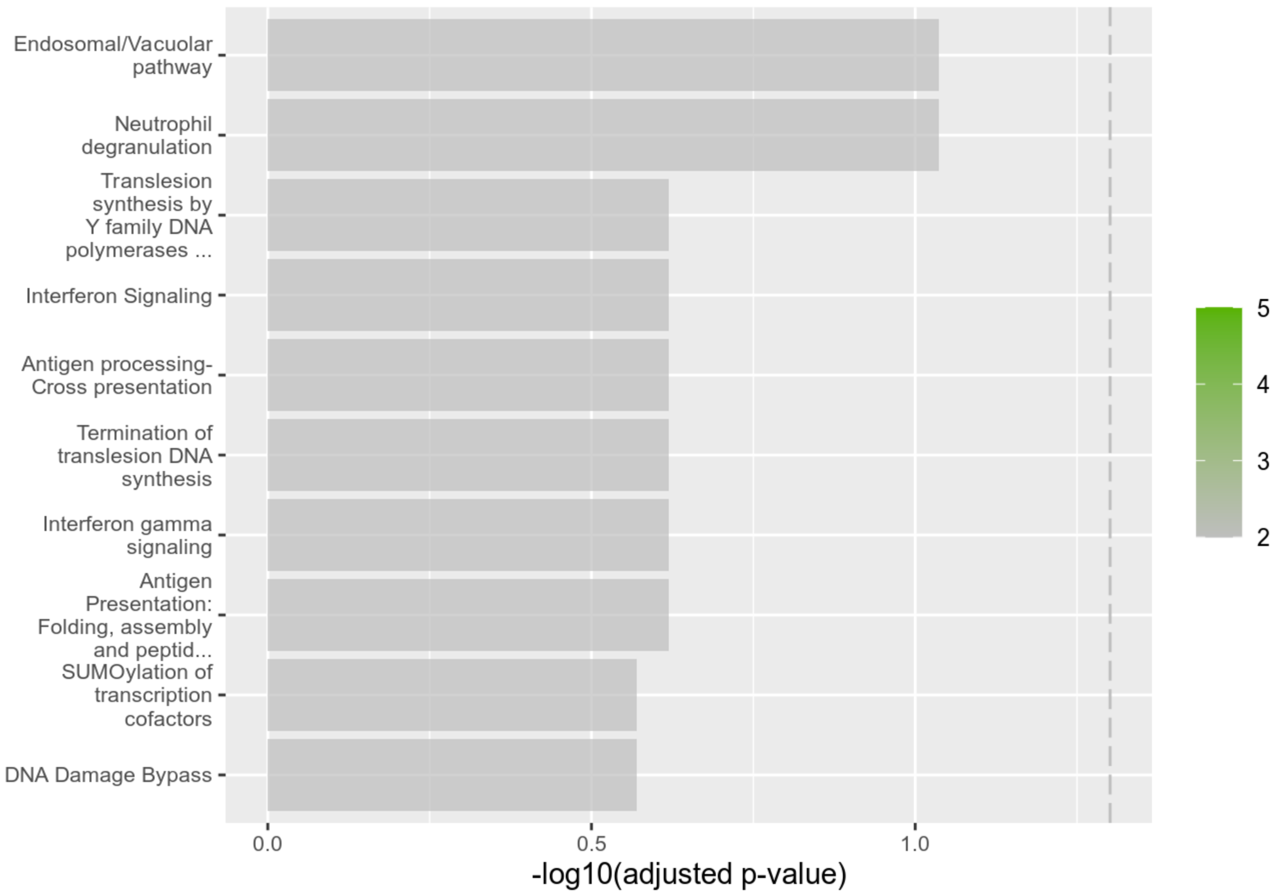
Endosomal/Vacuolar pathway
Neutrophil degranulation
Translesion synthesis by Y family DNA polymerases ...
Interferon Signaling
Antigen processing-Cross presentation
Termination of translesion DNA synthesis
Interferon gamma signaling
Antigen Presentation: Folding, assembly and peptid...
SUMOylation of transcription cofactors
DNA Damage Bypass
0.0
0.5
1.0
-log10(adjusted p-value)
5
4
3
2

M4

M4

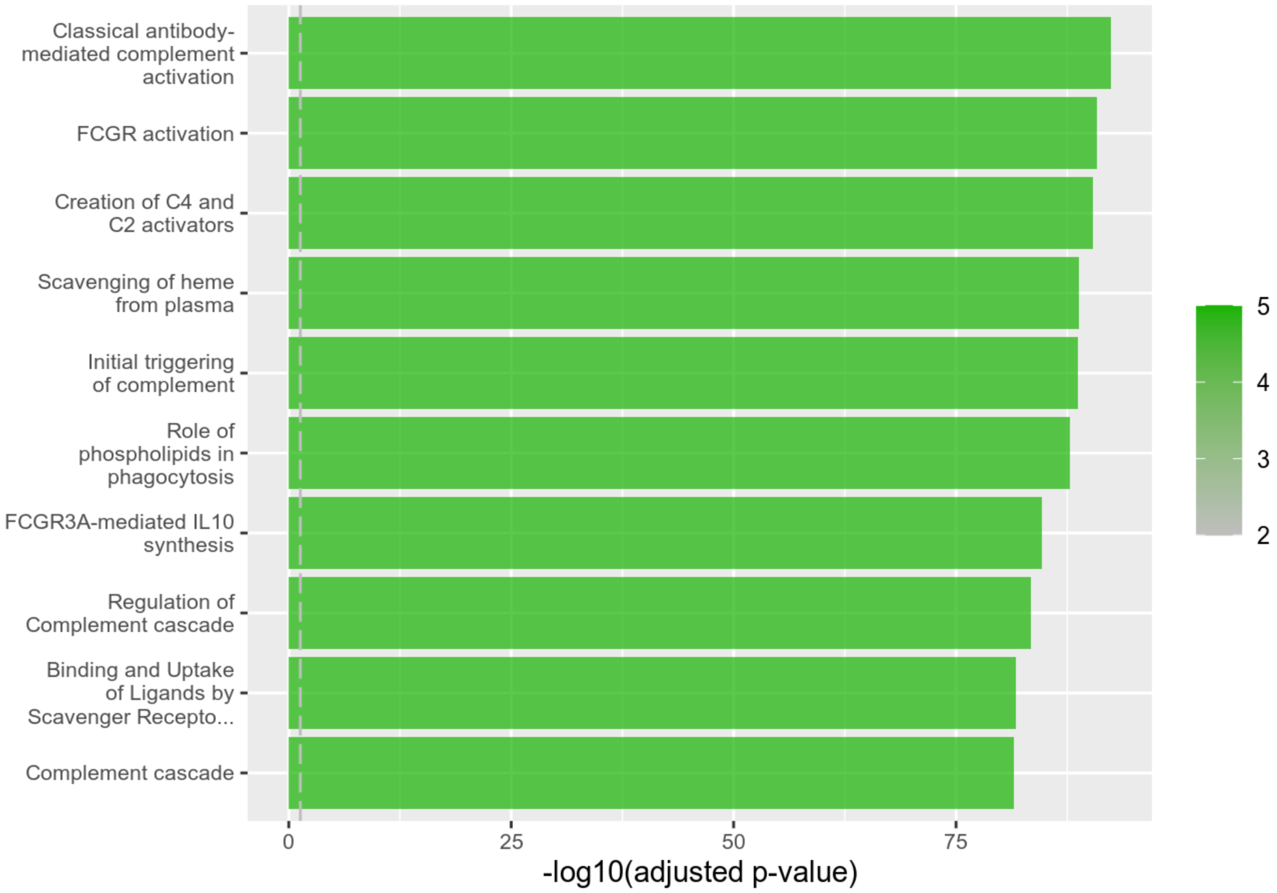

Classical antibody-mediated complement activation
FCGR activation
Creation of C4 and C2 activators
Scavenging of heme from plasma
Initial triggering of complement
Role of phospholipids in phagocytosis
FCGR3A-mediated IL10 synthesis
Regulation of Complement cascade
Binding and Uptake of Ligands by Scavenger Recepto...
Complement cascade
0
25
50
75
-log10(adjusted p-value)
5
4
3
2

M5

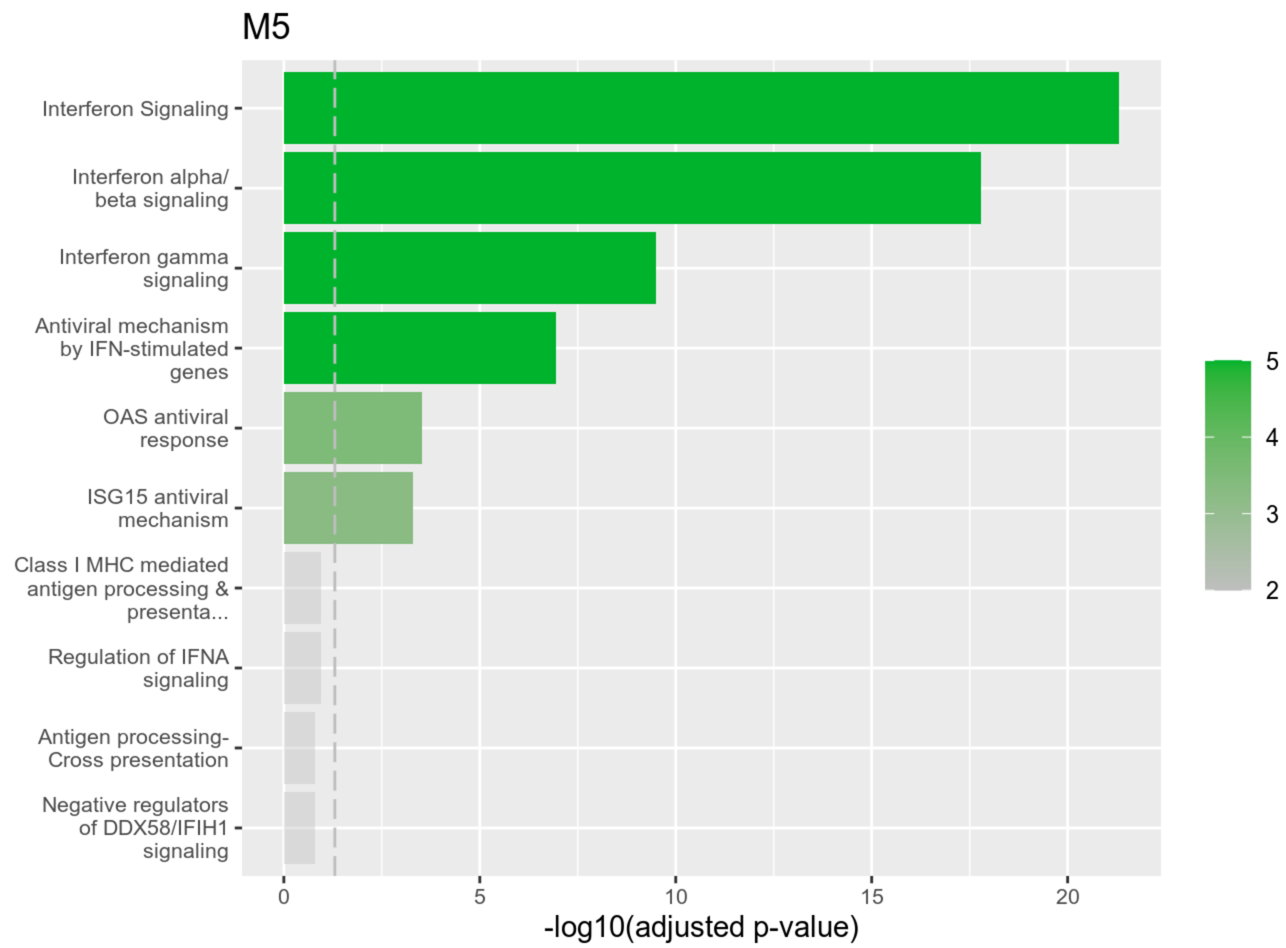
M5
Interferon Signaling
Interferon alpha/ beta signaling
Interferon gamma signaling
Antiviral mechanism by IFN-stimulated genes
OAS antiviral response
ISG15 antiviral mechanism
Class I MHC mediated antigen processing & presenta...
Regulation of IFNA signaling
Antigen processing-Cross presentation
Negative regulators of DDX58/IFIH1 signaling
0
5
10
15
20
-log10(adjusted p-value)
5
4
3
2

M6

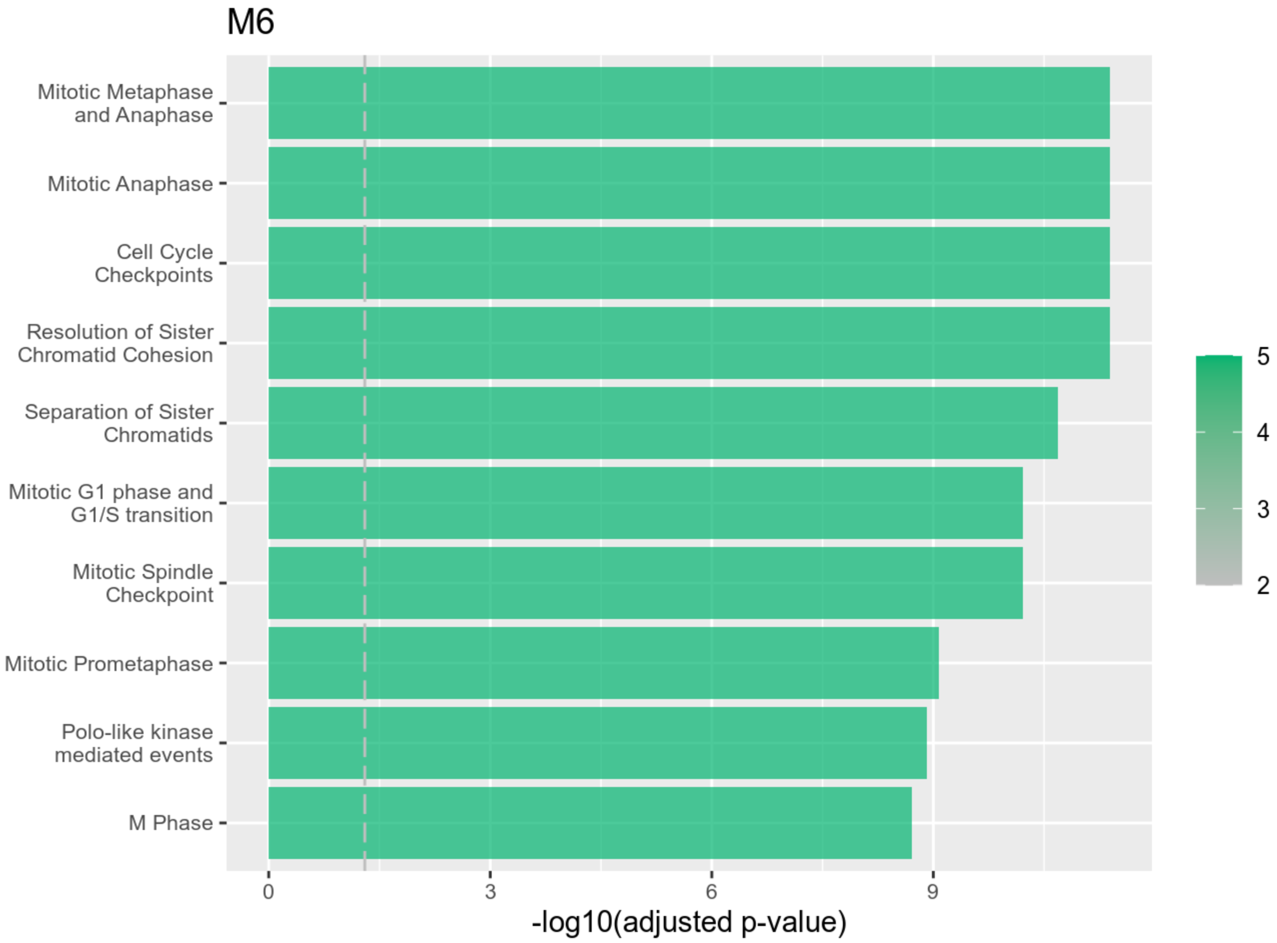
M6
Mitotic Metaphase and Anaphase
Mitotic Anaphase
Cell Cycle Checkpoints
Resolution of Sister Chromatid Cohesion
Separation of Sister Chromatids
Mitotic G1 phase and G1/S transition
Mitotic Spindle Checkpoint
Mitotic Prometaphase
Polo-like kinase mediated events
M Phase
0
3
6
9
-log10(adjusted p-value)
5
4
3
2

M7

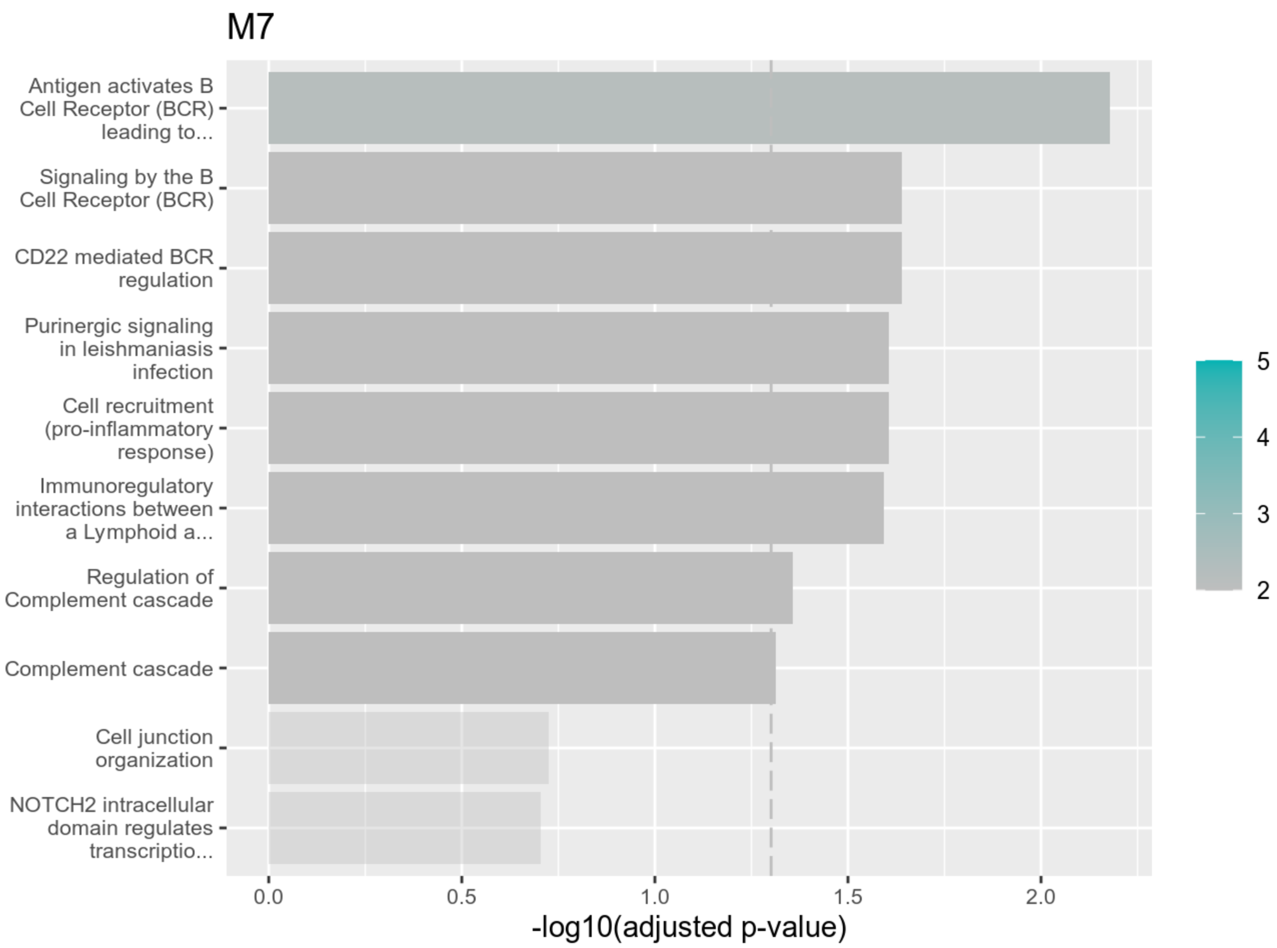

M7
Antigen activates B Cell Receptor (BCR) leading to...
Signaling by the B Cell Receptor (BCR)
CD22 mediated BCR regulation
Purinergic signaling in leishmaniasis infection
Cell recruitment (pro-inflammatory response)
Immunoregulatory interactions between a Lymphoid a...
Regulation of Complement cascade
Complement cascade
Cell junction organization
NOTCH2 intracellular domain regulates transcriptio...
0.0
0.5
1.0
1.5
2.0
-log10(adjusted p-value)
5
4
3
2

M8

M8

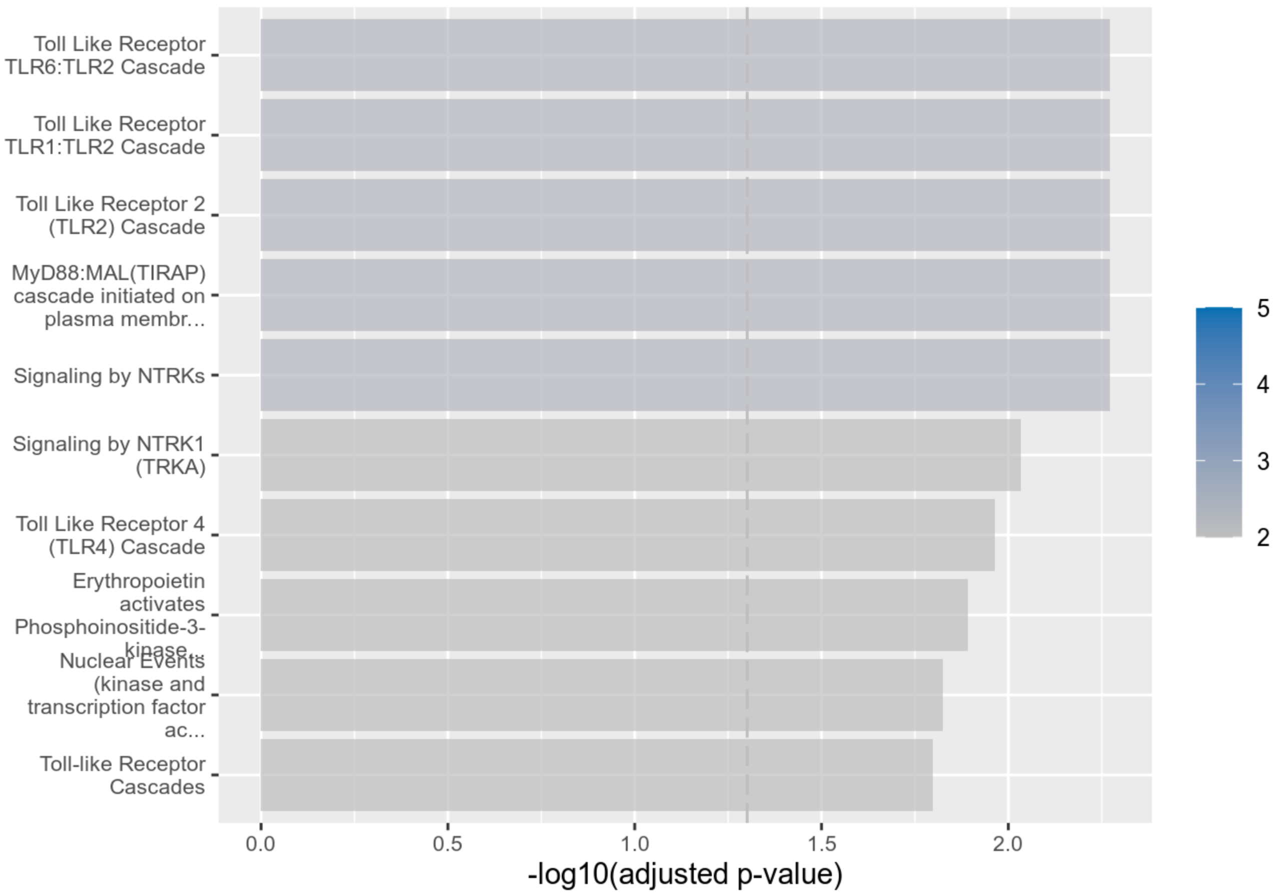

Toll Like Receptor TLR6:TLR2 Cascade
Toll Like Receptor TLR1:TLR2 Cascade
Toll Like Receptor 2 (TLR2) Cascade
MyD88:MAL(TIRAP) cascade initiated on plasma membr...
Signaling by NTRKs
Signaling by NTRK1 (TRKA)
Toll Like Receptor 4 (TLR4) Cascade
Erythropoietin activates Phosphoinositide-3-kinase...
Nuclear Events (kinase and transcription factor ac...
Toll-like Receptor Cascades
0.0
0.5
1.0
1.5
2.0
-log10(adjusted p-value)
5
4
3
2

M9

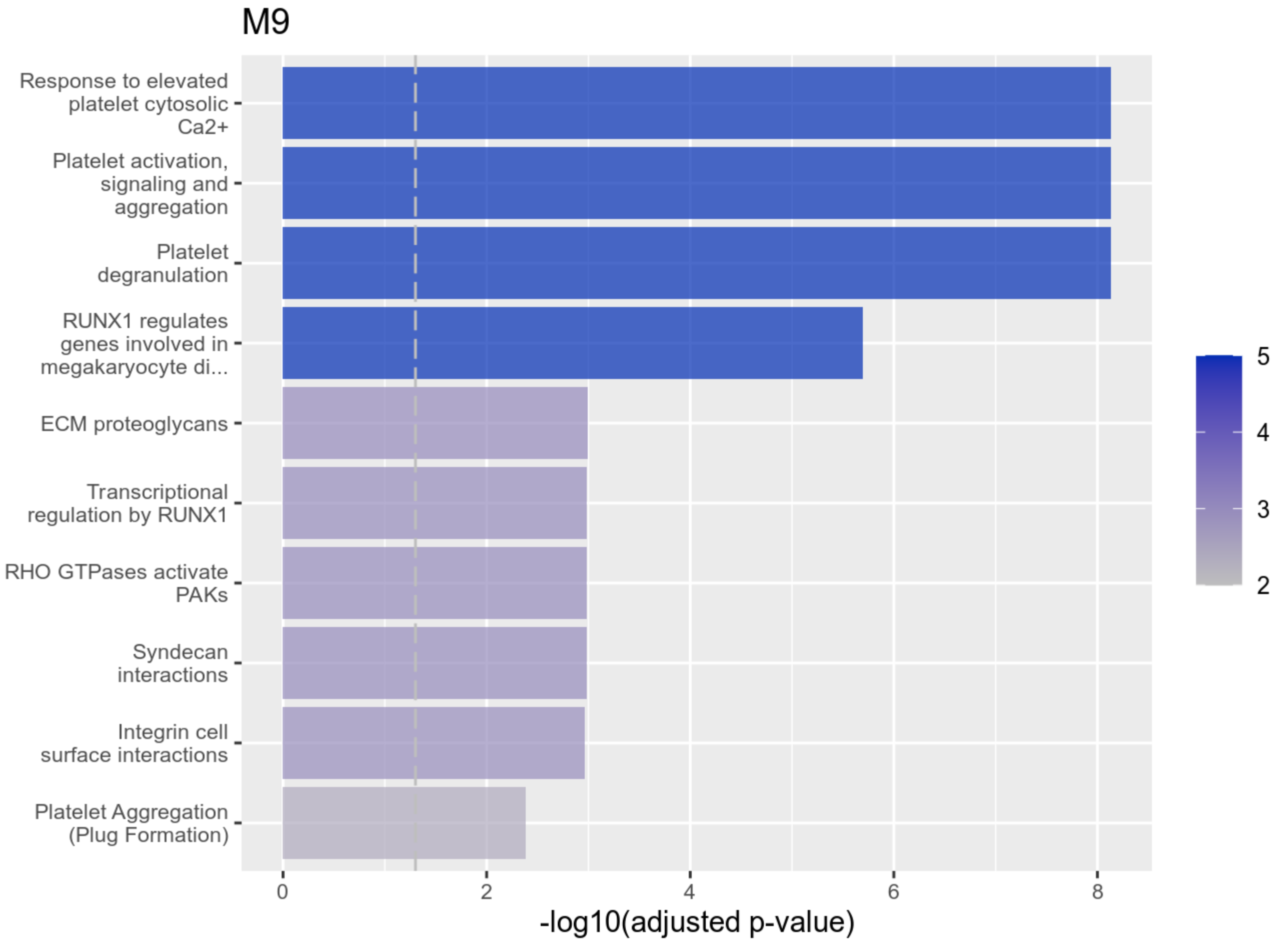
M9
Response to elevated platelet cytosolic Ca2+
Platelet activation, signaling and aggregation
Platelet degranulation
RUNX1 regulates genes involved in megakaryocyte di...
ECM proteoglycans
Transcriptional regulation by RUNX1
RHO GTPases activate PAKs
Syndecan interactions
Integrin cell surface interactions
Platelet Aggregation (Plug Formation)
0
2
4
6
8
-log10(adjusted p-value)
5
4
3
2

M10

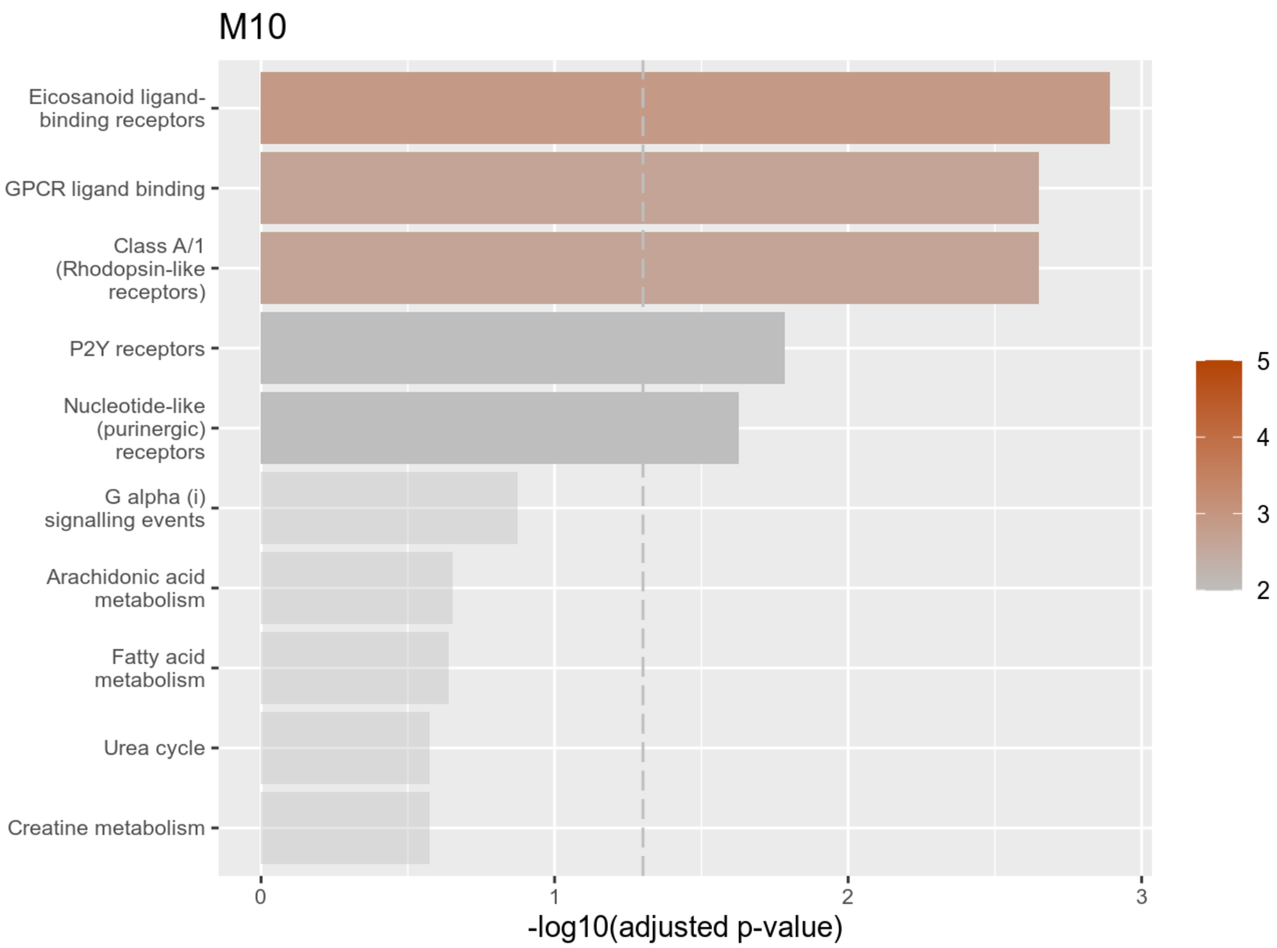
M10
Eicosanoid ligand-binding receptors
GPCR ligand binding
Class A/1 (Rhodopsin-like receptors)
P2Y receptors
Nucleotide-like (purinergic) receptors
G alpha (i) signalling events
Arachidonic acid metabolism
Fatty acid metabolism
Urea cycle
Creatine metabolism
0
1
2
3
-log10(adjusted p-value)
5
4
3
2

M11

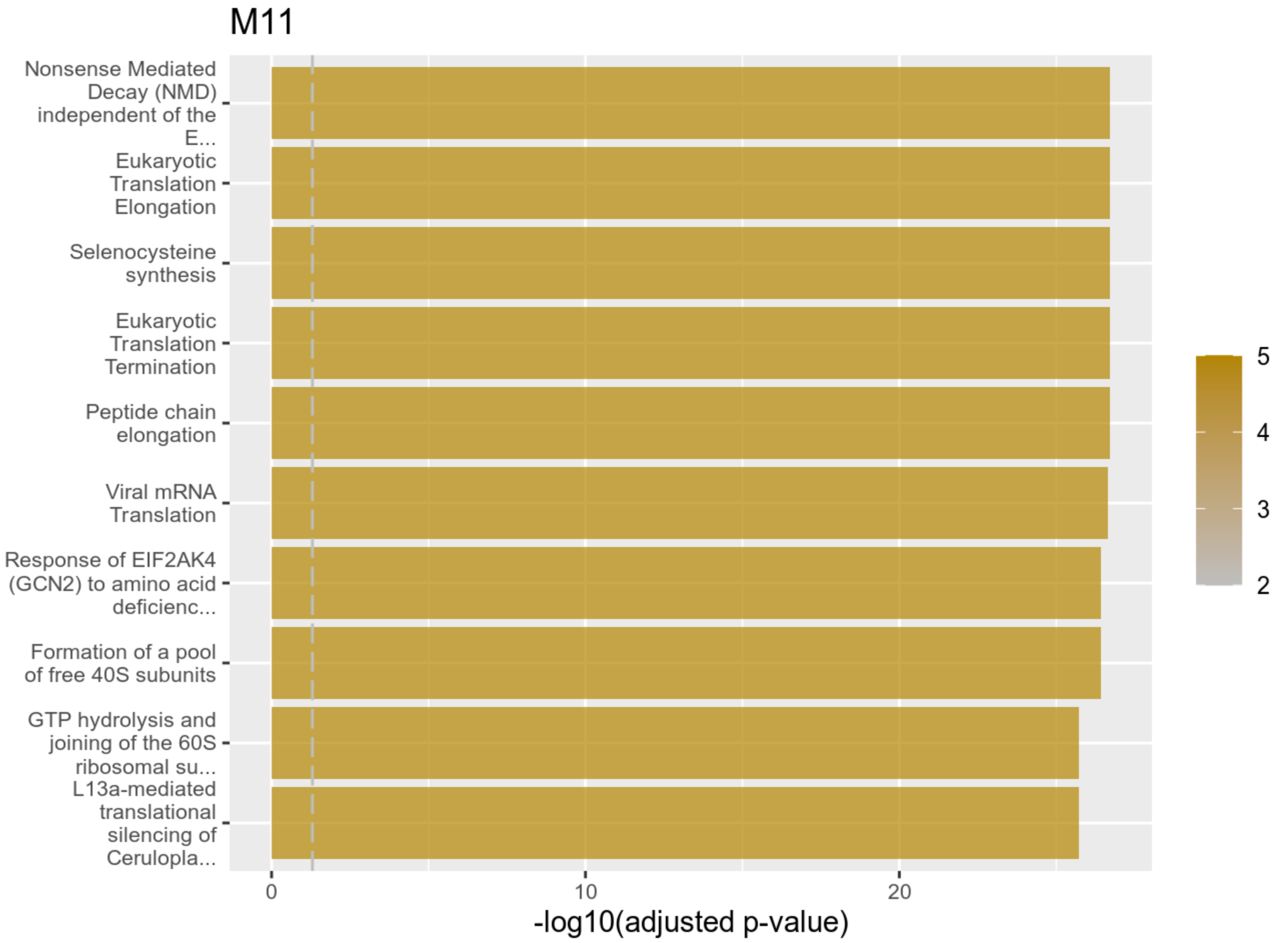

Not.Correlated

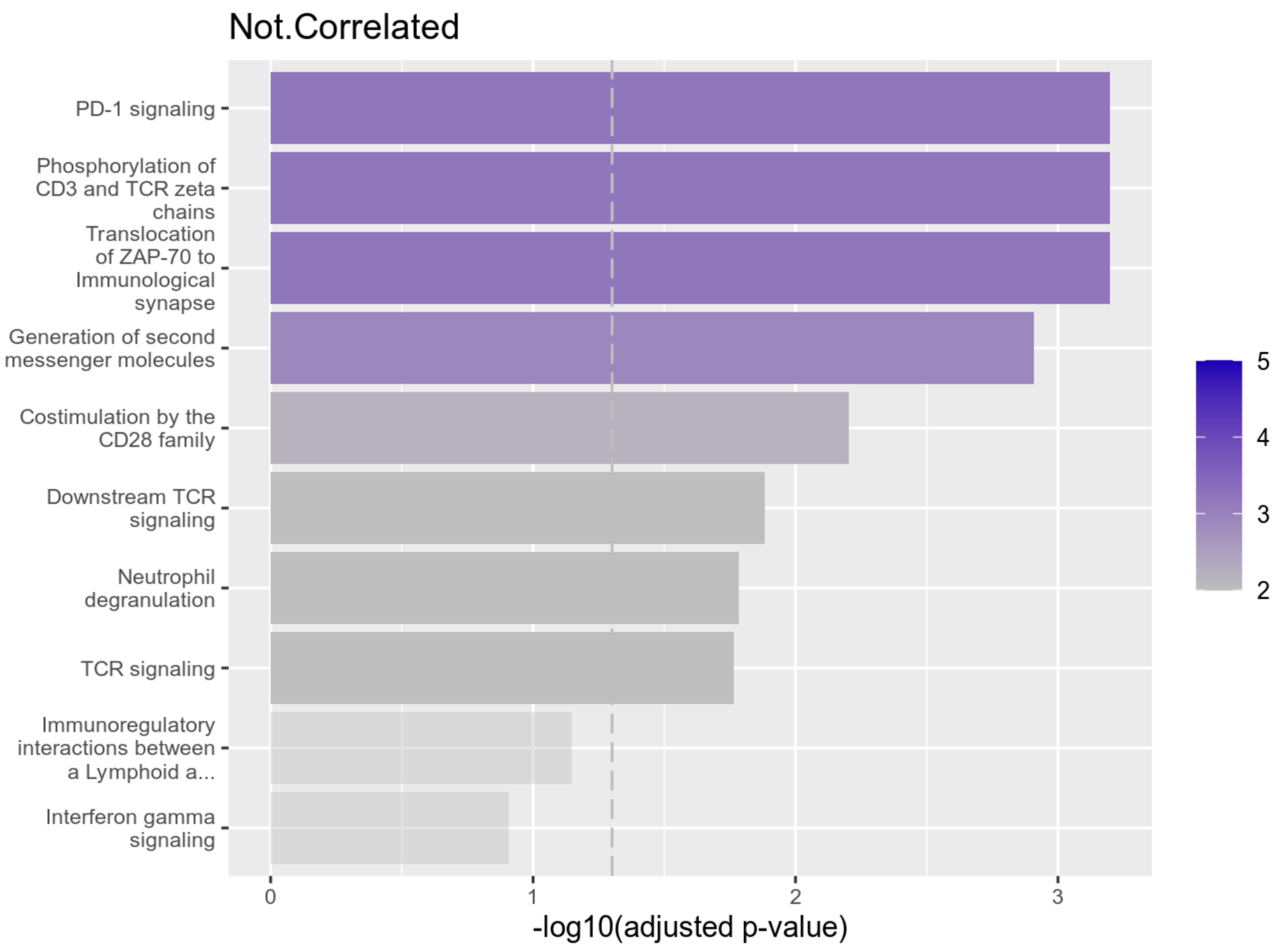

Interaction Network

M1

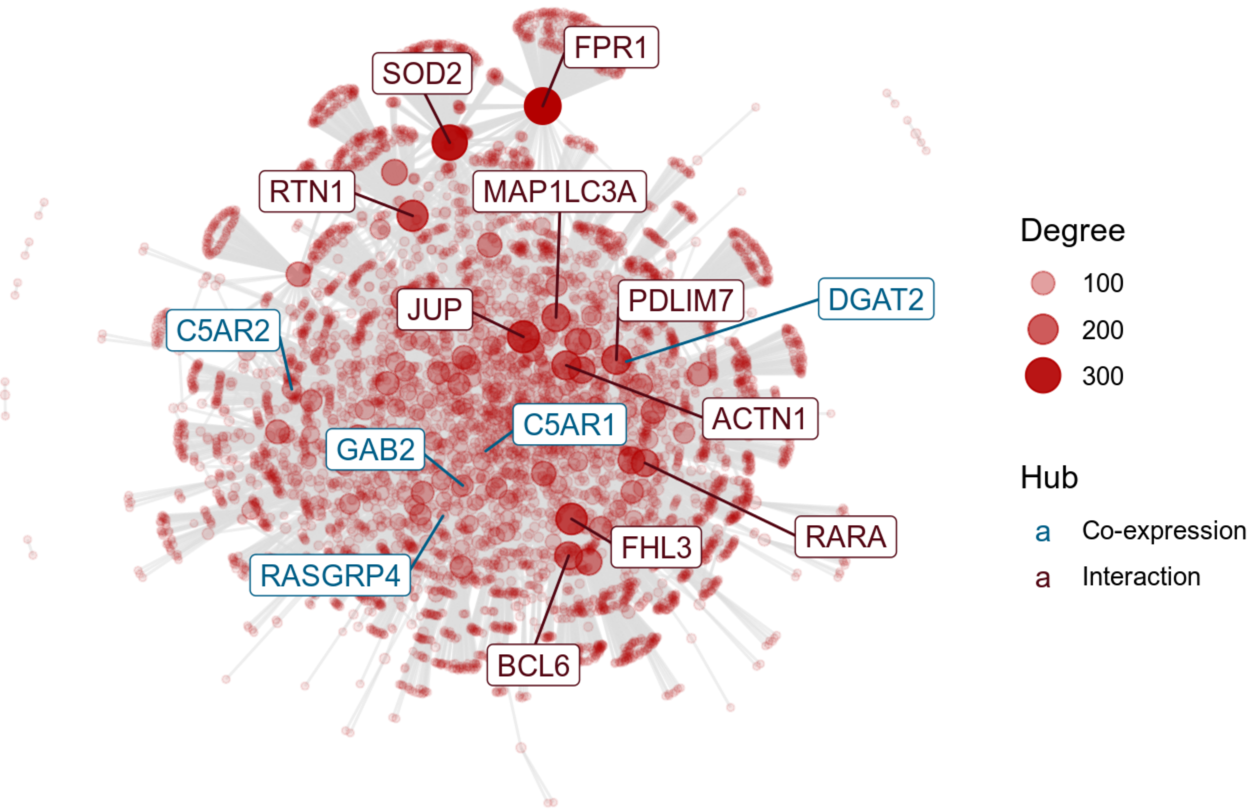
FPR1
SOD2
RTN1
MAP1LC3A
JUP
PDLIM7
DGAT2
C5AR2
ACTN1
C5AR1
GAB2
RARA
FHL3
RASGRP4
BCL6
Degree
100
200
300
Hub
a Co-expression
a Interaction

M2

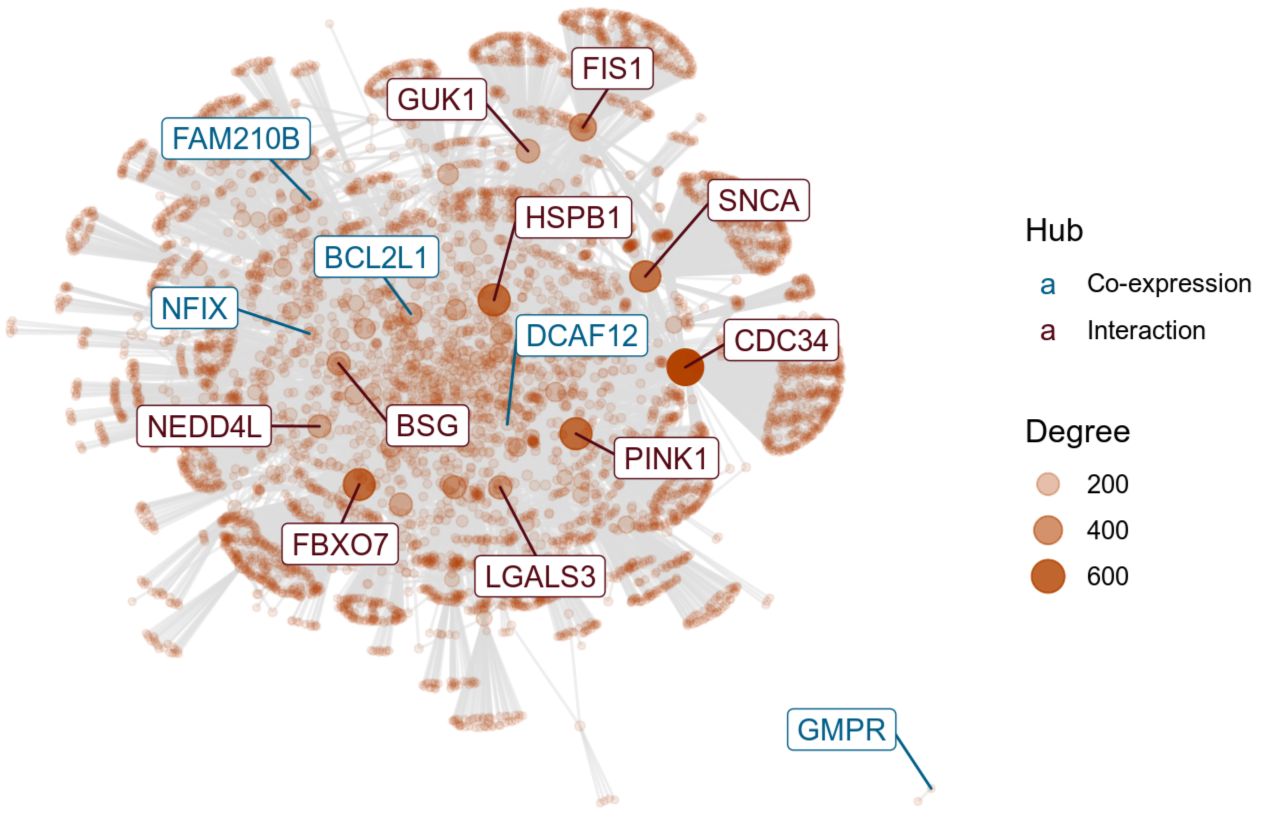
FIS1
GUK1
FAM210B
HSPB1
SNCA
BCL2L1
NFIX
DCAF12
CDC34
NEDD4L
BSG
PINK1
FBXO7
LGALS3
GMPR
Hub
a Co-expression
a Interaction
Degree
200
400
600

M3

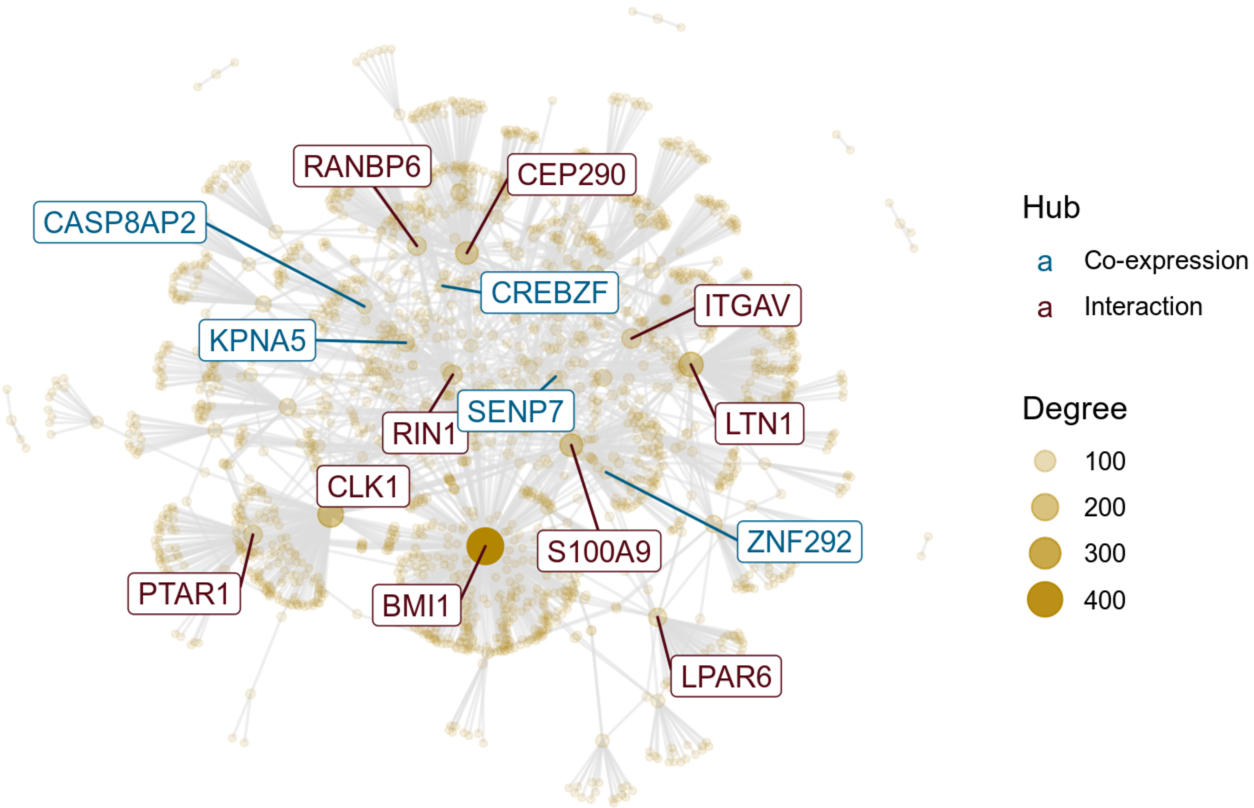
RANBP6
CEP290
CASP8AP2
CREBZF
ITGAV
KPNA5
LTN1
SENP7
RIN1
CLK1
S100A9
ZNF292
PTAR1
BMI1
LPAR6
Hub
a Co-expression
a Interaction
Degree
100
200
300
400

M4

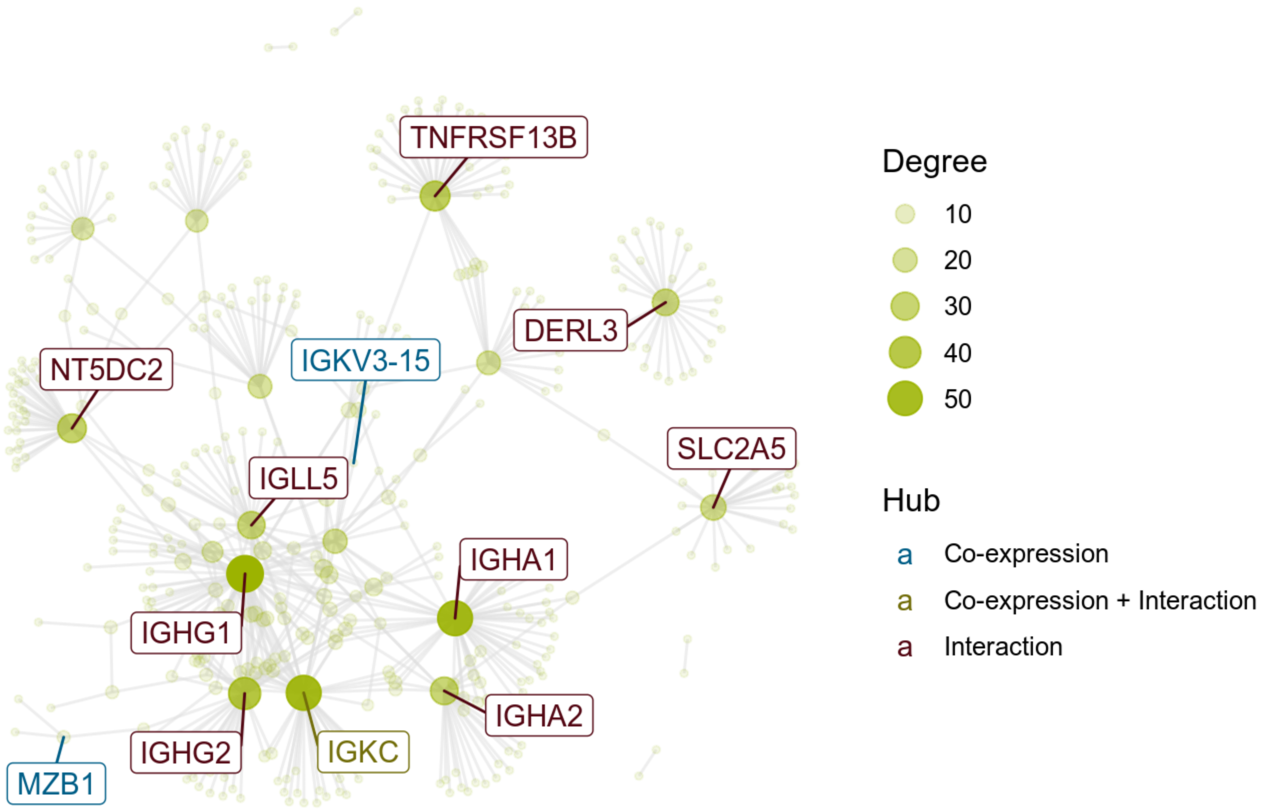
TNFRSF13B
DERL3
NT5DC2
IGKV3-15
IGLL5
SLC2A5
IGHA1
IGHG1
IGHA2
IGHG2
IGKC
MZB1
Degree
10
20
30
40
50
Hub
a Co-expression
a Co-expression + Interaction
a Interaction

M5

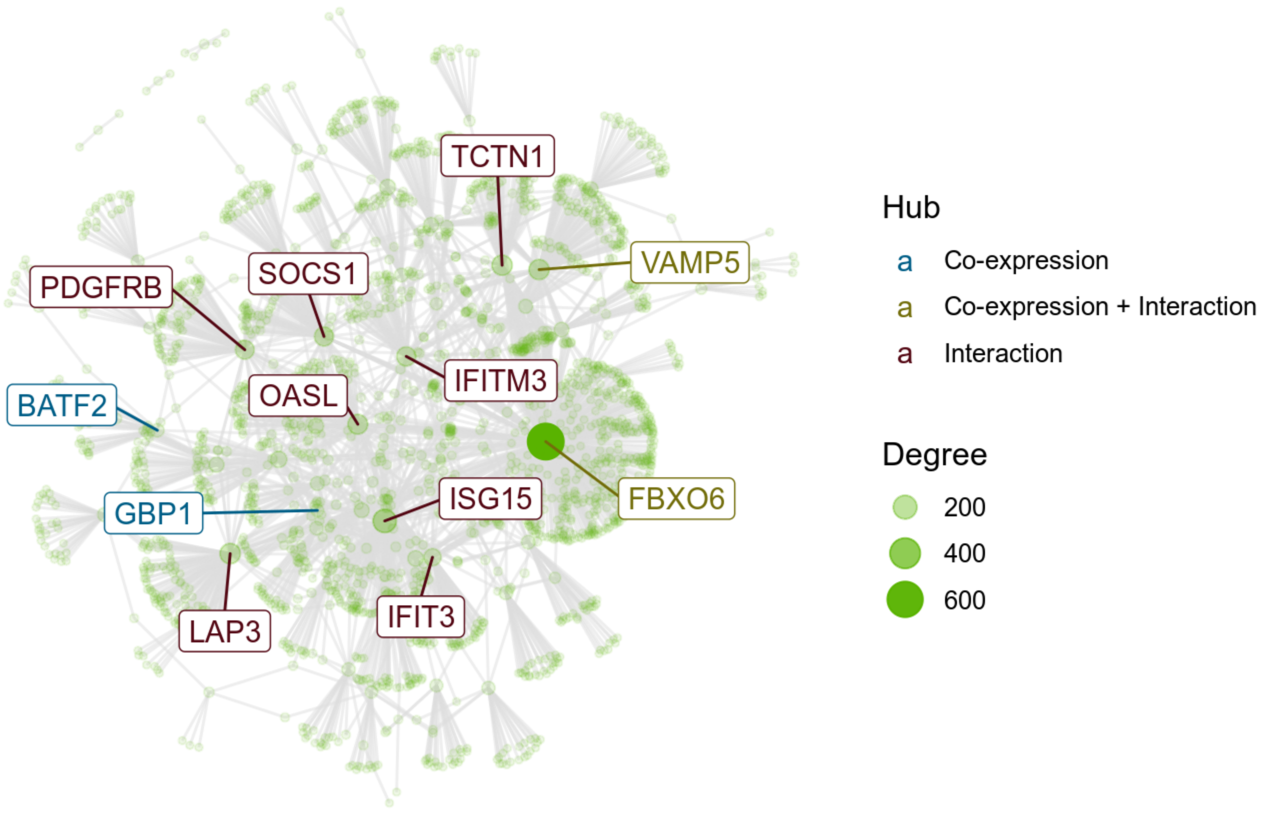
TCTN1
VAMP5
PDGFRB
SOCS1
IFITM3
BATF2
OASL
FBXO6
GBP1
ISG15
LAP3
IFIT3
Hub
a Co-expression
a Co-expression + Interaction
a Interaction
Degree
200
400
600

M6

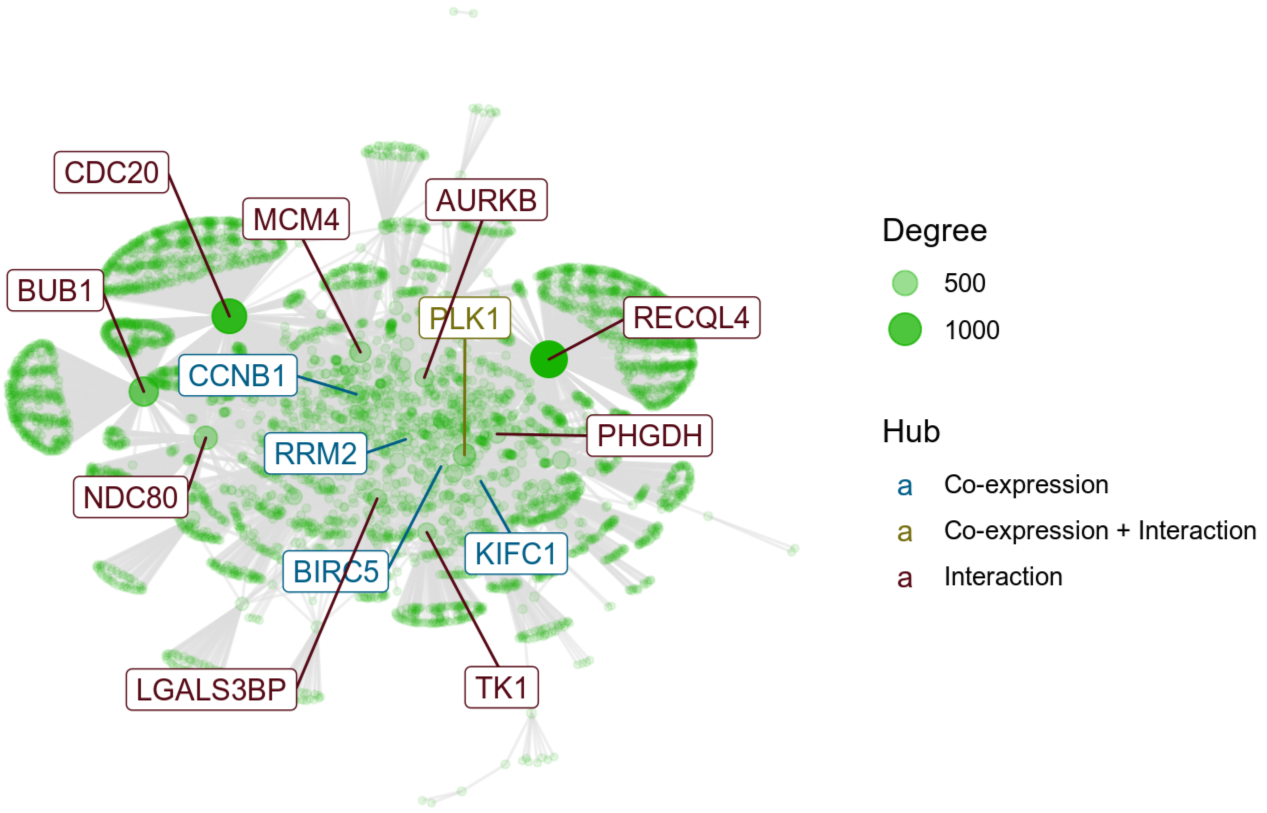
CDC20
MCM4
AURKB
BUB1
PLK1
RECQL4
CCNB1
RRM2
PHGDH
NDC80
KIFC1
BIRC5
LGALS3BP
TK1
Degree
500
1000
Hub
a Co-expression
a Co-expression + Interaction
a Interaction

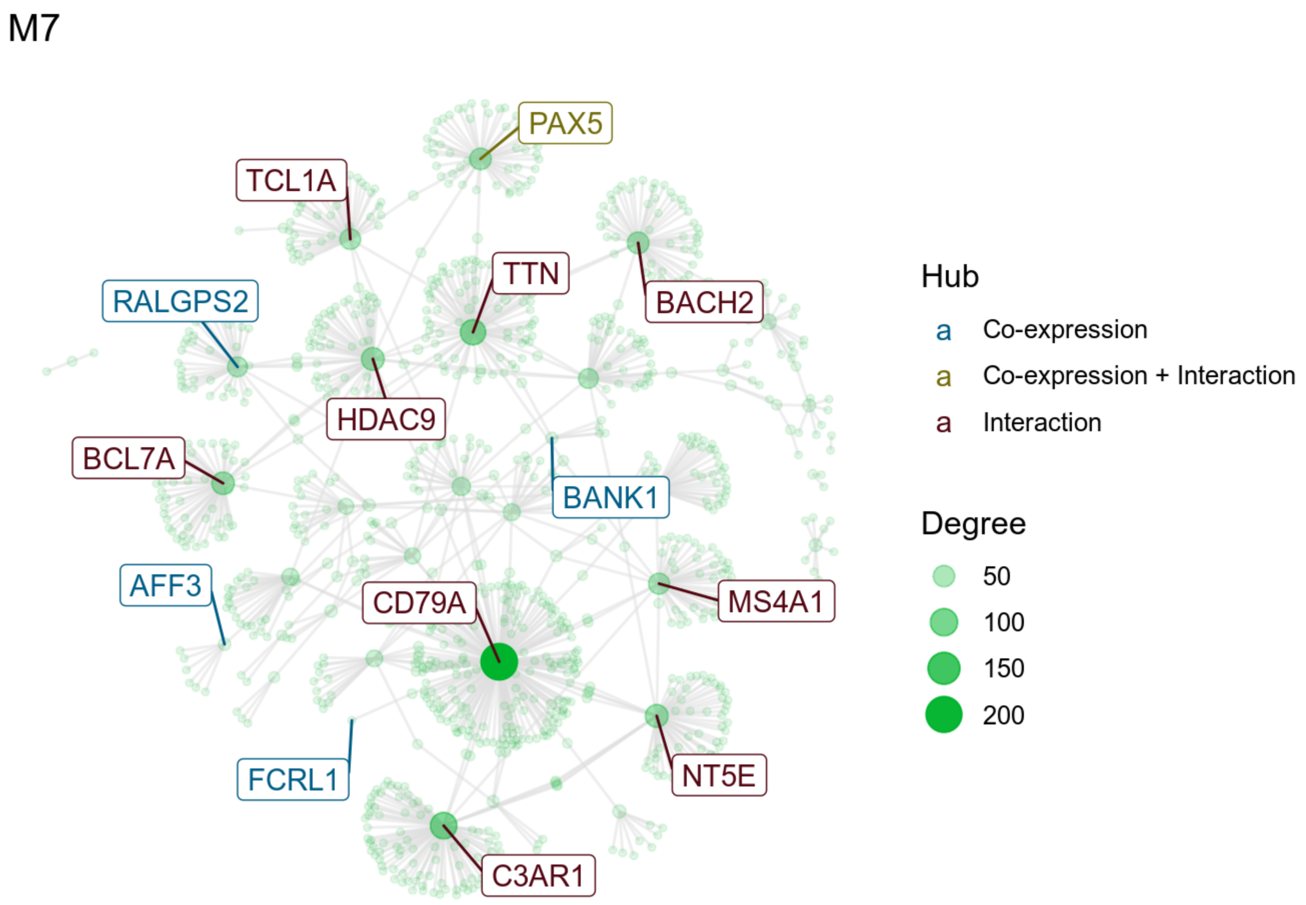
M7
PAX5
TCL1A
TTN
BACH2
RALGPS2
HDAC9
BCL7A
BANK1
AFF3
CD79A
MS4A1
FCRL1
NT5E
C3AR1
Hub
a Co-expression
a Co-expression + Interaction
a Interaction
Degree
50
100
150
200

M8

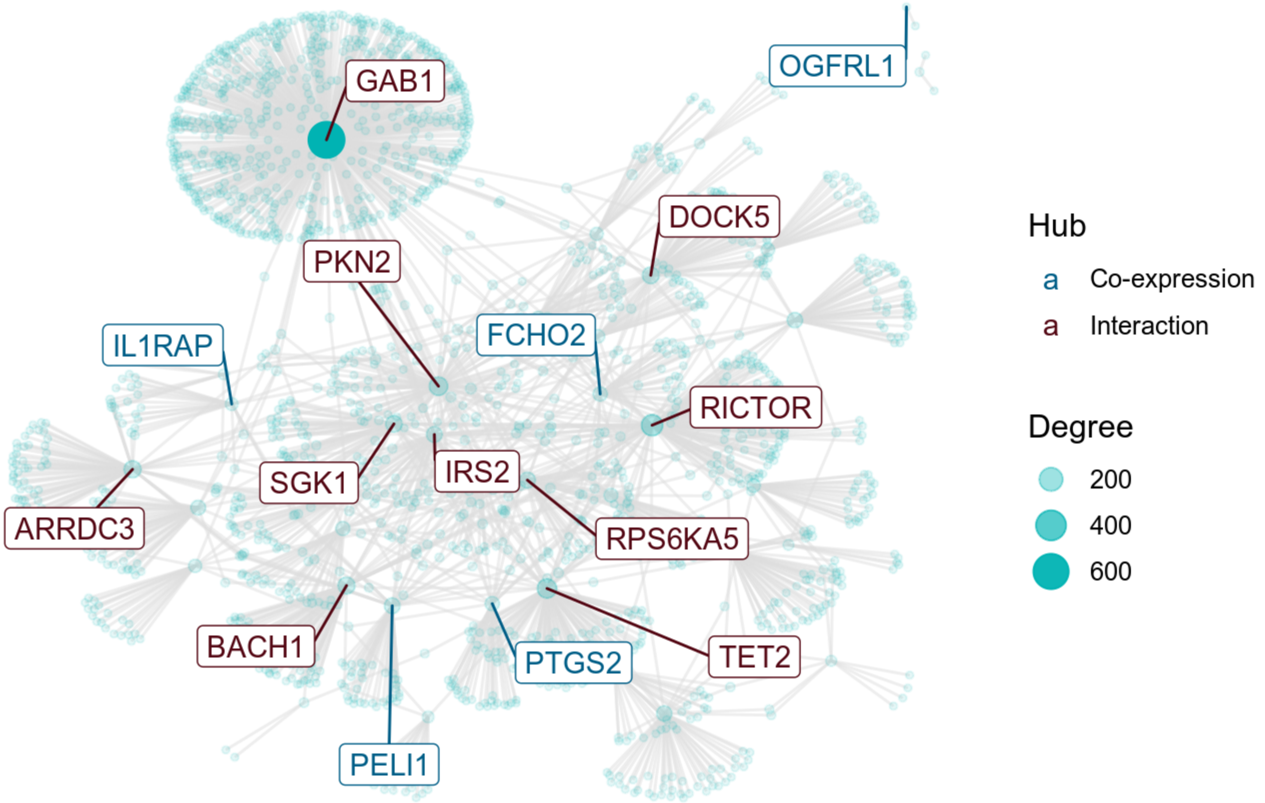
GAB1
OGFRL1
DOCK5
PKN2
IL1RAP
FCHO2
RICTOR
SGK1
IRS2
ARRDC3
RPS6KA5
BACH1
TET2
PTGS2
PELI1
Hub
a Co-expression
a Interaction
Degree
200
400
600

M9

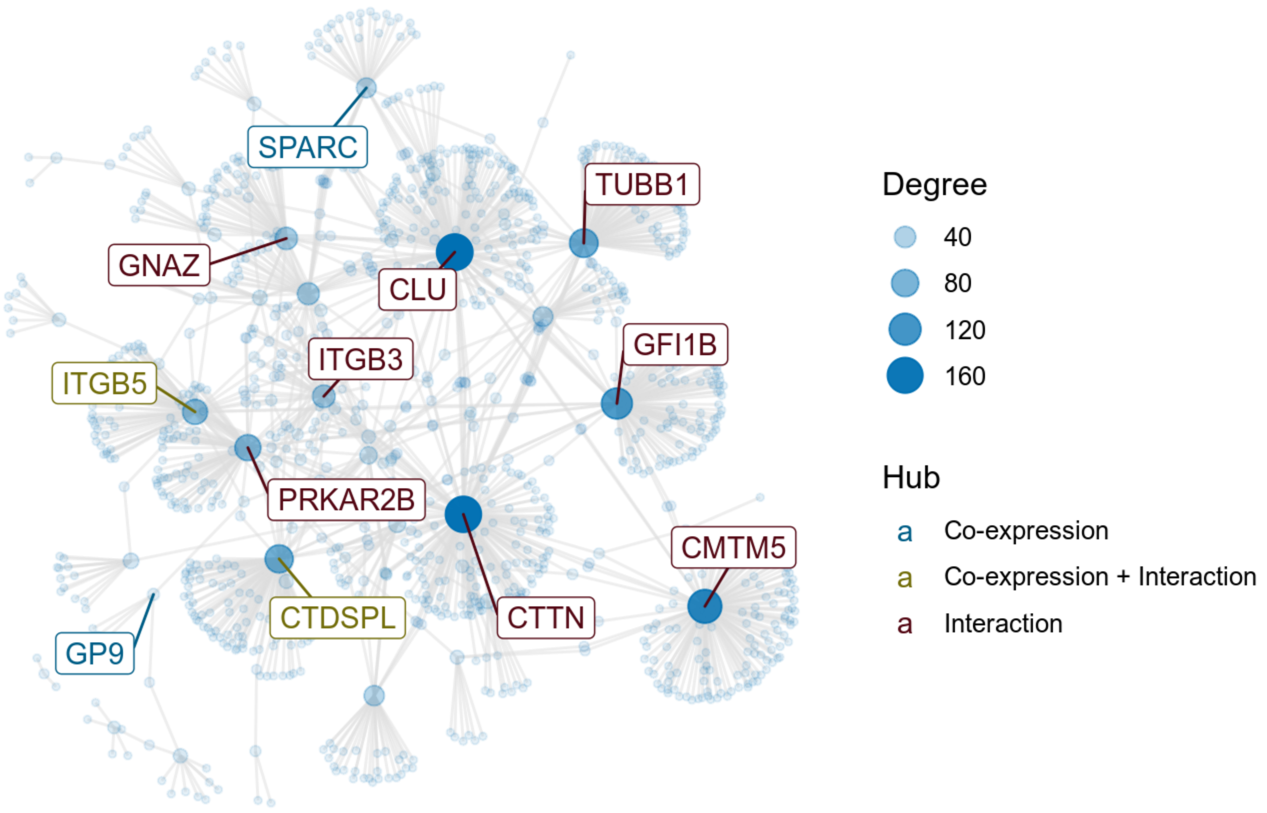
SPARC
TUBB1
GNAZ
CLU
GFI1B
ITGB3
ITGB5
PRKAR2B
CMTM5
CTDSPL
CTTN
GP9
Degree
40
80
120
160
Hub
a Co-expression
a Co-expression + Interaction
a Interaction

M10

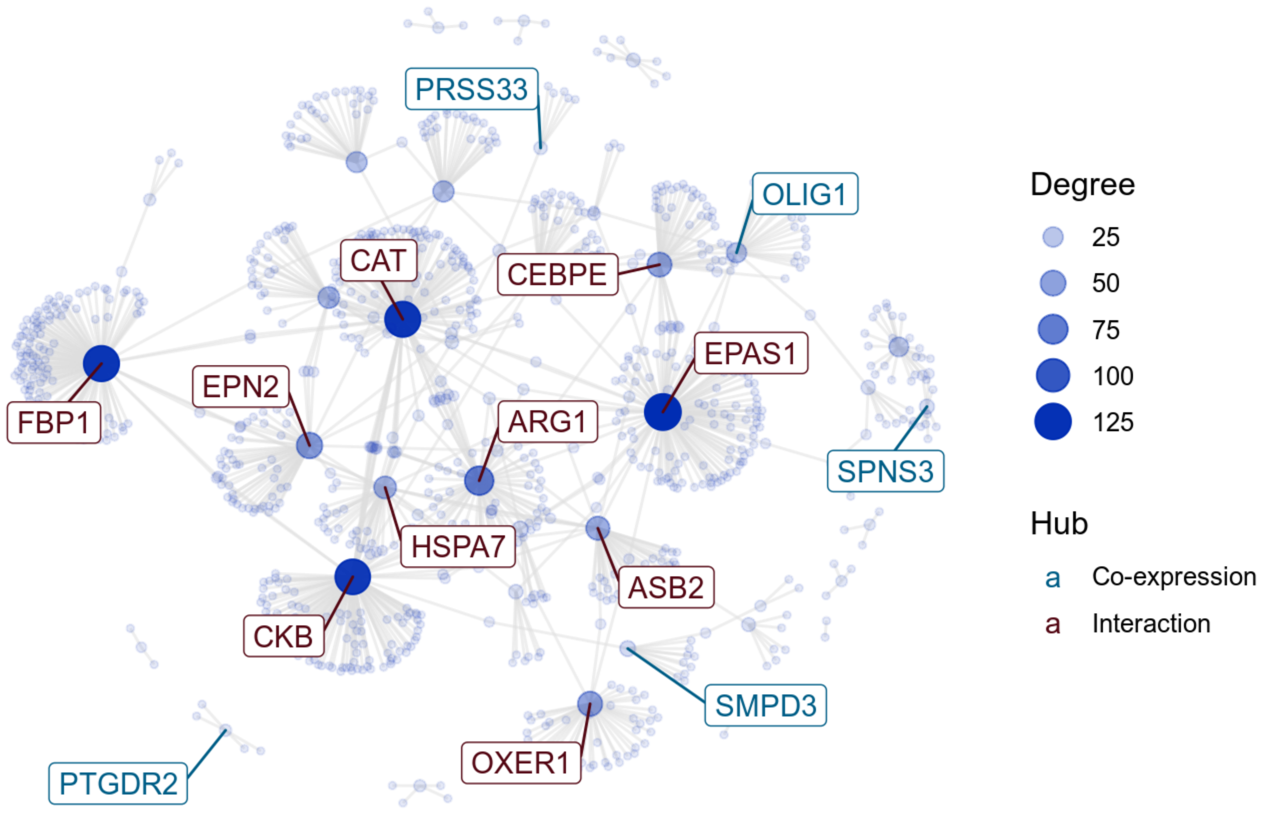
PRSS33
OLIG1
CAT
CEBPE
EPAS1
FBP1
EPN2
ARG1
SPNS3
HSPA7
ASB2
CKB
SMPD3
OXER1
PTGDR2
Degree
25
50
75
100
125
Hub
a Co-expression
a Interaction

M11

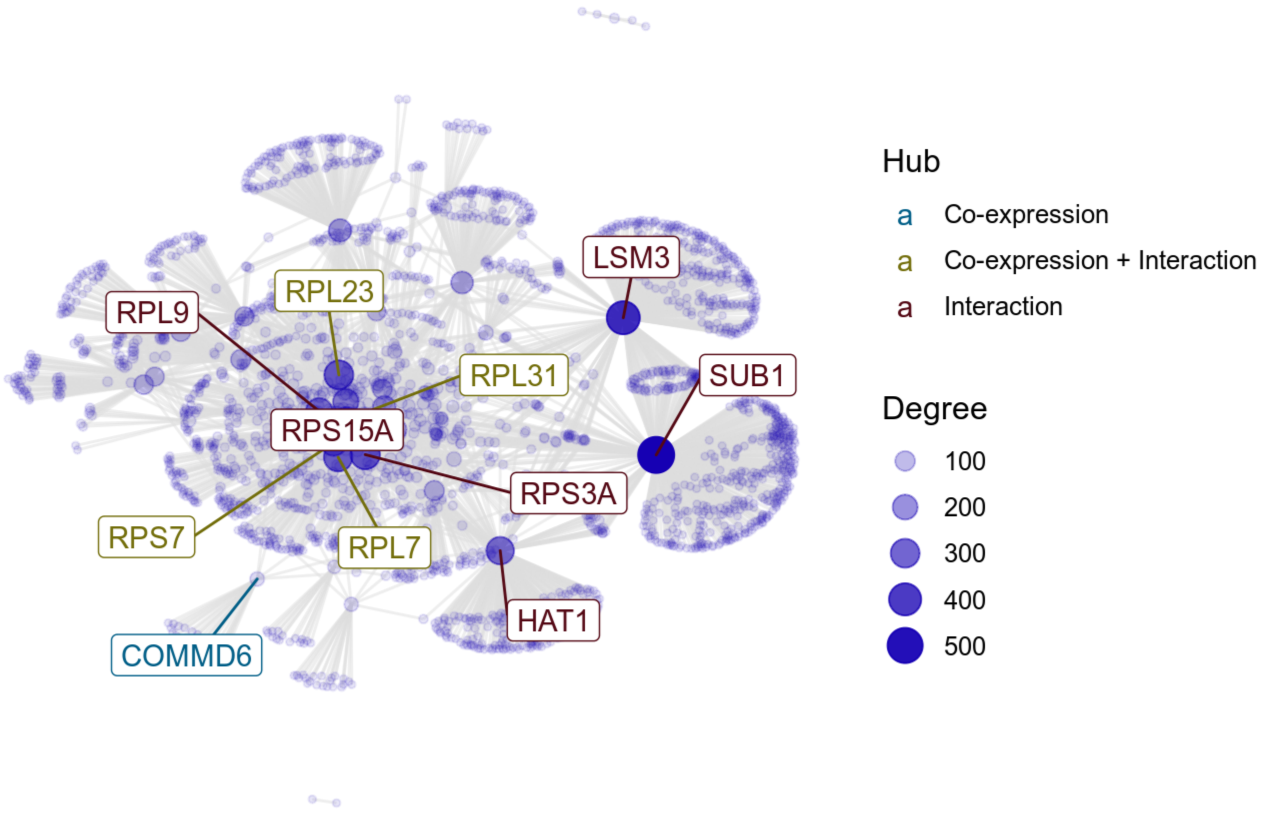

Parameters
