# Supplementary figures and images for: Insight Into the Long Noncoding RNA and mRNA Coexpression Profile in the Human Blood Transcriptome Upon Leishmania infantum Infection
Source: Front Immunol. 2022 Mar 15;13:784463. doi: 10.3389/fimmu.2022.784463 (PMC8965071; doi:10.3389/fimmu.2022.784463)

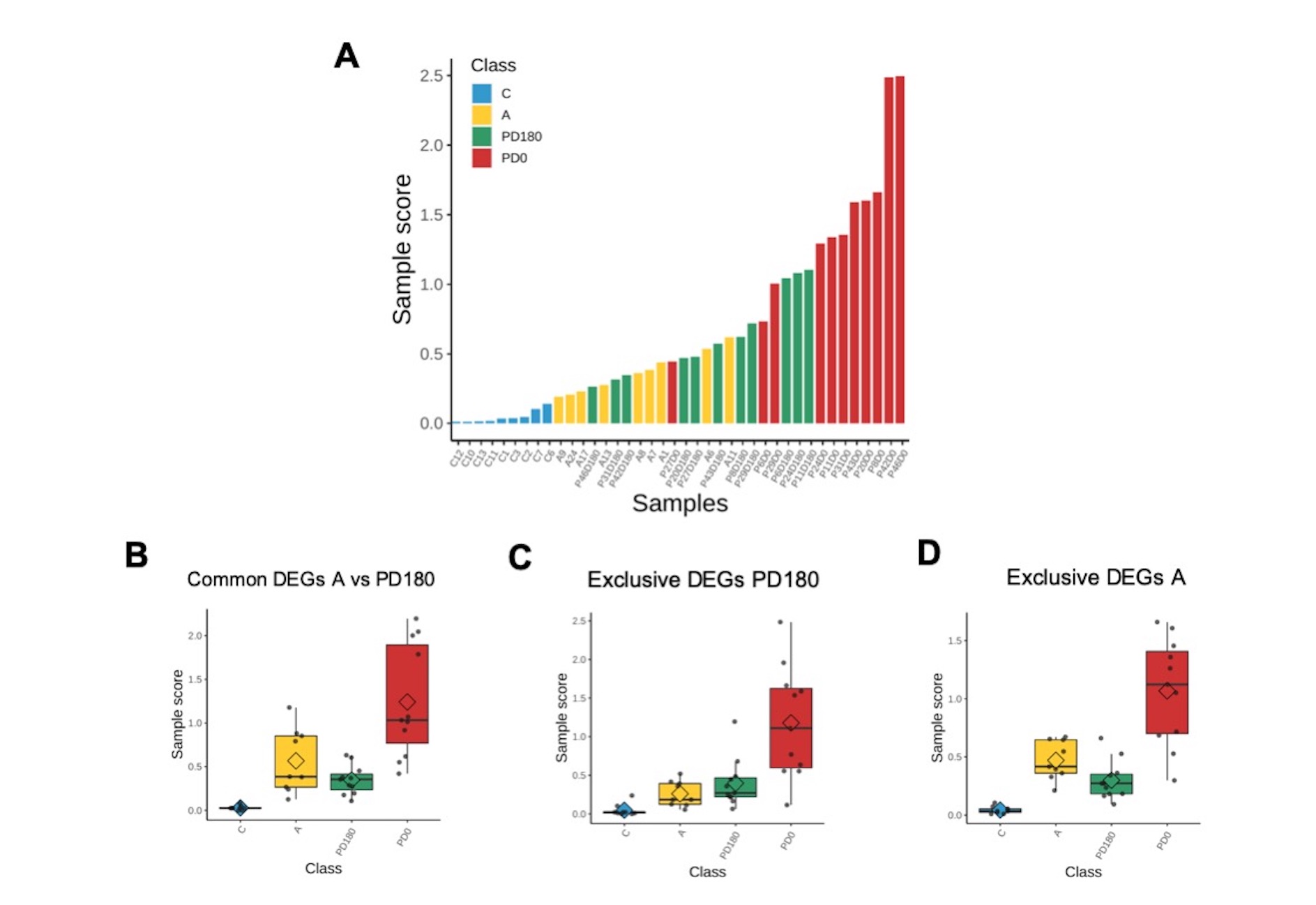

Supplement: Supplementary Figure 1 — Heterogeneity of samples analyzed using the Molecular Degree of Perturbation (MDP) tool. (A) Molecular perturbation scores plotted for each sample based on 14,247 genes expressed across the four groups. DEGs shared among all comparisons of VL patients to nondiseased groups (as numbers in (Figure 3) , central overlap); (B–D) Boxplots representing MDP scores by group quantified based on three subsets of DEGs, where the first was composed of 84 DEGs shared between A vs. PD0 and PD180 vs. PD0 comparisons (b), the second was composed of 186 DEGs exclusive for PD180 vs. PD0 comparison (c) and the third was composed of 193 DEGs exclusive for A vs. PD0 comparison. [file Image_1.jpeg]

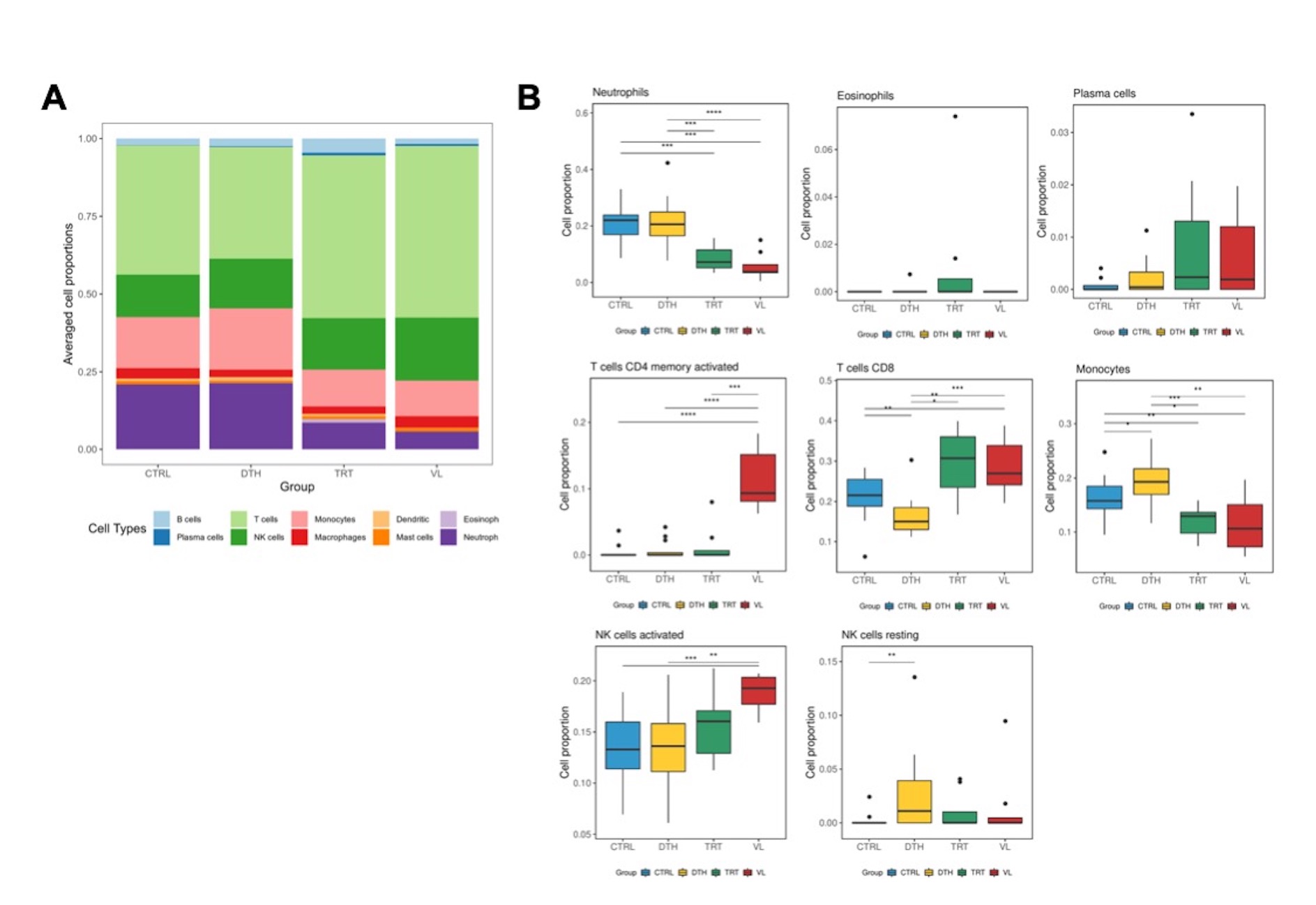

Supplement: Supplementary Figure 2 — Cellular deconvolution of blood transcriptomes in human visceral leishmaniasis using the CIBERSORT method. (A) Leukocyte proportions inferred from gene expression profiles of blood samples from Gardinassi et al., 2016 (microarray). (B) Plots by cell type displaying the relative cell proportions for neutrophils, eosinophils, plasma cells, memory CD4 cell plasma cells, CD8 T cells, monocytes, activated NK cells and resting NK cells. In the study of Gardinassi et al., groups were classified as CTRL (healthy uninfected controls, n= 15), DTH (asymptomatic patients, n= 14), TRT (patients at 2-5 months after treatment, considered under remission of the disease; n= 8) and VL (diseased patients, n= 8). [file Image_2.jpeg]
